# Clonal Evolutionary Analysis during HER2 Blockade in HER2-Positive Inflammatory Breast Cancer: A Phase II Open-Label Clinical Trial of Afatinib +/- Vinorelbine

**DOI:** 10.1371/journal.pmed.1002136

**Published:** 2016-12-06

**Authors:** Gerald Goh, Ramona Schmid, Kelly Guiver, Wichit Arpornwirat, Imjai Chitapanarux, Vinod Ganju, Seock-Ah Im, Sung-Bae Kim, Arunee Dechaphunkul, Jedzada Maneechavakajorn, Neil Spector, Thomas Yau, Mehdi Afrit, Slim Ben Ahmed, Stephen R. Johnston, Neil Gibson, Martina Uttenreuther-Fischer, Javier Herrero, Charles Swanton

**Affiliations:** 1 Translational Cancer Therapeutics Laboratory, UCL Cancer Institute, London, United Kingdom; 2 Bill Lyons Informatics Centre, UCL Cancer Institute, London, United Kingdom; 3 Boehringer Ingelheim Pharma GmbH & Co.KG, Biberach, Germany; 4 Boehringer Ingelheim Ltd, Bracknell, United Kingdom; 5 National Cancer Institute, Bangkok, Thailand; 6 Maharaj Nakhon Chiang Mai Hospital, Chiang Mai, Thailand; 7 Monash Medical Centre, Melbourne, Australia; 8 Seoul National University Hospital, Seoul, South Korea; 9 Asan Medical Center, University of Ulsan College of Medicine, Ulsan, South Korea; 10 Prince of Songkla University, Songkhla, Thailand; 11 Rajavithi Hospital, Bangkok, Thailand; 12 Duke University Medical Center, Durham, North Carolina, United States of America; 13 Queen Mary Hospital, Hong Kong; 14 Abderrahman Mami Hospital, Ariana, Tunisia; 15 Fahat Hached Hospital, Sousse, Tunisia; 16 Royal Marsden Hospital Breast Unit, London, United Kingdom; 17 The Francis Crick Institute, London, United Kingdom; Harvard Medical School, UNITED STATES

## Abstract

**Background:**

Inflammatory breast cancer (IBC) is a rare, aggressive form of breast cancer associated with HER2 amplification, with high risk of metastasis and an estimated median survival of 2.9 y. We performed an open-label, single-arm phase II clinical trial (ClinicalTrials.gov NCT01325428) to investigate the efficacy and safety of afatinib, an irreversible ErbB family inhibitor, alone and in combination with vinorelbine in patients with HER2-positive IBC. This trial included prospectively planned exome analysis before and after afatinib monotherapy.

**Methods and Findings:**

HER2-positive IBC patients received afatinib 40 mg daily until progression, and thereafter afatinib 40 mg daily and intravenous vinorelbine 25 mg/m^2^ weekly. The primary endpoint was clinical benefit; secondary endpoints were objective response (OR), duration of OR, and progression-free survival (PFS). Of 26 patients treated with afatinib monotherapy, clinical benefit was achieved in 9 patients (35%), 0 of 7 trastuzumab-treated patients and 9 of 19 trastuzumab-naïve patients. Following disease progression, 10 patients received afatinib plus vinorelbine, and clinical benefit was achieved in 2 of 4 trastuzumab-treated and 0 of 6 trastuzumab-naïve patients. All patients had treatment-related adverse events (AEs). Whole-exome sequencing of tumour biopsies taken before treatment and following disease progression on afatinib monotherapy was performed to assess the mutational landscape of IBC and evolutionary trajectories during therapy. Compared to a cohort of The Cancer Genome Atlas (TCGA) patients with HER2-positive non-IBC, HER2-positive IBC patients had significantly higher mutational and neoantigenic burden, more frequent gain-of-function *TP53* mutations and a recurrent 11q13.5 amplification overlapping *PAK1*. Planned exploratory analysis revealed that trastuzumab-naïve patients with tumours harbouring somatic activation of PI3K/Akt signalling had significantly shorter PFS compared to those without (*p* = 0.03). High genomic concordance between biopsies taken before and following afatinib resistance was observed with stable clonal structures in non-responding tumours, and evidence of branched evolution in 8 of 9 tumours analysed. Recruitment to the trial was terminated early following the LUX-Breast 1 trial, which showed that afatinib combined with vinorelbine had similar PFS and OR rates to trastuzumab plus vinorelbine but shorter overall survival (OS), and was less tolerable. The main limitations of this study are that the results should be interpreted with caution given the relatively small patient cohort and the potential for tumour sampling bias between pre- and post-treatment tumour biopsies.

**Conclusions:**

Afatinib, with or without vinorelbine, showed activity in trastuzumab-naïve HER2-positive IBC patients in a planned subgroup analysis. HER2-positive IBC is characterized by frequent *TP53* gain-of-function mutations and a high mutational burden. The high mutational load associated with HER2-positive IBC suggests a potential role for checkpoint inhibitor therapy in this disease.

**Trial Registration:**

ClinicalTrials.gov NCT01325428

## Abstract 2

In a phase 2 clinical trial in which patients received afatinib, plus vinorelbine on disease progression, Charles Swanton and colleagues study the mutational events in HER2-positive inflammatory breast cancers alongside early-stage clinical outcomes.

## Author Summary

**Why Was This Study Done?:**

- Inflammatory breast cancer (IBC) is a rare and poorly understood form of breast cancer that grows and spreads very quickly. Fifty percent of IBC cases are HER2-positive.
- Afatinib is an investigational drug that showed promise in early-stage trials in the setting of HER2-positive metastatic breast cancer.
- Our study was designed to look at how effective and safe afatinib is in treating HER2-positive IBC patients, and to elucidate how afatinib treatment affects the tumours at the genomic level.

**What Did the Researchers Do and Find?:**

- We recruited 26 patients for this study and administered afatinib daily, and 10 patients went on to be treated with daily afatinib and weekly vinorelbine, a chemotherapy drug, upon disease progression.
- Thirty-five percent (9 of 26) and 20% (2 of 10) of patients had clinical benefit from being treated with afatinib monotherapy and afatinib plus vinorelbine, respectively.
- We sequenced tumour biopsies before and after afatinib treatment and found that IBC has a higher mutational load and more frequent mutations in the well-known cancer gene *TP53*, compared to non-IBC.
- We did not identify any single gene or mutation that led to afatinib resistance, and biopsies before and after treatment were very similar genetically.

**What Do These Findings Mean?:**

- Afatinib appears to be clinically active in HER2-positive IBC, albeit in a relatively small patient cohort.
- The high mutational load in IBC suggests that checkpoint inhibitors, a type of cancer immunotherapy, might potentially be an effective way of treating patients.

## Introduction

Inflammatory breast cancer (IBC) is a rare, aggressive form of breast cancer that accounts for around 1%–6% of breast cancers [1–4]. IBC tends to affect younger women and has a high risk of local and distant metastasis. Prognosis is poor, with median survival estimated at 2.9 y in IBC patients versus 6.4 y in those with non-inflammatory, locally advanced breast cancer [3,5]. Current management of IBC involves a combination of anthracycline and taxane-based chemotherapy in the neoadjuvant setting, followed by surgery, adjuvant chemotherapy, or radiotherapy [6].

IBC is thought to be a biologically distinct form of breast cancer, commonly lacking oestrogen (ER) and progesterone (PgR) receptor expression [7]. A greater frequency of HER2 and EGFR overexpression among IBC cases has been reported, occurring in 50% and 30% of patients, respectively [8]. Genomic profiling techniques have led to the identification of genes that are potentially involved in disease development [9–11]; however, HER2-positive IBC has not been characterised through deep exome sequencing.

EGFR and HER2 have been shown to be involved in tumour growth and metastasis of IBC, and as such represent therapeutic targets [12]. Afatinib is a small molecule tyrosine kinase inhibitor that irreversibly and selectively blocks signalling from ErbB family members. Clinically, afatinib showed activity in phase II trials with HER2-positive breast cancer patients [13,14]. Most recently, in the phase III LUX-Breast 1 trial, afatinib combined with vinorelbine demonstrated similar progression-free survival (PFS) and objective response (OR) rates to trastuzumab plus vinorelbine in patients with HER2-positive metastatic breast cancer after failure on trastuzumab, but the afatinib-containing regimen was associated with shorter overall survival (OS) and was less tolerable [15].

We performed an open-label, single-arm phase II clinical trial to investigate the efficacy and safety of afatinib alone and in combination with vinorelbine following disease progression in patients with HER2-positive IBC. Recruitment to this trial was terminated early following the results of the LUX-Breast 1 trial. We carried out prospectively planned whole-exome sequencing of tumour biopsies at baseline and after progression on afatinib monotherapy to explore two questions: (1) what is the mutational landscape of HER2-positive IBC, and is it distinct from HER2-positive non-IBC; and (2) how does exposure to HER2 inhibition affect the evolution of IBC?

## Methods

### Study Design

This was an open-label, phase II, multicentre trial of afatinib for the treatment of HER2-positive IBC (ClinicalTrials.gov NCT01325428, S1 and S2 Texts). Patients were treated with afatinib monotherapy until disease progression (Part A), and then afatinib and vinorelbine until disease progression (Part B).

PFS was assessed separately for Part A and Part B, and over the whole study. OS was only assessed over the whole study period. The primary endpoint was clinical benefit (defined as stable disease [SD] for ≥6 mo, partial response [PR], or complete response [CR]). Secondary endpoints were objective response (OR) and duration of OR and PFS; other endpoints included OS and safety.

Following the results of the LUX-Breast 1 trial, Part B was stopped and recruitment to the whole trial was stopped thereafter. Patients in Part A were informed that they would no longer be able to receive afatinib plus vinorelbine upon progression, and had to agree with the investigator regarding continuation of afatinib monotherapy. Patients in Part B who were deriving benefit from treatment could continue afatinib plus vinorelbine.

PR was considered to be confirmed if the criteria were met at least 4 wk later. SD had to be observed at least 42 days after first study drug administration in the respective part of the study to be considered for best overall response regardless of confirmation, and had to last for more than 182 d to qualify for clinical benefit.

The study was conducted in line with the Declaration of Helsinki, the International Conference on Harmonization Good Clinical Practice Guideline and approved by the local ethics committees (S1 Appendix). All patients provided written informed consent prior to study participation.

### Patients

Female patients aged ≥18 y with investigator-confirmed IBC characterized by diffuse erythema and oedema (peau d’orange) with locally advanced or metastatic disease and histologically confirmed HER2-positive disease (i.e. immunohistochemistry [IHC] 3+ or IHC 2+ with FISH/SISH positivity) were eligible for the study (S1 Table). Patients were required to have an Eastern Cooperative Oncology Group (ECOG) status of 0–2 and life expectancy of ≥6 mo. Other exclusion criteria for the trial included: radiotherapy, chemotherapy, hormone therapy, immunotherapy, trastuzumab, or surgery (other than biopsy) within 2 wk prior to the first dose of afatinib in Part A, known pre-existing interstitial lung disease, active brain metastases, significant chronic or recent acute gastrointestinal disorders with diarrhoea as a major symptom, any other current malignancy or malignancy diagnosed or relapsed within the past 5 y (other than non-melanomatous skin cancer and in situ cervical cancer), inadequate bone marrow, and renal and liver functions.

### Treatments

In both parts of the study, patients received a single oral dose of afatinib 40 mg once daily until disease progression. The first dose was administered at the trial site, and subsequent doses were taken at home. Afatinib dose reductions were required for any drug-related grade ≥3 adverse events (AEs) and selected grade 2 AEs. The afatinib dose was reduced in 10 mg decrements to a minimum of 20 mg; all dose reductions were permanent. In Part B, patients received previously tolerated afatinib doses and additionally received short infusion (approximately 10 min) intravenous vinorelbine at a weekly dose of 25 mg/m^2^ in a 4-weekly course until disease progression. Vinorelbine treatment was administered at the trial site under the supervision of the investigator; treatment was withheld if platelet count was <100,000 cells/mm^3^ or absolute neutrophil count was <1,500 cells/mm^3^.

### Assessments

Tumour assessments were performed by computed tomography or magnetic resonance imaging at screening and every 8 wk after the first dose of afatinib. Investigators evaluated response according to Response Evaluation Criteria in Solid Tumors (RECIST) version 1.1. AEs were graded using Common Terminology Criteria for Adverse Events (CTCAE) version 3.0.

### Whole-Exome Sequencing

Tumour biopsies were obtained before afatinib treatment and on disease progression in Part A, snap frozen, and optimal cutting temperature compound (OCT)-embedded. Venous blood samples were obtained and genomic DNA was extracted. Whole-exome sequencing was performed on pre-treatment tumour biopsies, matched germline genomic DNA and post-treatment tumour biopsies according to the manufacturer’s protocol (Agilent SureSelect Human All Exon 50Mb Kit). Tumour and germline DNA were sequenced at the Beijing Genomics Institute on the Illumina HiSeq 2000 to an average depth of 396x and 157x, respectively (S2 Table).

### Mutation Calling and Genomic Analysis

Raw sequencing data were aligned to human genome sequence version hg19 using bwa (v0.5.9) [16], duplicates marked using Picard (v1.54), and indel realignment performed with GATK IndelRealigner (v1.0.6076) [17]. Somatic single nucleotide variant (SNV) calling was performed using VarScan2 (v2.3.7) [18], MuTect (v1.1.7) [19], Virmid (v1.1.0) [20], and Strelka (v1.0.14) [21]. SNVs called by ≥2 tools were further filtered for variant allele frequency (VAF) ≥5%. Small indels were identified using Pindel (v0.2.5a7) [22] and VarScan2 (v2.3.7). Indels called by both tools were further filtered for VAF ≥5%. Mutations in genes of interest were visualized with Oncoprints [23].

Tumour copy number aberrations, ploidy, and purity were determined using ASCAT 2 [24], which allows for exome sequencing data as input (available at https://github.com/Crick-CancerGenomics/ascat) (S3 Table). Some samples were excluded from copy number analysis due to low tumour content. Segmented copy number data were divided by sample mean ploidy and log2 transformed for GISTIC2.0 analysis [25]. Copy number segments were defined relative to ploidy as previously described [26]: amplification, gain, and loss were defined as log_2_(4/2), log_2_(2.5/2), and log_2_(1.5/2), respectively.

Genome-doubling status was determined as previously described [27]. Briefly, each sample, s, was represented as an aberration profile of major and minor allele copy numbers at chromosome arm resolution. The total number of aberrations (relative to diploid), Ns, and the probabilities of loss/gain for each allele at each chromosome arm, Ps, was calculated. Ten thousand simulations were run for each sample s, where Ns sequential aberrations, based on Ps, were applied to a diploid profile. A *p*-value for genome doubling was obtained by counting the percentage of simulations in which the proportion of chromosome arms with a major allele copy number ≥2 was higher than that observed in the sample.

The weighted Genomic Instability Index (wGII) was used to assess chromosomal instability [28]. Briefly, the percentage aberrant regions for each autosome was calculated separately and mean percentage aberration then calculated across all 22 chromosomes to account for variation in chromosome size, so that large chromosomes do not have a greater effect on the GII score than small chromosomes.

Mutational signatures were determined using the R package deconstructSigs [29]. Using this tool, the fraction of mutations in each of the 96 trinucleotide contexts was calculated, and the weighted combination of published signatures from [30] identified to most closely reconstruct the mutational profile of the sample.

The mutation copy number and cancer cell fraction of each mutation were calculated by integrating ASCAT-derived integer copy number and tumour purity estimates with the variant frequency as described in [31]. This was used as input for PyClone [32], which uses a hierarchical Bayesian Dirichlet process in order to infer clonal population structure. A modified version of PyClone was used as described in [33]; clusters with 3 or fewer SNVs were excluded.

HLA typing was performed with OptiType [34]. Nonsynonymous mutations were extracted from each tumour sample and translated into mutant peptide 9–11mers long [33]. Using the patient-specific HLA type, we used NetMHC (v2.8) [35] to predict the binding strength of each mutant and wildtype peptide to the respective MHC class I molecules. Somatic mutations that gave rise to peptides with a binding affinity of ≤500 nM were considered to be putatively neoantigenic.

### TCGA Data

The comparison to the HER2-positive non-IBC cohort is based upon data generated by The Cancer Genome Atlas (TCGA) Research Network: http://cancergenome.nih.gov/ [36]. Tumour samples were filtered for positive HER2 IHC status (*n* = 131, S4 Table). For the matched cohort, a subset was selected by matching cases based on age (±10 years), ER, and PgR status; this allowed only for a one-to-one matching due to the limited number of TCGA cases available.

### Statistical Analysis

Analyses of efficacy and safety in this trial were descriptive and exploratory. A sample size of 40 patients was selected for this study; assuming an underlying clinical benefit rate of 50%, 40 patients would provide more than a 90% probability of observing a clinical benefit rate of at least 40%. Analyses of clinical benefit rate (CBR) and OR rate were planned for the following subgroups: hormone receptor (ER and PgR), EGFR status, new brain metastases, patients presenting with target lesions only versus those with non-target lesions only versus those with both, and prior trastuzumab therapy. Exploratory analyses using genomic data were planned to search for predictive markers of response and resistance to afatinib.

MutSigCV (v1.3) [37] and GISTIC2.0 [25] were used to determine mutational significance of somatic SNVs and somatic copy number alterations (SCNAs). Multiple-testing corrections in these tests were carried out using the Benjamini-Hochberg false discovery rate method. Mann-Whitney and Fisher’s exact test were used for comparison between two groups. Survival curves were estimated using the Kaplan-Meier method, and the log-rank test was used to test for significance.

## Results

### Patients

**Fig 1:**
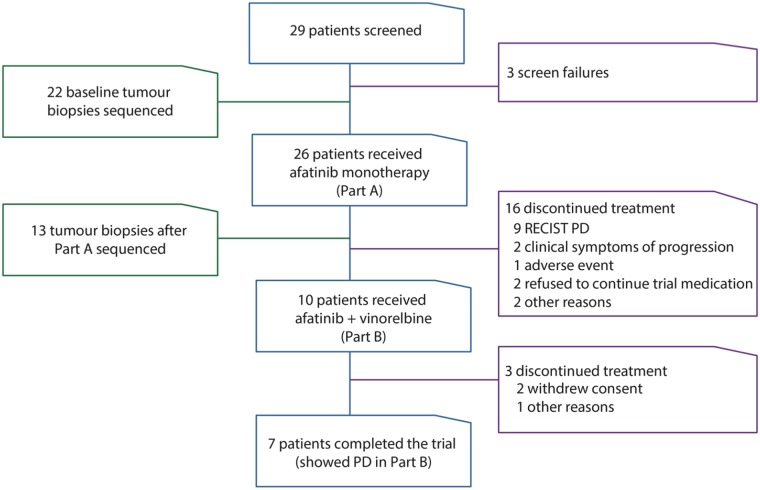
Study flow and patient disposition. This figure describes the study and number of patients in each part of the clinical trial (blue outline), reasons for patients being excluded or discontinuing treatment (purple outline), and number of patients with genomic analysis performed (green outline). PD, progressive disease.

The study was performed at 14 centres in seven countries between December 2011 and November 2014. Twenty-nine patients were screened, and 26 received afatinib monotherapy; of these, 10 patients continued into Part B of the study (Fig 1). Twenty-four of 26 patients had metastatic disease at study inclusion. Patient demographics at baseline are shown in Table 1.

**Table 1:** Patient demographics at beginning of study.

Characteristic	Part A:	Part B:
	Afatinib monotherapy	Afatinib plus vinorelbine
	(*n* = 26)	(*n* = 10)
**Sex, *n* (%)**		
Female	26 (100)	10 (100)
**Age, years**		
Mean (SD)	51.5 (8.8)	51.5 (12.5)
**Race, *n* (%)**		
Asian	17 (65)	7 (70)
- Southeast Asian	13 (50)	5 (50)
- Korean	4 (15)	2 (20)
Black/African American	1 (4)	0
White	8 (31)	3 (30)
**Smoking status, *n* (%)**		
Never smoker	22 (85)	7 (70)
Ex-smoker	4 (15)	3 (30)
**Body mass index, kg/m** ^**2**^		
Mean (SD)	25.0 (3.9)	26.0 (4.5)
**Tumour histology, *n* (%)** *		
Infiltrating duct carcinoma	23 (88)	10 (100)
Papillary carcinoma	1 (4)	0
Infiltrating lobular carcinoma	2 (8)	0
Paget disease	1 (4)	1 (10)
Other	2 (8)	1 (10)
**ER status at first diagnosis, *n* (%)**		
Positive	13 (50)	4 (40)
Negative	13 (50)	6 (60)
**PgR status at first diagnosis, *n* (%)**		
Positive	6 (23)	1 (10)
Negative	20 (77)	9 (90)
**HER2 status at first diagnosis, *n* (%)**		
Positive	26 (100)	10 (100)
Negative	0	0
**Metastatic sites at study inclusion, *n* (%)**		
0	2 (8)	0
1	5 (19)	1 (10)
2	6 (23)	2 (20)
3	9 (35)	4 (40)
≥4	4 (15)	3 (30)
**Prior chemotherapy, *n* (%)**		
Yes	18 (69)	7 (70)
No	8 (31)	3 (30)
**Prior trastuzumab, *n* (%)**		
Yes	7 (27)	4 (40)
No	19 (73)	6 (60)

*Patients could have more than one type of tumour histology.

### Efficacy with Afatinib (Part A)

Nine (35%) of 26 treated patients had confirmed clinical benefit with afatinib monotherapy (eight PRs and one SD of ≥6 months; Table 2, S5 Table). Three patients had an unconfirmed PR, resulting in an overall response rate (ORR) of 42% (*n* = 11). Twenty (77%) patients progressed or died on afatinib monotherapy; median PFS was 110.5 days (95% CI 58.0–386.0). In total, there were three on-treatment and one post-study deaths. Planned subgroup analyses were performed (S1 Fig). Clinical benefit with afatinib monotherapy was achieved in 0 of 7 trastuzumab-treated patients and 9 of 19 (47%) trastuzumab-naïve patients. Median PFS with afatinib monotherapy was apparently shorter in trastuzumab-treated patients versus trastuzumab-naïve patients (64 versus 151 days, *p*-value = 0.099, log-rank test; S2 Fig).

**Table 2:** Summary of efficacy in patients enrolled in clinical trial.

Measurement	Part A:	Part B:
	Afatinib monotherapy	Afatinib plus vinorelbine
	(*n* = 26)	(*n* = 10)
**Clinical benefit, *n* (%)**	9 (35)	2 (20)
Confirmed PR	8 (31)	1 (10)
SD ≥6 mo	1 (4)	1 (10)
**SD <6 mo**	7 (27)	4 (40)
Unconfirmed PR	3 (12)	2 (20)
**Progressive disease, *n* (%)**	8 (31)	3 (30)
**Not evaluable**	2 (8)	1 (10)
**Progression-free survival, days** Median (95% CI)	110.5 (58.0–386.0)	106.0 (36.0–190.0)
**Overall survival, days** Median (95% CI)	713.0 (400.0–NE)	

Results in this table according to RECIST (v1.1), based on best overall response. Clinical benefit: confirmed CR, PR, or SD ≥6 mo; NE, not estimable.

### Efficacy with Afatinib plus Vinorelbine (Part B)

Following progression on afatinib monotherapy, ten patients received afatinib plus vinorelbine (Part B). Confirmed clinical benefit was achieved in two (20%; Table 2, S5 Table, S1 Fig); a further two patients had an unconfirmed PR, with an overall CBR rate of 40% (*n* = 4). Eight (80%) patients progressed or died. Median duration of PFS was 106.0 d (95% CI 36.0–190.0).

OS was analysed across the whole study. Eleven (42%) patients died during the study, and median OS was 713.0 d. Median PFS across the whole study was shorter in trastuzumab-treated patients versus trastuzumab-naïve (136 versus 395 d, *p*-value = 0.024, log-rank test, S3 Fig).

### Safety

Median duration of exposure to treatment was 111 d (range: 17–700) in Part A and 84.5 d (range: 42–237) in Part B. All patients had treatment-related AEs (Table 3).

**Table 3:** Most common treatment-related adverse events reported in ≥10% of patients.

Adverse Event	Part A:		Part B:	
	Afatinib monotherapy		Afatinib plus vinorelbine	
	(*n* = 26)		(*n* = 10)	
	All-grade	Grade ≥3	All-grade	Grade ≥3
Any	26 (100)	10 (38)	10 (100)	7 (70)
Diarrhoea	24 (92)	6 (23)	7 (70)	2 (20)
Rash	17 (65)	0	1 (10)	0
Decreased appetite	10 (38)	1 (4)	2 (20)	0
Mucosal inflammation	9 (35)	3 (12)	3 (30)	1 (10)
Nausea	7 (27)	0	5 (50)	0
Paronychia	7 (27)	1 (4)	0	0
Vomiting	6 (23)	0	1 (10)	0
Weight decreased	6 (23)	1 (4)	3 (30)	0
Dermatitis acneiform	5 (19)	1 (4)	0	0
Epistaxis	4 (15)	0	0	0
Fatigue	4 (15)	1 (4)	3 (30)	1 (10)
Dry eye	3 (12)	0	0	0
Erythema	3 (12)	0	0	0
Palmar-plantar erythrodysaesthesia	3 (12)	0	0	0
Stomatitis	3 (12)	0	1 (10)	1 (10)
Neutropenia	1 (4)	0	8 (80)	7 (70)
Anaemia	0	0	5 (50)	2 (20)
Abdominal pain*	2 (8)	0	3 (30)	0
Dyspnoea	0	0	2 (20)	0

*Reported as abdominal pain upper in Part A.

### Mutational Landscape of HER2-Positive IBC

**Fig 2:**
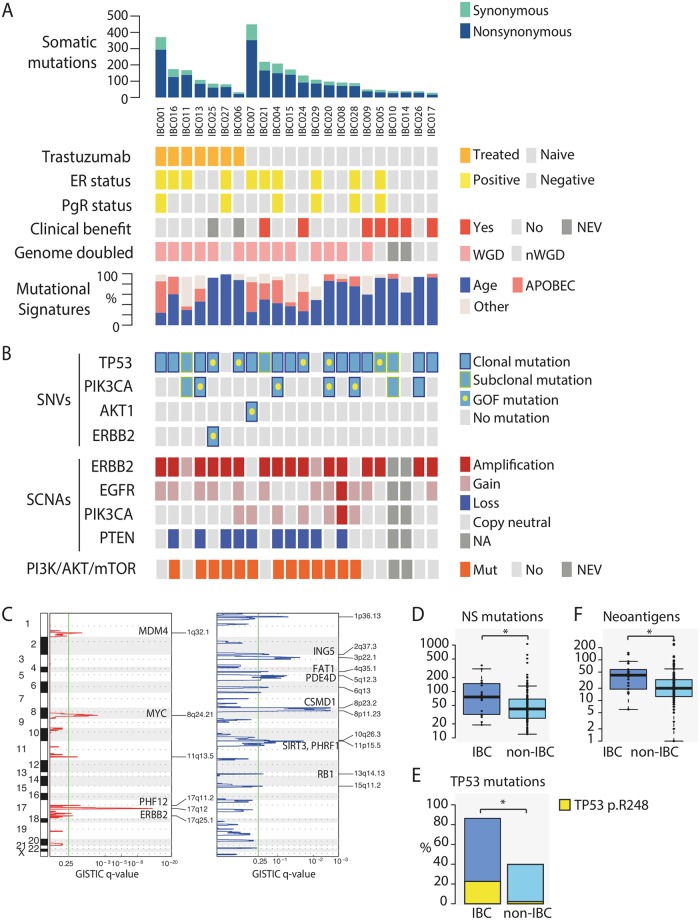
Somatic mutations in HER2-positive IBC. (A) Top panel shows number of somatic mutations (SNVs and indels) identified across the 22 IBC patients. Data tracks below indicate if patient was: treated with trastuzumab prior to afatinib monotherapy (orange); oestrogen-receptor (ER) or progesterone-receptor (PgR) positive (yellow); derived confirmed clinical benefit from afatinib monotherapy (red); tumour underwent whole-genome doubling (WGD) (pink). Mutational signatures identified in IBC tumours were predominantly age-related (Signatures 1A and 1B) (blue), APOBEC-related (Signatures 2 and 13) (salmon), and others (grey). NEV, not evaluated; NA, no information available. (B) *TP53*, *PIK3CA*, *AKT1*, and *ERBB2* mutations identified in samples are indicated if present (blue) or absent (grey). Gain-of-function mutations (*TP53* p.R248, *PIK3CA* p.H1047R, *AKT1* p.E17K, *ERBB2* p.V777L) are indicated by a yellow dot. Clonal and subclonal mutations are indicated by dark blue and yellow outlines, respectively. Amplifications (≥2x ploidy), gains (≥1 copy number relative to ploidy), and losses (≤1 copy number relative to ploidy) in *ERBB2* (*HER2*), *PIK3CA*, *EGFR*, and *PTEN* are indicated by red, pink, and dark blue, respectively. Somatic activation of PI3K/AKT/mTOR pathway (defined as *PIK3CA* activating mutation or gain, *PTEN* deletion, *AKT1* mutation) indicated in orange. (C) Plots showing results of GISTIC analysis identifying recurrent focal gains (left panel in red) and losses (right panel in blue); *y*-axis is genomic position and *x*-axis is GISTIC q-value; green line represents significance threshold (q-value = 0.25). Gene names are indicated where significantly mutated cancer driver genes were previously associated with the GISTIC peak in a pan-cancer analysis of SCNAs [38]. (D) Box plot showing higher numbers of somatic nonsynonymous (NS) mutations identified in IBC patients compared to non-IBC patients. The band inside the box denotes median. (E) Bar plot showing an enrichment of *TP53* mutations in IBC patients versus non-IBC patients. Yellow bar is proportion of gain-of-function *TP53* p.R248 mutations. (F) Boxplot showing higher numbers of neoantigens predicted in IBC patients compared to non-IBC patients. Asterisk (*) denotes significant *p*-value <0.05.

Twenty-two of 26 patients in Part A had tumour biopsy material suitable for whole-exome sequencing analysis (Fig 2). Overall, we identified an average of 134.5 (range: 30–468) somatic coding mutations (Fig 2A, S6 Table). The most commonly mutated gene was *TP53* (MutSig q-value = 1.68x10^-11^); 86.4% (19/22) of the tumours harboured a somatic mutation in *TP53* (S7 Table). Strikingly, five patients had gain-of-function *TP53* mutations at hot-spot residue p.R248 (S4 Fig). Planned exploratory analyses showed that OS was non-significantly shorter in patients carrying *TP53* p.R248 mutations pre-treatment versus those with loss-of-function (nonsense, frame-shift, splice site) mutations (398 versus 652 d, *p*-value = 0.626, log-rank test). Patients IBC007 and IBC001 had much higher numbers of somatic SNVs compared to the rest of the cohort (468 and 393, respectively) but did not have mutations in known DNA mismatch repair genes.

Mutations in the PI3K/AKT/mTOR pathway are frequent in breast cancer, and activation of this pathway via molecular aberrations in *PIK3CA*, *PIK3CB*, *PIK3R1*, *AKT*, *TSC1/2*, and *PTEN* promotes resistance to HER2-targeted therapies [39–41]. Seven patients harboured *PIK3CA* mutations, including four with hotspot mutation p.H1047R [42]. IBC007 harboured an activating *AKT1* p.E17K mutation, and IBC025 carried an activating *ERBB2* p.V777L mutation (Fig 2B) [43,44]. No other somatic mutations in this pathway were identified.

In order to gain insight into the mutational processes shaping the IBC landscape, we utilized previously extracted mutational signatures and applied them to the IBC cohort (Fig 2A, S5A Fig) [29,30]. Signature 1A, which was previously associated with age of diagnosis [45], accounted for the majority of mutations (64.2% ± 26.1). Signatures 2 and 13, attributed to activity of the APOBEC family of cytidine deaminases, were together present in 64% (14/22) of tumours (16.4% ± 19.7% of somatic mutations). In particular, the excess of mutations in IBC001 and IBC007 appear to be driven by APOBEC mutagenesis (S5B Fig), which was observed in both clonal and subclonal mutations for these samples (S5C Fig).

SCNA calling was possible in 20 of 22 tumours (S8 Table). Seventy percent (14/20) of the tumours had undergone whole-genome doubling and had higher genomic instability scores (wGII) compared to non-genome-doubled tumours (0.54 ± 0.18 versus 0.31 ± 0.06, *p*-value = 4.6x10^-3^, Mann-Whitney) (Fig 2A). Even though all IBC patients were HER2-positive via IHC or FISH (S1 Table), only 16 of 20 tumours were called as having *ERBB2* amplification; two tumours (IBC011 and IBC029) harboured gains and two tumours (IBC007 and IBC028) had neither amplification nor gain of *ERBB2* (Fig 2B). It is possible that these four tumours could represent false negatives due to reasons such as sampling bias caused by intra-tumour heterogeneity or normal tissue contamination. Sixty percent (12/20) of tumours had *EGFR* gains (11 gains, 1 amplification), consistent with previous reports [8]; 10 tumours had *PTEN* loss (Fig 2B). GISTIC [25] analysis revealed recurrent focal amplifications across 6 loci, including 17q12 (q-value = 9.22x10^-13^), 8q24.21 (q-value = 4.89x10^-3^), and 1q32.1 (q-value = 5.80x10^-2^) containing *ERBB2*, *MYC*, and *MDM4*, respectively (Fig 2C, S9 Table). Recurrent focal losses were identified across 12 chromosomal regions, including 11p5.15 (q-value = 2.09x10^-2^) containing *SIRT3* and *PHRF1*.

We carried out planned exploratory analyses to identify predictive markers of response and resistance to afatinib. We did not identify an association between *EGFR* gains or *HER2* amplifications and response to afatinib. Since activation of PI3K/Akt signalling is thought to impact the efficacy of HER2-targeted treatment [46–48], we focused on mutations in this pathway to explore any potential impact on PFS. We observed that somatic activation of this pathway (i.e. *PIK3CA* activating mutation or gain, *ERBB2* activating mutation, *PTEN* deletion, *AKT1* activating mutation) was significantly associated with shorter PFS in trastuzumab-naïve patients (*p*-value = 0.03, S6 Fig). Although activating mutations of the PI3K pathway have been reported as occurring more frequently in ER-positive breast tumours [40], we did not observe a difference in this small cohort (6/10 ER-positive versus 7/12 ER-negative). Unexpectedly, a trastuzumab-naïve patient (IBC024) harbouring a gain overlapping *PIK3CA* and *PTEN* heterozygous deletion at baseline showed a PR for 48 wk before disease progression.

### Genomic Analysis of HER2-Positive IBC versus HER2-Positive non-IBC

To determine if there were significant differences in mutational profiles between HER2-positive IBC and HER2-positive non-IBC, we compared our results against a cohort of TCGA patients with HER2-positive breast cancer (*n* = 131, S4 Table) [36]. We observed that the average number of somatic protein-changing mutations per patient was higher in IBC than non-IBC patients (102.4 ± 89.4 versus 71.9 ± 115.3; *p*-value = 0.0107, Mann-Whitney) (Fig 2D).

Given that *TP53* was the only significantly mutated gene identified in IBC, we compared the mutation burden of this gene between the 2 cohorts. We observed that *TP53* mutations were significantly enriched in the IBC cohort compared to non-IBC (19/22 versus 53/131; *p*-value = 5.76x10^-5^, Fisher’s exact) [49], as were *TP53* hotspot p.R248 mutations (5/19 versus 3/53; *p*-value = 0.026, Fisher’s exact) (Fig 2E). Consistent with the higher mutational load, IBC tumours also had a higher number of predicted neoantigens compared to non-IBC (49.59 ± 37.9 versus 31.0 ± 41.8, *p*-value = 8.39x10^-4^, Mann-Whitney) (Fig 2F). Similar to IBC, the most prevalent mutational processes among the non-IBC cohort were age and APOBEC-related, with similar distributions of these mutational signatures between the 2 cohorts (S5D Fig).

There were no significant differences in the proportion of genome-doubled tumours (14/20 versus 75/131, *p*-value = 0.34, Fisher’s exact) or wGII scores (0.47 versus 0.51, *p*-value = 0.3843, Mann-Whitney) between IBC and non-IBC tumours. Applying GISTIC to the non-IBC tumours, 5 of 6 recurrently amplified regions and all 12 recurrently deleted regions in IBC had wide-peak boundaries that overlapped with those of non-IBC tumours (S9 Table). Only the 11q13.5 amplification in IBC did not overlap with non-IBC, which includes *PAK1*, an oncogene that activates MAPK and MET signalling and regulates cell motility; interestingly, previous reports have associated IBC with MAPK hyperactivation [50,51].

Utilizing an age and ER/PgR status matched cohort (Methods), the results were concordant, with a higher burden of somatic protein-changing mutations, neoantigens, and *TP53* mutations in IBC versus non-IBC (S7 Fig).

### Tracking Genomic Evolution of Afatinib Treated IBC

**Fig 3:**
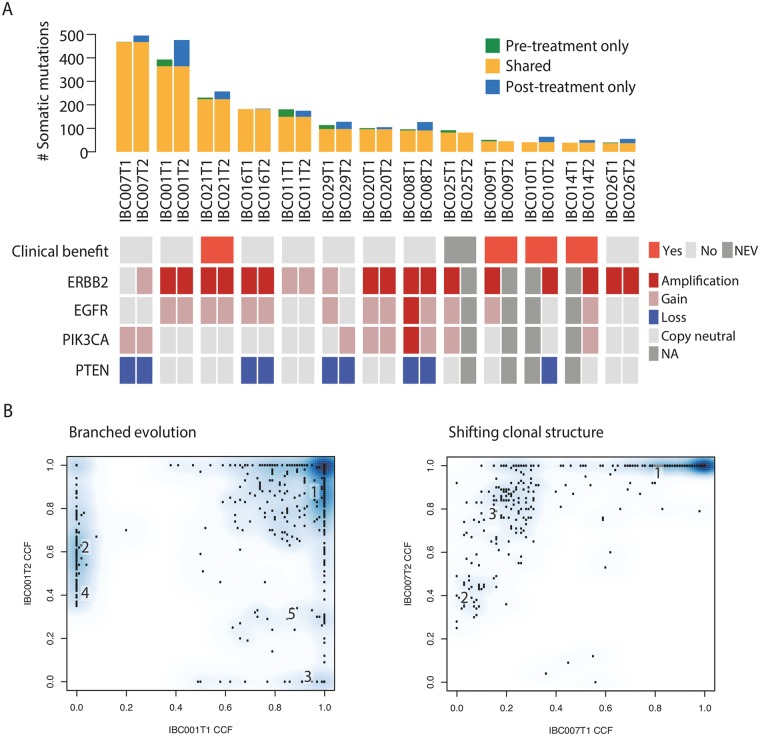
Genomic analysis of tumour biopsies before treatment and following disease progression on afatinib monotherapy. (A) Somatic mutations (SNVs and indels) identified in pre- and post-treatment biopsies. Green, mutations identified in pre-treatment only; yellow, mutations identified in both pre- and post-treatment; blue, mutations identified in post-treatment only. Data tracks below denote: if patient derived confirmed clinical benefit from afatinib monotherapy (red); amplifications (≥2x ploidy), gains (≥1 copy number relative to ploidy), and losses (≤1 copy number relative to ploidy) in *ERBB2* (*HER2*), *EGFR*, *PIK3CA*, and *PTEN* are indicated by red, pink, and blue, respectively. NEV, not evaluated; NA, no information available. (B) Two main patterns of clonal evolution following afatinib monotherapy observed, either branched evolution or shifting clonal structure. Numbers refer to mutation clusters from PyClone results, also in S10 Fig. T1, pre-treatment biopsy; T2, post-treatment biopsy; CCF, cancer cell fraction.

Among 13 tumour biopsies obtained following disease progression, we identified an average of 181.4 (range: 50–505) somatic mutations, of which 79.1% ± 12.0% were shared with baseline tumours (Fig 3A, S10 Table). The overall mutation burden in tumours following treatment was higher in post-treatment samples compared to pre-treatment samples (172.5 ± 136.7 versus 156.1 ± 151.9, *p*-value = 0.030, paired *t* test). No recurrent mutations were identified among newly arising mutations post-treatment, and no new mutations in PI3K/Akt pathway genes were identified, aside from a *MTOR* p.K30N mutation (variant of unknown significance) in IBC021.

Nine of 13 matched pairs had copy number data (S11 Table); all tumours had the same genome-doubling status pre- and post-treatment, and there was no difference in ploidy (2.9 versus 2.9, *p*-value = 0.80) or wGII scores (0.42 versus 0.46, *p*-value = 0.55) (S8 Fig, S3 Table) between pre- and post-treatment samples. *ERBB2* amplification status appeared to change in two of nine patients, from gain to copy-neutral in IBC029 and from copy-neutral to gain in IBC007 (Fig 3A). Overall, SCNAs between paired samples (*n* = 9) were highly concordant, and unsupervised hierarchical clustering showed that tumour biopsies clustered by patient rather than treatment stage (S9 Fig).

Drug resistance may arise as a consequence of an evolutionary bottleneck, where a resistant subclone is selectively enriched during therapy [52]. We utilized previously described methods to compare the clonal architecture of tumours before and after treatment [32,53]. Of these nine patients, IBC021 was the only patient with confirmed clinical benefit. We observed in all patients a cluster of variants that was clonal in both pre- and post-treatment biopsies (cancer cell fraction [CCF] around 1.0 on both *x* and *y* axis in S10 Fig); all gain-of-function *PIK3CA* and *TP53* mutations, when present in the tumour, belonged to this cluster. In eight of nine patients, we observed some evidence of branching evolution, with new clones identifiable in the post-treatment samples and others declining in frequency or disappearing (Fig 3B, S11 Fig). Interestingly, the majority of mutations identified after treatment were detected in the pre-treatment tumour biopsy at a similar CCF (S10 and S11 Figs), and the overall clonal composition in all 8 tumours remained largely similar between the two time points with little evidence of bottlenecking, consistent with the lack of confirmed benefit in these patients, aside from IBC021. In one patient (IBC007), new clones were not observed, but there were distinct clonal shifts; there was clonal expansion of two subclones from 2% to 38% and 22% to 81%, and the major clone decreased slightly from 96% to 78%; no known drivers were identified in the subclones (S10 Table). Importantly, we cannot exclude the possibility that the observed dynamics could be due to tumour sampling bias between pre- and post-treatment samples.

## Discussion

Longitudinal analysis of the genomic evolution of tumours during therapy can inform drug resistance mechanisms and the changing landscape of disease over time. Here, we report the first prospectively planned clinical trial in IBC with genomic analysis, and the first assessment of afatinib with or without vinorelbine in patients with HER2-positive IBC.

Afatinib monotherapy demonstrated activity in patients with HER2-positive IBC, with nine (35%) patients achieving clinical benefit and median PFS of 110.5 d. This is concordant with data from a phase II trial assessing lapatinib 1500 mg daily in 126 patients with relapsed or refractory HER2-positive IBC, in which no patients had a CR but 49 (39%) had a PR and median PFS was 102.2 d [54]. Following progression on afatinib monotherapy, two (20%) patients achieved clinical benefit with addition of vinorelbine, and median PFS in Part B was 106.0 d.

The most common treatment-related events reported during the trial were diarrhoea, rash, and decreased appetite in Part A, and neutropenia, diarrhoea, nausea, and anaemia in Part B. Overall, the safety profile observed was generally consistent with previously published data on afatinib and vinorelbine. Importantly, this trial included pre-planned exome analysis of tumour biopsies at two time-points: before treatment and at disease progression. To our knowledge, this is the first report characterising IBC through exome sequencing. We identified a high incidence of *TP53* mutations, as reported previously [49], and an enrichment of p.R248 hotspot DNA-contact mutations that promote nuclear accumulation of p53 [55–57]; cellular and animal studies indicate that these gain-of-function mutations induce increased invasion, chemoresistance and decreased survival [58–60]. Our results showed a non-significant reduction in OS in IBC patients carrying *TP53* p.R248 mutations, consistent with previous analysis [61] and reports of nuclear p53 overexpression representing an adverse prognostic marker in IBC [62–64].

We identified recurrent focal gains across 6 loci and losses across 12 regions, including 11p5.15 containing *SIRT3* and *PHRF1* (also identified in the non-IBC cohort). *SIRT3* is deleted in 40% of human breast tumours, and loss of *SIRT3* increases reactive oxygen species production and HIF-1a stabilization [65]. *PHRF1* functions as a tumour suppressor by promoting the TGF-beta cytostatic programme [66]; a recent transcriptomic study identified reduced TGF-beta signalling as a specific gene expression signature of IBC compared to non-IBC [67]. Comparing IBC to non-IBC, the only different recurrent focal SCNA was the amplification of 11q13.5 containing *PAK1* in IBC; *PAK1* is an oncogene that activates MAPK and MET signalling and regulates cell motility, and previous reports have associated IBC with MAPK hyperactivation [50,51].

We compared tumours before and after afatinib monotherapy to investigate potential drivers of resistance. The tumour pairs displayed a high degree of genetic relatedness, both in terms of point mutations and large-scale genomic aberrations. We did not observe changes in *ERBB2* amplification status in the majority (7/9 or 78%) of our tumours, consistent with previous reports of loss of HER2-positivity occurring in only 12%–32% of patients undergoing anti-HER2 therapy [68–71]. In the two patients who appeared to undergo a change in amplification status, we are unable to conclude if the lack of *ERBB2* amplification (in the pre-treatment biopsy for IBC007 and in the post-treatment biopsy for IBC029) was due to technical limitations of exome sequencing, sampling bias, or selection of a HER2-negative subclone during therapy (in the case of IBC029).

In contrast to *EGFR* mutant lung adenocarcinomas, in which the T790M gatekeeper mutation is commonly selected following EGFR inhibitor exposure [72], there was no evidence of selection for mutations in specific genes in the post-treatment IBC tumours. Eight out of 9 tumour pairs displayed branching evolution, with new clones emerging and others disappearing after treatment, possibly reflecting the differential effect that afatinib monotherapy had on the different subclones; it is worth noting that only 1 of 8 of these patients (IBC021) derived confirmed clinical benefit from afatinib monotherapy. It is also possible that subclones detected only in the pre- or post-treatment tumour biopsy in this study could be related to sampling bias or caused by the “illusion of clonality” derived from a single-region biopsy. Regardless, the majority of mutations in these tumours were shared between the two time points and possessed largely similar clonal compositions, concordant with previous reports in pre- and post-treatment samples of multiple myeloma and high-grade serous ovarian carcinoma [53,73]. IBC007 was the only tumour with an apparent shift in clonal structure, possibly reflecting random drift of tumour clones or sampling bias, given that this patient did not respond to afatinib monotherapy [32,53]. Immune checkpoint inhibitors have been shown to provide clinical benefit in a variety of cancers, including melanoma and lung cancer [74–77]. In particular, a high mutational load (>100 somatic nonsynonymous coding mutations) was reported as significantly correlated with improved OS in patients with metastatic melanoma treated with ipilimumab or tremelimumab [78]. Several clinical trials investigating the efficacy of checkpoint inhibitors have already been initiated in HER2-positive breast cancer (NCT02734004, NCT02605915, NCT02318901, NCT02403271) and HER2-positive gastric cancer (NCT02689284). The mutational burden in our study revealed an average of 102.4 nonsynonymous mutations in baseline HER2-positive IBC, above the threshold indicated for clinical benefit with anti-CTLA4 therapy [78]. The high mutational and neoantigenic load associated with HER2-positive IBC suggests a potential role for checkpoint inhibitor therapy in this disease.

Following the results of the LUX-Breast 1 trial, recruitment to this study was terminated early. As such, a limitation of this study is the relatively small sample size of HER2-positive IBC patients, making it difficult to draw robust conclusions regarding clinical efficacy of afatinib in this disease. Furthermore, single-region biopsies could be leading to underestimation of tumoural heterogeneity and clonal dynamics.

In conclusion, this phase II trial demonstrated that afatinib, with or without vinorelbine, showed activity in patients with HER2-positive IBC in trastuzumab-naïve patients, albeit in a small patient cohort. This is one of the first clinical trials to fully and prospectively integrate longitudinal exome sequencing with drug development. HER2-positive IBC is characterised by a higher mutational and neoantigenic burden and greater incidence of *TP53* mutations compared to HER2-positive non-IBC. PI3K pathway activation was associated with poorer outcomes on afatinib therapy. Analysis of pre- and post-afatinib monotherapy tumour biopsies did not identify major dynamics of tumour sublcones or recurrent somatic mutations driving resistance. Epigenetic and tumour microenvironmental changes [79,80] may contribute to drug resistance in IBC and should be investigated further in future trials.

This study provides a proof of principle that prospective planning of genomic analysis in clinical trials is feasible in advanced breast cancer, and provides insight into the dynamics of cancer genome evolution through therapy.

## Supporting Information

S1 Appendix Supporting documents for ethical approval. (PDF)

S1 Fig Forest plots of confirmed clinical benefit by subgroups. (A) Subgroup analyses for Part A. (B) Subgroup analyses for Part B.
(TIF)

S2 Fig PFS curves (Part A) of trastuzumab-treated (n = 7) versus trastuzumab-naïve patients (n = 19). *Y*-axis is percentage PFS, *x*-axis is time to PD or death (days). Blue line, trastuzumab-naïve patients; red line, trastuzumab-treated patients.
(TIF)

S3 Fig PFS curves (whole study) of trastuzumab-treated (n = 7) versus trastuzumab-naïve patients (n = 19). *Y*-axis is percentage PFS, *x*-axis is time to PD or death (days). Blue line, trastuzumab-naïve patients; red line, trastuzumab-treated patients.
(TIF)

S4 Fig *TP53* mutations identified in IBC. Each missense, nonsense and frameshift mutation is depicted as a green circle; splice site mutations are not shown. Recurrent gain-of-function p.R248 mutations are labelled. TAD, transcription-activation domain.
(TIF)

S5 Fig Mutational processes underlying somatic mutations in pre-treatment biopsies. (A) Breakdown of mutations driven by age, APOBEC and other mutational processes by patient. (B) Boxplots of number of mutations explained by age, APOBEC and other mutational signatures. (C) Breakdown of mutational signatures in IBC001 and IBC007. (D) Boxplot of different contributions of age, APOBEC-related and other mutational signatures in IBC versus non-IBC tumours.
(TIF)

S6 Fig PFS curves of patients harbouring activating mutations in PI3K/Akt pathway (n = 8) versus those without (n = 5). Activation of PI3K/Akt pathway defined as *PI3KCA* amplification or activating mutation, *PTEN* loss and/or activating mutations in *AKT1* and *ERBB2*. *Y*-axis is percentage PFS, *x*-axis is time to PD or death (days). Blue line, patients without mutations in PI3K/Akt pathway; red line, patients with somatic activation in PI3K/Akt pathway.
(TIF)

S7 Fig Comparing IBC to an age-matched, ER and PgR status matched non-IBC cohort (n = 22). (A) Boxplot showing higher numbers of somatic nonsynonymous (NS) mutations identified in IBC patients compared to non-IBC patients. (B) Barplot showing an enrichment of *TP53* mutations in IBC patients versus non-IBC patients. (C) Boxplot showing higher numbers of neoantigens predicted in IBC patients compared to non-IBC patients.
(TIF)

S8 Fig Ploidy and wGII comparisons between pre- and post-treatment tumours. (A) Ploidy scores in pre-treatment (*x*-axis) and post-treatment (*y*-axis) tumours. Line represents linear regression fit. (B) wGII scores in pre- and post-treatment tumours.
(TIF)

S9 Fig Unsupervised hierarchical clustering of SCNAs in pre- and post-treatment tumour biopsies (n = 9). Pre-treatment biopsies are labelled T1 and in green; post-treatment biopsies are labelled T2 and in blue.
(TIF)

S10 Fig SNV clusters identified in matched tumours pre- and post-treatment. Each of the nine tumours with copy number data is shown here. SNV mutation clusters in each tumour determined by Dirichlet clustering using PyClone coloured distinctly and labelled from 1 through 6. Shades of yellow are clusters shared between biopsies, greens are clusters only in the pre-treatment biopsy, and blues are clusters only in the post-treatment biopsies. Mutations in driver genes are labelled, where present. T1, pre-treatment biopsy; T2, post-treatment biopsy; CCF, cancer cell fraction.
(TIF)

S11 Fig Patterns of clonal evolution following afatinib monotherapy. Eight of 9 tumours display branched evolution, 1 tumour displayed shift in clonal structure. The intensity of blue shading corresponds to density of somatic mutations. T1, pre-treatment biopsy; T2, post-treatment biopsy; CCF, cancer cell fraction.
(TIF)

S1 Table Biopsy locations and HER2 central review results. ALN, axillary lymph node. Weak to moderate staining and strong membrane staining of HER2 in >10% of cells scored as IHC 2+ and 3+, respectively.
(DOCX)

S2 Table Sequencing statistics of IBC tumour biopsies and matched germline samples. (DOCX)

S3 Table Ploidy and purity of tumour biopsies. (DOCX)

S4 Table Clinical characteristics of TCGA patients used in this study. IDC, invasive ductal carcinoma; ILC, invasive lobular carcinoma.
(XLSX)

S5 Table Tumour response by individual patient. (DOCX)

S6 Table Somatic mutations (SNVs and indels) identified in IBC (n = 22). (XLSX)

S7 Table *TP53* mutations identified in IBC patients. (DOCX)

S8 Table Copy number profiles of pre-treatment biopsies in IBC (n = 20). (XLSX)

S9 Table GISTIC peaks identified in IBC (n = 20). (DOCX)

S10 Table Somatic mutations (SNVs and indels) identified in pre- and post-treatment biopsies (n = 13). (XLSX)

S11 Table Copy number profiles of post-treatment biopsies in IBC (n = 11). (XLSX)

S1 Text Clinical trial protocol. (PDF)

S2 Text TREND statement checklist. (PDF)

## Abbreviations

AE: adverse event
CBR: clinical benefit rate
CCF: cancer cell fraction
CR: complete response
ECOG: Eastern Cooperative Oncology Group
ER: oestrogen
FISH: fluorescence in situ hybridization
IBC: inflammatory breast cancer
IHC: immunohistochemistry
OR: objective response
OS: overall survival
PD: progressive disease
PFS: progression-free survival
PgR: progesterone
PR: partial response
RECIST: Response Evaluation Criteria in Solid Tumors
SCNA: somatic copy number alteration
SD: stable disease
SNV: single nucleotide variant
TCGA: The Cancer Genome Atlas
VAF: variant allele frequency
wGII: weighted Genomic Instability Index
