# Supplementary material for: Clonal Evolutionary Analysis during HER2 Blockade in HER2-Positive Inflammatory Breast Cancer: A Phase II Open-Label Clinical Trial of Afatinib +/- Vinorelbine
Source: PLoS Med. 2016 Dec 6;13(12):e1002136. doi: 10.1371/journal.pmed.1002136 (PMC5140058; doi:10.1371/journal.pmed.1002136)
Supplement: S1 Appendix — (PDF) [file pmed.1002136.s001.pdf]

NRES Committee London - Chelsea
Room 4W/12, 4th Floor West
Charing Cross Hospital
Fulham Palace Road
London W6 8RF

Telephone: 020 331 17251
Facsimile:

21 July 2011

[redacted]

[redacted]

**Study title:**	**1200.89: An open label, phase II trial of afatinib with or without vinorelbine for the treatment of HER2-overexpressing Inflammatory Breast Cancer**
**REC reference:**	**11/LO/0762**
**Protocol number:**	**1200.89**
**EudraCT number:**	**2010-024454-10**

Thank you for your letter of 14 July 2011, responding to the Committee's request for further information on the above research and submitting revised documentation.

The further information has been considered on behalf of the Committee by the Chair.

Confirmation of ethical opinion

On behalf of the Committee, I am pleased to confirm a **favourable ethical opinion** for the above research on the basis described in the application form, protocol and supporting documentation as revised, subject to the conditions specified below.

Ethical review of research sites

NHS sites

The favourable opinion applies to all NHS sites listed in the application, subject to management permission being obtained from the NHS/HSC R&D office prior to the start of the study (see "Conditions of the favourable opinion" below).

Non-NHS sites

The Committee has not yet been notified of the outcome of any site-specific assessment (SSA) for the non-NHS research site(s) taking part in this study. The favourable opinion does not therefore apply to any non-NHS site at present. We will write to you again as soon as one Research Ethics Committee has notified the outcome of a SSA. In the meantime no study procedures should be initiated at non-NHS sites.

Conditions of the favourable opinion

The favourable opinion is subject to the following conditions being met prior to the start of the study.

Management permission or approval must be obtained from each host organisation prior to the start of the study at the site concerned.

Management permission ("R&D approval") should be sought from all NHS organisations involved in the study in accordance with NHS research governance arrangements.

Guidance on applying for NHS permission for research is available in the Integrated Research Application System or at http://www.rdforum.nhs.uk.

Where a NHS organisation's role in the study is limited to identifying and referring potential participants to research sites ("participant identification centre"), guidance should be sought from the R&D office on the information it requires to give permission for this activity.

For non-NHS sites, site management permission should be obtained in accordance with the procedures of the relevant host organisation.

Sponsors are not required to notify the Committee of approvals from host organisations

Clinical trial authorisation must be obtained from the Medicines and Healthcare products Regulatory Agency (MHRA).

The sponsor is asked to provide the Committee with a copy of the notice from the MHRA, either confirming clinical trial authorisation or giving grounds for non-acceptance, as soon as this is available.

It is the responsibility of the sponsor to ensure that all the conditions are complied with before the start of the study or its initiation at a particular site (as applicable).

Approved documents

The final list of documents reviewed and approved by the Committee is as follows:

Document	*Version*	*Date*
Covering Letter		09 May 2011
Evidence of insurance or indemnity		08 April 2011
GP/Consultant Information Sheets	1	27 April 2011
Investigator CV		12 August 2009
Investigator's Brochure	11	05 January 2011
Letter from Sponsor		28 April 2011
Other: Summary of Product Characteristics		01 February 2010
Other: Study Contact details	1	19 March 2010
Other: MHRA Notice of Acceptance	00015/0359/001-0001	18 May 2011
Participant Consent Form	2	13 July 2011
Participant Information Sheet	2	13 July 2011
Protocol	1.0	03 March 2011
REC application	71700/212472/1/827	12 May 2011
Response to Request for Further Information		14 July 2011
Sample Diary/Patient Card	1	27 April 2011

Statement of compliance

This Committee is recognised by the United Kingdom Ethics Committee Authority under the Medicines for Human Use (Clinical Trials) Regulations 2004, and is authorised to carry out the ethical review of clinical trials of investigational medicinal products.

The Committee is fully compliant with the Regulations as they relate to ethics committees and the conditions and principles of good clinical practice.

The Committee is constituted in accordance with the Governance Arrangements for Research Ethics Committees (July 2001) and complies fully with the Standard Operating Procedures for Research Ethics Committees in the UK.

After ethical review

Reporting requirements

The attached document *"After ethical review – guidance for researchers"* gives detailed guidance on reporting requirements for studies with a favourable opinion, including:

- Notifying substantial amendments
- Adding new sites and investigators
- Notification of serious breaches of the protocol
- Progress and safety reports
- Notifying the end of the study

The NRES website also provides guidance on these topics, which is updated in the light of changes in reporting requirements or procedures.

Feedback

You are invited to give your view of the service that you have received from the National Research Ethics Service and the application procedure. If you wish to make your views known please use the feedback form available on the website.

Further information is available at National Research Ethics Service website > After Review

**11/LO/0762**	**Please quote this number on all correspondence**

With the Committee's best wishes for the success of this project

Yours sincerely

[redacted]

Enclosures: "After ethical review – guidance for researchers" *[SL-AR1]*

[redacted]

NHS
National Research Ethics Service

NRES Committee London - Chelsea
Room 4W/12, 4th Floor West
Charing Cross Hospital
Fulham Palace Road
London W6 8RF
Tel: 020 331 17251

13 October 2011

[redacted]

**Study title:**	**1200.89: An open label, phase II trial of afatinib with or without vinorelbine for the treatment of HER2-overexpressing Inflammatory Breast Cancer**
**REC reference:**	**11/LO/0762**
**Protocol number:**	**1200.89**
**EudraCT number:**	**2010-024454-10**
**Amendment number:**	**AM1**
**Amendment date:**	**27 September 2011**

The above amendment was reviewed by the Sub-Committee in correspondence.

Ethical opinion

The members of the Committee taking part in the review gave **a favourable ethical opinion of the amendment** on the basis described in the notice of amendment form and supporting documentation.

Approved documents

The documents reviewed and approved at the meeting were:

Document	Version	Date
Participant Consent Form: tracked & clean	3	27 September 2011
Participant Information Sheet: tracked & clean	3	27 September 2011
Investigator CV	[redacted]	04 May 2011
European Commission Notification of Substantial Amendment Form	AM1	27 September 2011

Membership of the Committee

The members of the Committee who took part in the review are listed overleaf.

R&D approval

All investigators and research collaborators in the NHS should notify the R&D office for the relevant NHS care organisation of this amendment and check whether it affects R&D approval of the research.

Statement of compliance

This Committee is recognised by the United Kingdom Ethics Committee Authority under the Medicines for Human Use (Clinical Trials) Regulations 2004, and is authorised to carry out the ethical review of clinical trials of investigational medicinal products.

The Committee is fully compliant with the Regulations as they relate to ethics committees and the conditions and principles of good clinical practice.

The Committee is constituted in accordance with the Governance Arrangements for Research Ethics Committees (July 2001) and complies fully with the Standard Operating Procedures for Research Ethics Committees in the UK.

**11/LO/0762:**	**Please quote this number on all correspondence**

Yours sincerely

[redacted]

NRES Committee London - Chelsea
Attendance at Sub-Committee of the REC meeting on 10 October 2011

Name	*Profession*	*Capacity*
[redacted]	[redacted]	Expert
		Expert

NHS
Health Research Authority

NRES Committee London - Chelsea
Room 4W/12, 4th Floor West
Charing Cross Hospital
Fulham Palace Road
London W6 8RF

Tel: 020 331 17251
Fax:

13 February 2012

[redacted]

**Study title:**	**1200.89: An open label, phase II trial of afatinib with or without vinorelbine for the treatment of HER2-overexpressing Inflammatory Breast Cancer**
**REC reference:**	**11/LO/0762**
**Protocol number:**	**1200.89**
**EudraCT number:**	**2010-024454-10**
**Amendment number:**	
**Amendment date:**	**02 February 2012**

The above amendment was reviewed by the Sub-Committee in correspondence.

Ethical opinion

The members of the Committee taking part in the review gave a favourable ethical opinion of the amendment on the basis described in the notice of amendment form and supporting documentation.

Approved documents

The documents reviewed and approved at the meeting were:

Document	Version	Date
History sheet - changes from version 11 to version 12		
Participant Consent Form: with changes tracked	4	23 January 2012
Participant Consent Form	4	23 January 2012
Participant Information Sheet: with changes tracked	4	23 January 2011
Participant Information Sheet	4	23 January 2012
Investigator's Brochure	12	02 January 2012
European Commission Notification of Substantial Amendment Form		02 February 2012
Covering Letter		02 February 2012

Membership of the Committee

The members of the Committee who took part in the review were [redacted]

R&D approval

All investigators and research collaborators in the NHS should notify the R&D office for the relevant NHS care organisation of this amendment and check whether it affects R&D approval of the research.

Statement of compliance

This Committee is recognised by the United Kingdom Ethics Committee Authority under the Medicines for Human Use (Clinical Trials) Regulations 2004, and is authorised to carry out the ethical review of clinical trials of investigational medicinal products.

The Committee is fully compliant with the Regulations as they relate to ethics committees and the conditions and principles of good clinical practice.

The Committee is constituted in accordance with the Governance Arrangements for Research Ethics Committees and complies fully with the Standard Operating Procedures for Research Ethics Committees in the UK.

**11/LO/0762:**	**Please quote this number on all correspondence**

Yours sincerely

[redacted]

NRES Committee London - Chelsea

Attendance at Sub-Committee of the REC meeting on 08 February 2012

Name	*Profession*	*Capacity*
[redacted]	[redacted]	Lay Plus
[redacted]	[redacted]	Expert

NHS
Health Research Authority
NRES Committee London - Chelsea
Room 4W/12, 4th Floor West
Charing Cross Hospital
Fulham Palace Road
London W6 8RF

Telephone: 020 331 17282

07 March 2012

[redacted]

**Study Title:**	**1200.89: An open label, phase II trial of afatinib with or without vinorelbine for the treatment of HER2-overexpressing Inflammatory Breast Cancer**
**REC reference:**	**11/LO/0762**
**Protocol number:**	**1200.89**
**EudraCT number:**	**2010-024454-10**
**Amendment number:**	**3**
**Amendment date:**	**07 March 2012**

Thank you for submitting the above amendment, which was received on 07 March 2012.

Research Site	Principal Investigator / Local Collaborator
University College London Hospital, Cancer Clinical Research Facility, Ground Floor EGA Wing 235 Euston Road, London NW1 2BU	[redacted]

The amendment relates solely to the addition of new site(s) and/or investigator(s) within the National Health Service (NHS) or Health and Social Care (HSC) in Northern Ireland. The site-specific assessment for the site(s) will therefore form part of the research governance review. The Site-Specific Information (SSI) Form for the site should be included with the application for R&D approval.

On behalf of the Committee, I am pleased to confirm the extension of the favourable opinion to the new site(s) and/or investigator(s), subject to management permission being given by the relevant NHS/HSC R&D office(s) prior to the study starting at the site.

Statement of compliance

This Committee is recognised by the United Kingdom Ethics Committee Authority under the Medicines for Human Use (Clinical Trials) Regulations 2004, and is authorised to carry out the ethical review of clinical trials of investigational medicinal products. The Committee is fully compliant with the Regulations as they relate to ethics committees and the conditions and principles of good clinical practice.

The Committee is constituted in accordance with the Governance Arrangements for Research Ethics Committees and complies fully with the Standard Operating Procedures for Research Ethics Committees in the UK.

**11/LO/0762**	**Please quote this number on all correspondence**

Yours sincerely

[redacted]

NHS
Health Research Authority

NRES Committee London - Chelsea

HRA
Research Ethics Committee (REC) London Centre
Ground Floor
80 Skipton House
London Road
London, SE1 6LH

Tel: 020 7972 2556

10 December 2012

[redacted]

**Study title:**	**1200.89: An open label, phase II trial of afatinib with or without vinorelbine for the treatment of HER2-overexpressing Inflammatory Breast Cancer**
**REC reference:**	**11/LO/0762**
**Protocol number:**	**1200.89**
**EudraCT number:**	**2010-024454-10**
**Amendment number:**	**Substantial Amendment 4 dated 08 November 2012**
**IRAS project ID:**	**71700**

The above amendment was reviewed by the Sub-Committee in correspondence.

Ethical opinion

The members of the Committee taking part in the review gave a favourable ethical opinion of the amendment on the basis described in the notice of amendment form and supporting documentation.

Approved documents

The documents reviewed and approved at the meeting were:

Document	Version	Date
Product Information - Navelbine (vinorelbine)		
Participant Consent Form	5	08 November 2012
Participant Information Sheet	5	08 November 2012
Protocol	2.0	18 October 2012
European Commission Notification of Substantial Amendment Form	Substantial Amendment 4 dated 08 November 2012	08 November 2012
Covering Letter		08 November 2012

Membership of the Committee

The members of the Committee who took part in the review are listed on the attached sheet.

R&D approval

All investigators and research collaborators in the NHS should notify the R&D office for the relevant NHS care organisation of this amendment and check whether it affects R&D approval of the research.

Statement of compliance

This Committee is recognised by the United Kingdom Ethics Committee Authority under the Medicines for Human Use (Clinical Trials) Regulations 2004, and is authorised to carry out the ethical review of clinical trials of investigational medicinal products.

The Committee is fully compliant with the Regulations as they relate to ethics committees and the conditions and principles of good clinical practice.

The Committee is constituted in accordance with the Governance Arrangements for Research Ethics Committees and complies fully with the Standard Operating Procedures for Research Ethics Committees in the UK.

We are pleased to welcome researchers and R & D staff at our NRES committee members' training days – see details at http://www.hra.nhs.uk/hra-training/

11/LO/0762:	Please quote this number on all correspondence

Yours sincerely

[redacted]

NRES Committee London - Chelsea

Attendance at Sub-Committee of the REC meeting on 16 November 2012

Name	*Profession*	*Capacity*
[redacted]	[redacted]	Expert
[redacted]	[redacted]	Lay Plus

NHS
Health Research Authority
NRES Committee London - Chelsea
HRA
Research Ethics Committee (REC) London Centre
Ground Floor
80 Skipton House
London Road
London, SE1 6LH

Tel: 020 7972 2556

29 August 2013

[redacted]

**Study title:**	**1200.89: An open label, phase II trial of afatinib with or without vinorelbine for the treatment of HER2-overexpressing Inflammatory Breast Cancer**
**REC reference:**	**11/LO/0762**
**Protocol number:**	**1200.89**
**EudraCT number:**	**2010-024454-10**
**Amendment number:**	**Substantial Amendment 6 - 23 July 2013**
**IRAS project ID:**	**71700**

The above amendment was reviewed by the Sub-Committee in correspondence.

Ethical opinion

The members of the Committee taking part in the review gave a favourable ethical opinion of the amendment on the basis described in the notice of amendment form and supporting documentation.

Approved documents

The documents reviewed and approved at the meeting were:

Document	Version	Date
Addendum 1 - Supporting Document for Afatinib Risks		
European Commission Notification of Substantial Amendment Form	Substantial Amendment 6 - 23 July 2013	23 July 2013
History Sheet for IB		04 July 2013
Investigator's Brochure	14	11 July 2013
Participant Information Sheet: Addendum 1 - New Information for Ongoing Patients	1	18 July 2013
Covering Letter		23 July 2013
Protocol	3.0	01 July 2013

Evidence of insurance or indemnity		17 July 2012

Membership of the Committee

The members of the Committee who took part in the review are listed on the attached sheet.

R&D approval

All investigators and research collaborators in the NHS should notify the R&D office for the relevant NHS care organisation of this amendment and check whether it affects R&D approval of the research.

Statement of compliance

This Committee is recognised by the United Kingdom Ethics Committee Authority under the Medicines for Human Use (Clinical Trials) Regulations 2004, and is authorised to carry out the ethical review of clinical trials of investigational medicinal products.

The Committee is fully compliant with the Regulations as they relate to ethics committees and the conditions and principles of good clinical practice.

The Committee is constituted in accordance with the Governance Arrangements for Research Ethics Committees and complies fully with the Standard Operating Procedures for Research Ethics Committees in the UK.

We are pleased to welcome researchers and R & D staff at our NRES committee members' training days – see details at http://www.hra.nhs.uk/hra-training/

**11/LO/0762:**	**Please quote this number on all correspondence**

Yours sincerely

[redacted]

NRES Committee London - Chelsea

Attendance at Sub-Committee of the REC meeting on 09 August 2013

Name	*Profession*	*Capacity*
[redacted]	[redacted]	Lay
[redacted]	[redacted]	Expert

Also in attendance:

Name	*Position (or reason for attending)*
[redacted]	[redacted]

NRES Committee London - Chelsea
Research Ethics Committee (REC) Bristol Centre
Level 3, Block B
Whitefriars
Lewins Mead
Bristol
BS1 2NT

Tel: 0117 342 1380

20 May 2014

[redacted]

Study title: **1200.89: An open label, phase II trial of afatinib with or without vinorelbine for the treatment of HER2-overexpressing Inflammatory Breast Cancer**
REC reference: **11/LO/0762**
Protocol number: **1200.89**
EudraCT number: **2010-024454-10**
Amendment number: **Minor Amendment 2**
Amendment date: **12 May 2014**
IRAS project ID: **71700**

Thank you for your letter of 12 May 2014, notifying the Committee of the above amendment.

It is noted that you do not consider this to be a substantial amendment to the clinical trial authorisation, as defined in the Medicines for Human Use (Clinical Trials) Regulations 2004, and that ethical review by the Committee is therefore not required.

Documents received

The documents received were as follows:

Document	*Version*	*Date*
Letter from sponsor [Sponsor Confirmation Minor Amendment]		19 May 2014
Notice of Minor Amendment [Minor Amendment 2]		12 May 2014
Research protocol or project proposal [Internal Signature Page]	4.0	14 April 2014

Research protocol or project proposal [(tracked copy)]	4.0	10 April 2014
Research protocol or project proposal [CI Signature Page]	4.0	17 April 2014

Statement of compliance

This Committee is recognised by the United Kingdom Ethics Committee Authority under the Medicines for Human Use (Clinical Trials) Regulations 2004, and is authorised to carry out the ethical review of clinical trials of investigational medicinal products.

The Committee is fully compliant with the Regulations as they relate to ethics committees and the conditions and principles of good clinical practice.

The Committee is constituted in accordance with the Governance Arrangements for Research Ethics Committees and complies fully with the Standard Operating Procedures for Research Ethics Committees in the UK.

**11/LO/0762:**	**Please quote this number on all correspondence**

Yours sincerely

[redacted]
