# Supplementary figures and images for: Clonal Evolutionary Analysis during HER2 Blockade in HER2-Positive Inflammatory Breast Cancer: A Phase II Open-Label Clinical Trial of Afatinib +/- Vinorelbine
Source: PLoS Med. 2016 Dec 6;13(12):e1002136. doi: 10.1371/journal.pmed.1002136 (PMC5140058; doi:10.1371/journal.pmed.1002136)

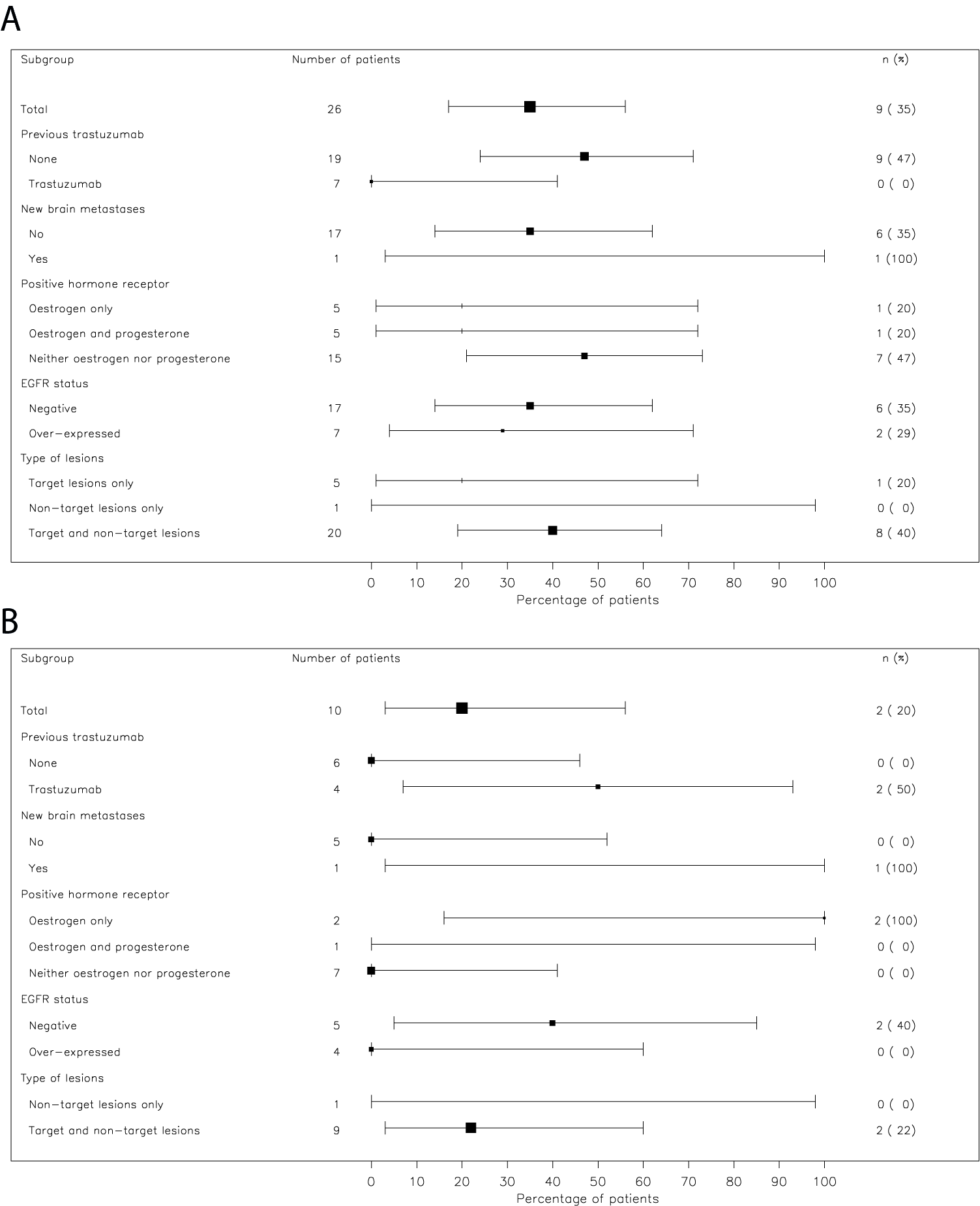

Supplement: S1 Fig — (A) Subgroup analyses for Part A. (B) Subgroup analyses for Part B. (TIF) [file pmed.1002136.s002.tif]

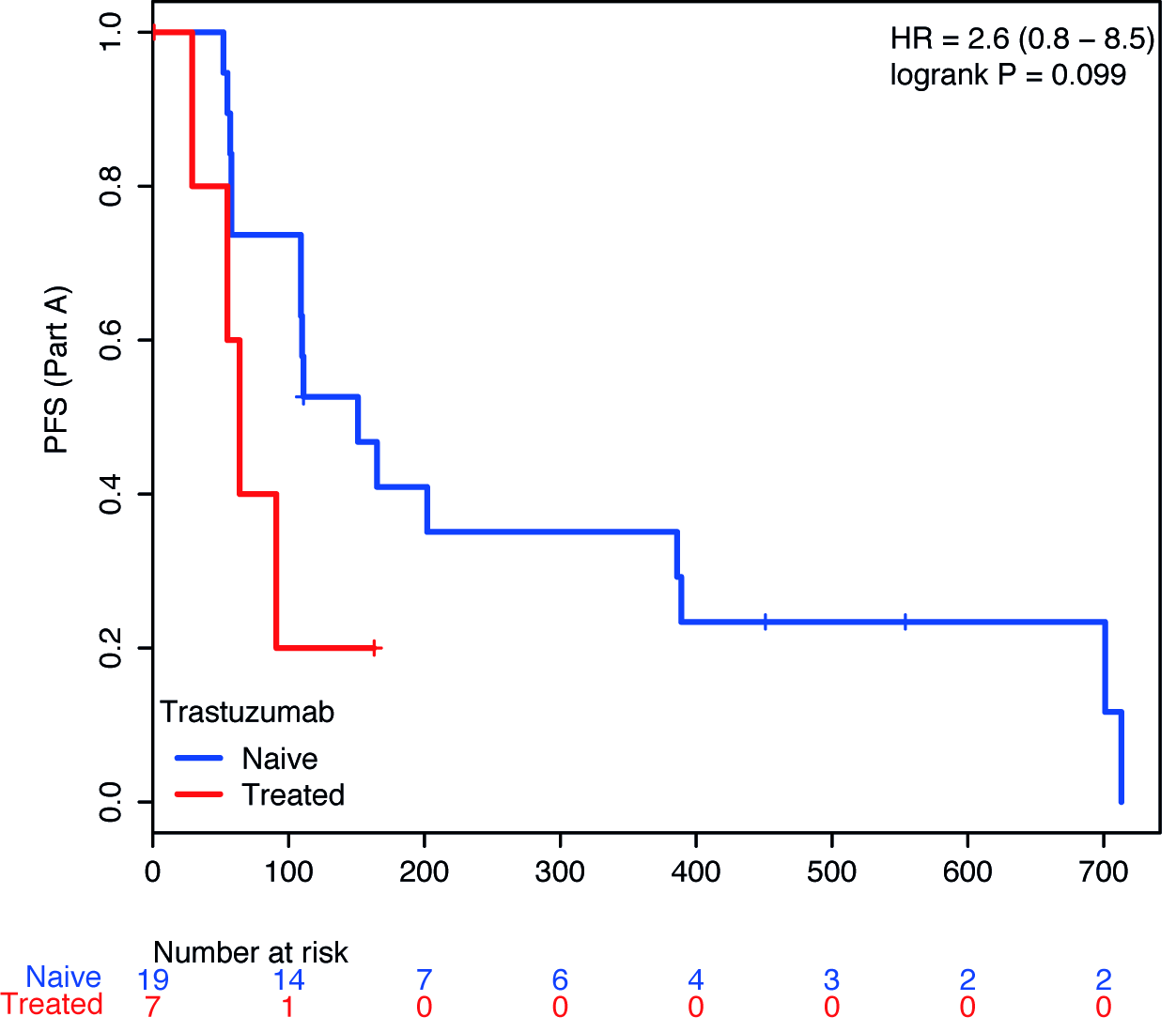

Supplement: S2 Fig — Y-axis is percentage PFS, x-axis is time to PD or death (days). Blue line, trastuzumab-naïve patients; red line, trastuzumab-treated patients. (TIF) [file pmed.1002136.s003.tif]

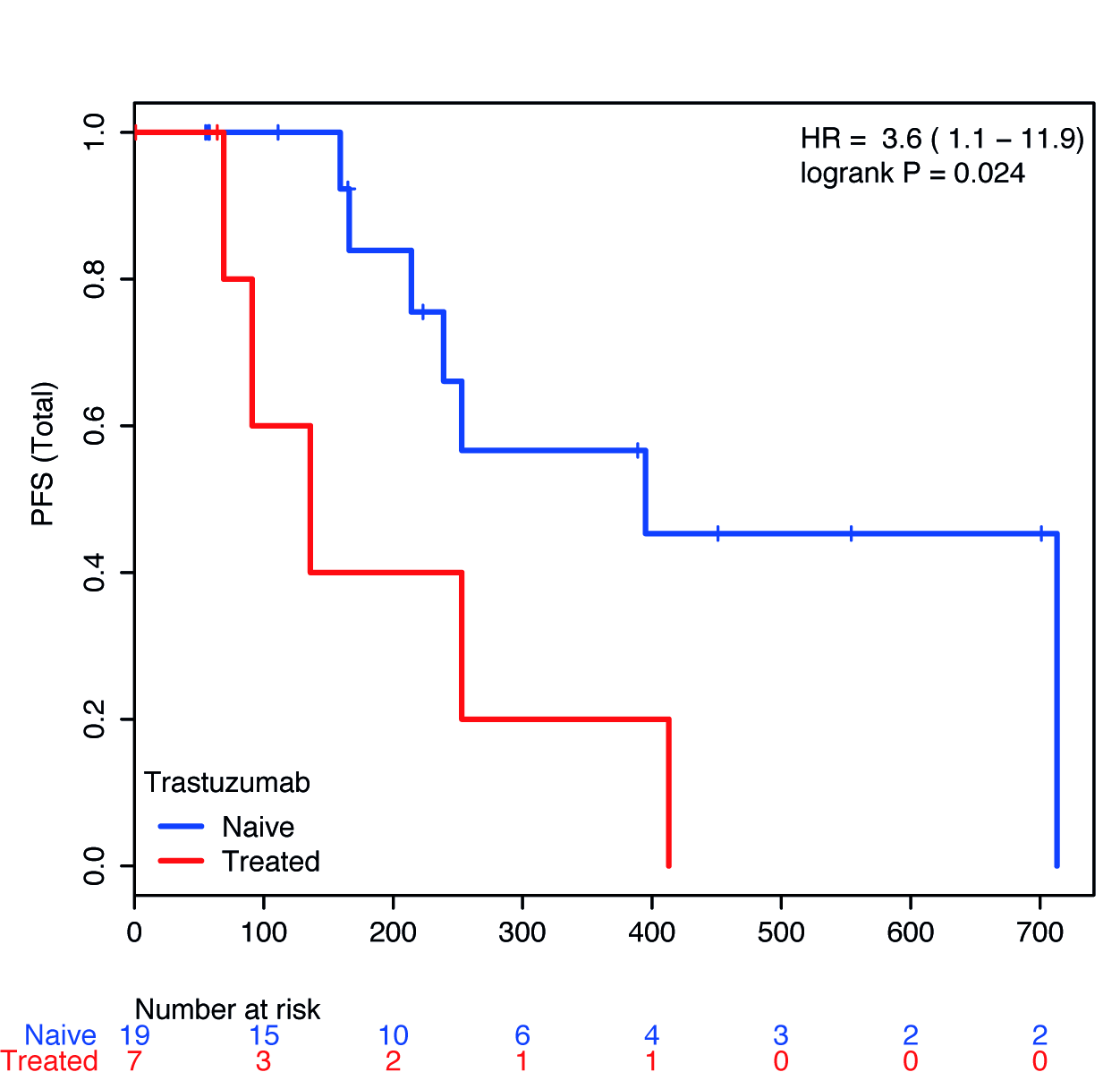

Supplement: S3 Fig — Y-axis is percentage PFS, x-axis is time to PD or death (days). Blue line, trastuzumab-naïve patients; red line, trastuzumab-treated patients. (TIF) [file pmed.1002136.s004.tif]

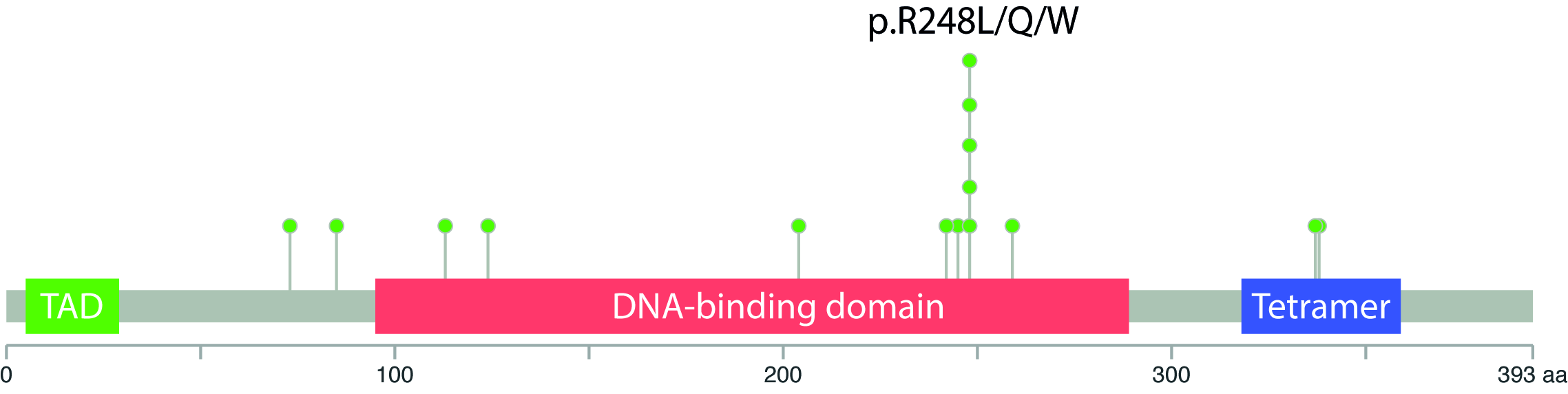

Supplement: S4 Fig — Each missense, nonsense and frameshift mutation is depicted as a green circle; splice site mutations are not shown. Recurrent gain-of-function p.R248 mutations are labelled. TAD, transcription-activation domain. (TIF) [file pmed.1002136.s005.tif]

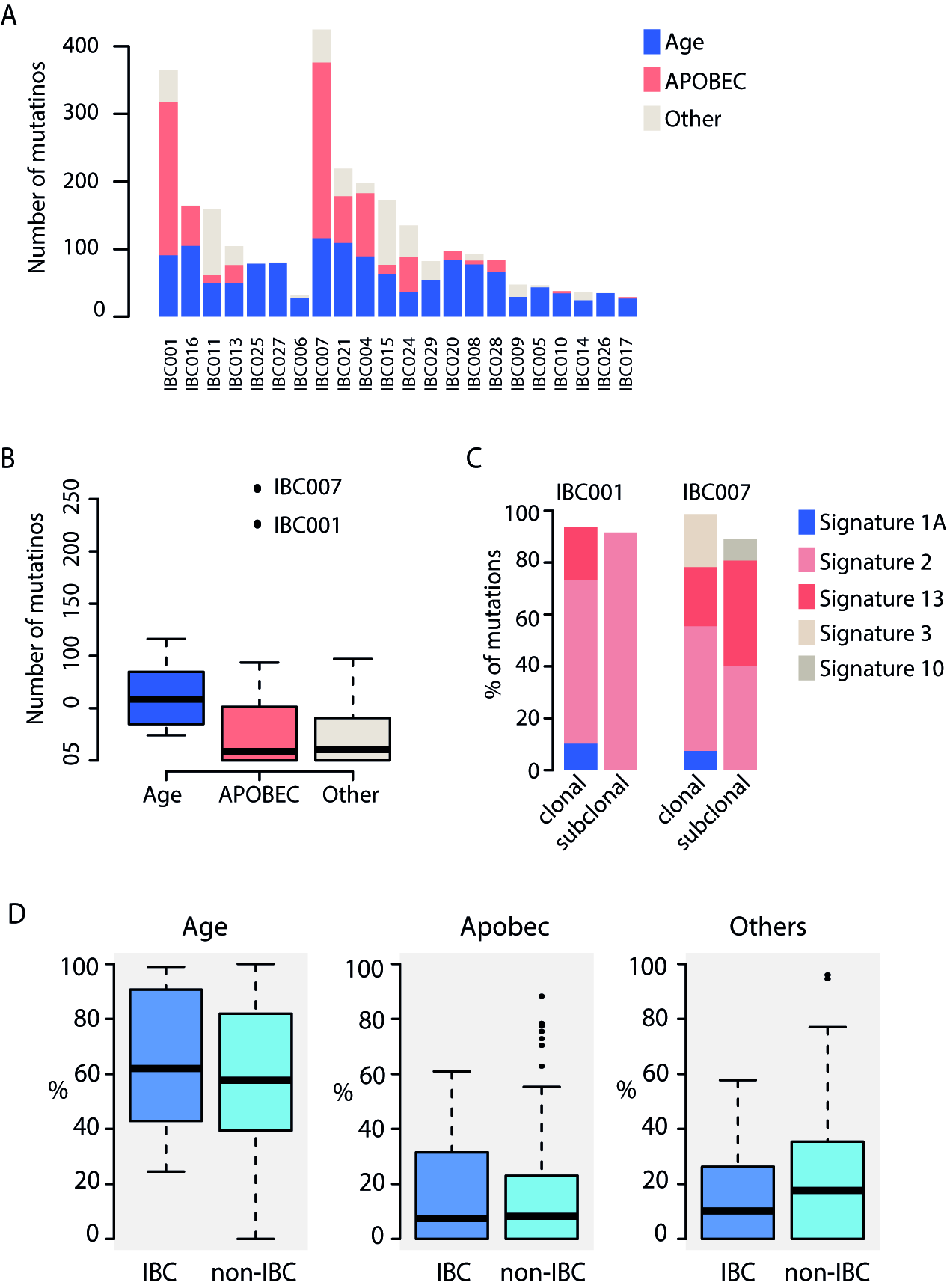

Supplement: S5 Fig — (A) Breakdown of mutations driven by age, APOBEC and other mutational processes by patient. (B) Boxplots of number of mutations explained by age, APOBEC and other mutational signatures. (C) Breakdown of mutational signatures in IBC001 and IBC007. (D) Boxplot of different contributions of age, APOBEC-related and other mutational signatures in IBC versus non-IBC tumours. (TIF) [file pmed.1002136.s006.tif]

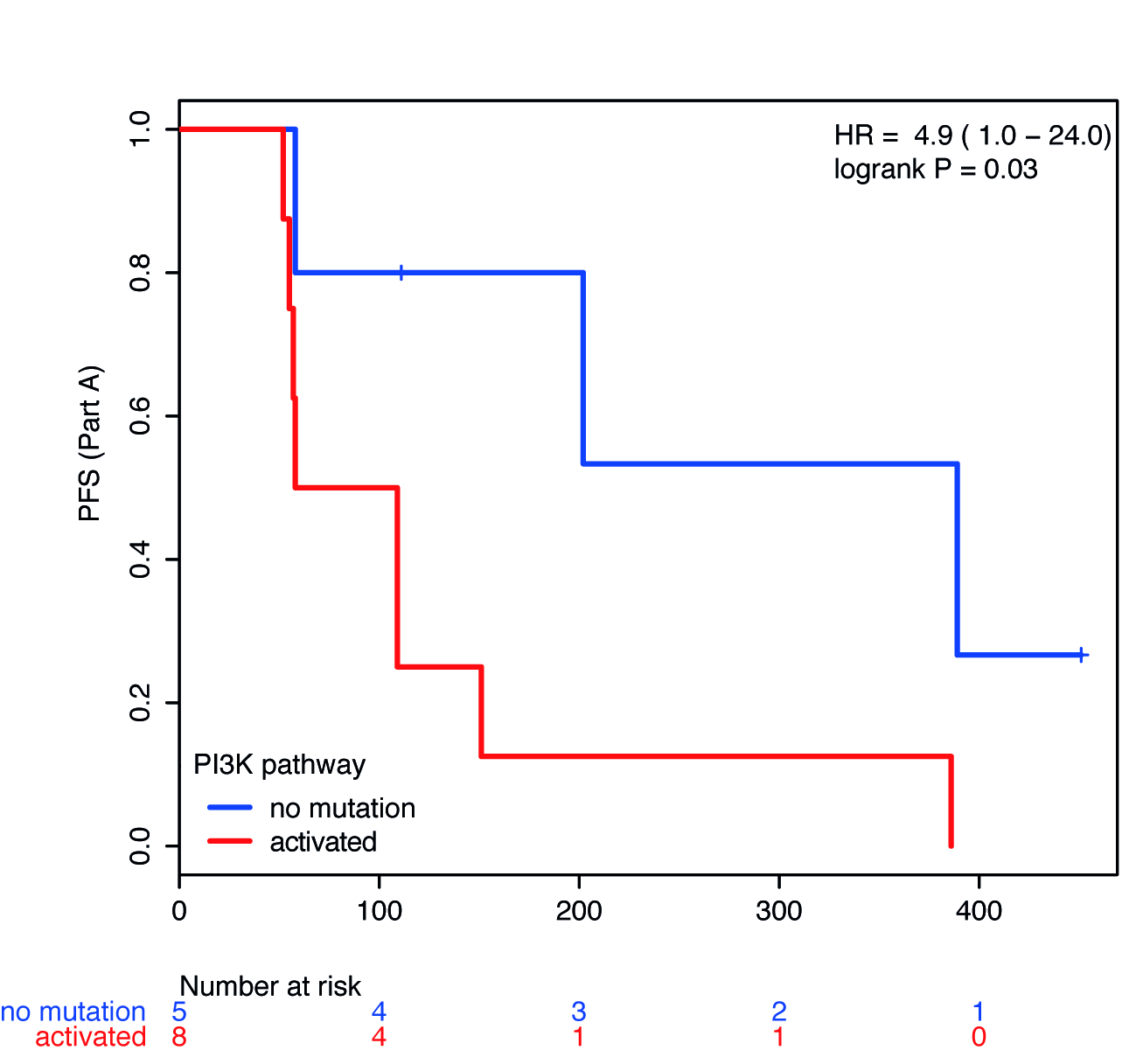

Supplement: S6 Fig — Activation of PI3K/Akt pathway defined as PI3KCA amplification or activating mutation, PTEN loss and/or activating mutations in AKT1 and ERBB2. Y-axis is percentage PFS, x-axis is time to PD or death (days). Blue line, patients without mutations in PI3K/Akt pathway; red line, patients with somatic activation in PI3K/Akt pathway. (TIF) [file pmed.1002136.s007.tif]

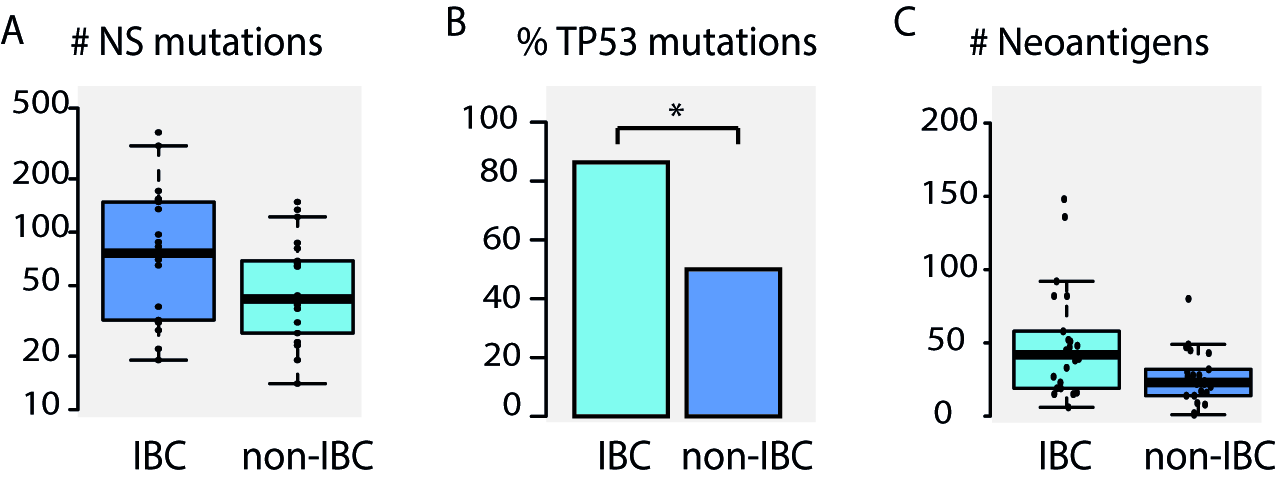

Supplement: S7 Fig — (A) Boxplot showing higher numbers of somatic nonsynonymous (NS) mutations identified in IBC patients compared to non-IBC patients. (B) Barplot showing an enrichment of TP53 mutations in IBC patients versus non-IBC patients. (C) Boxplot showing higher numbers of neoantigens predicted in IBC patients compared to non-IBC patients. (TIF) [file pmed.1002136.s008.tif]

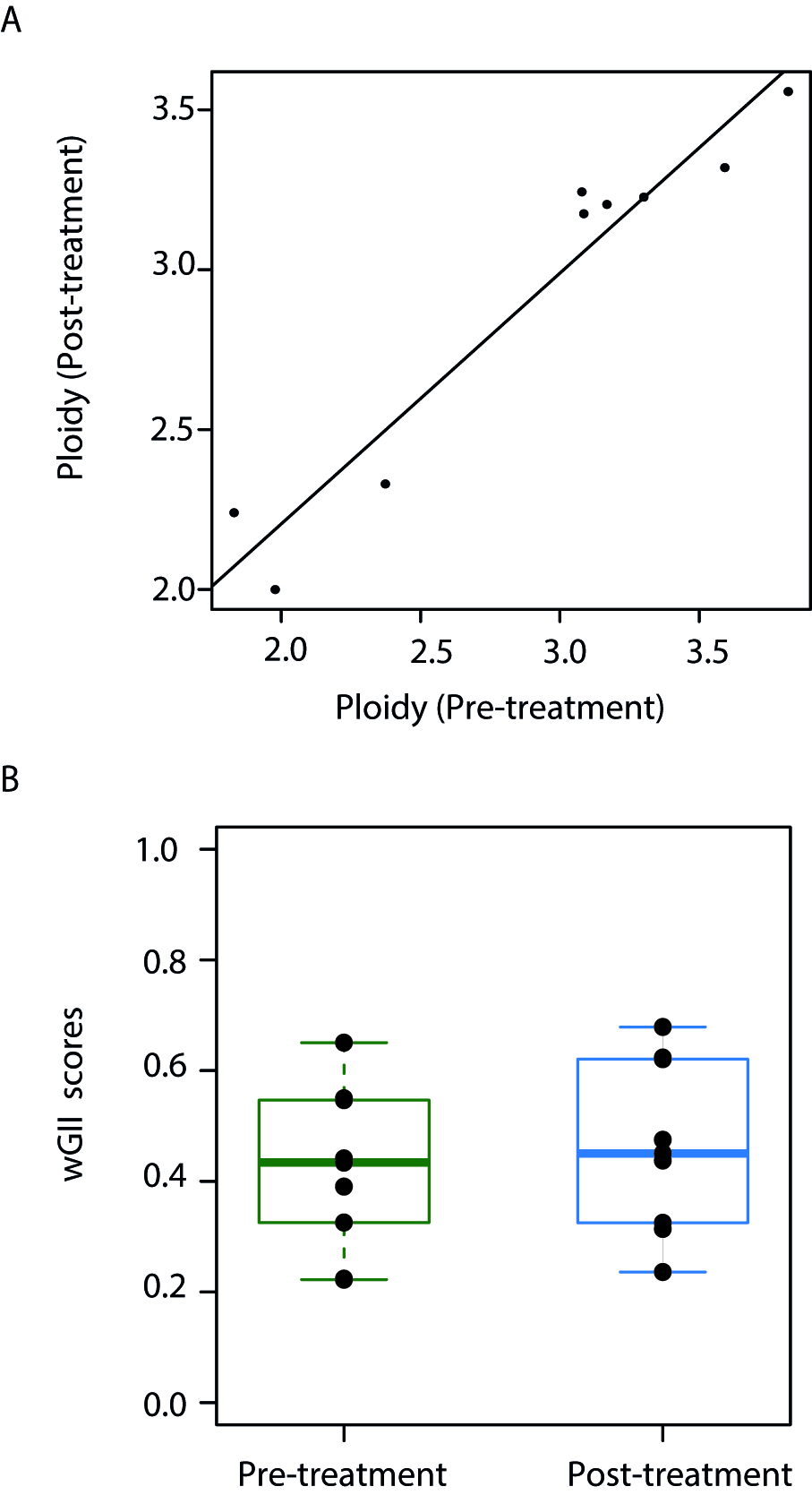

Supplement: S8 Fig — (A) Ploidy scores in pre-treatment (x-axis) and post-treatment (y-axis) tumours. Line represents linear regression fit. (B) wGII scores in pre- and post-treatment tumours. (TIF) [file pmed.1002136.s009.tif]

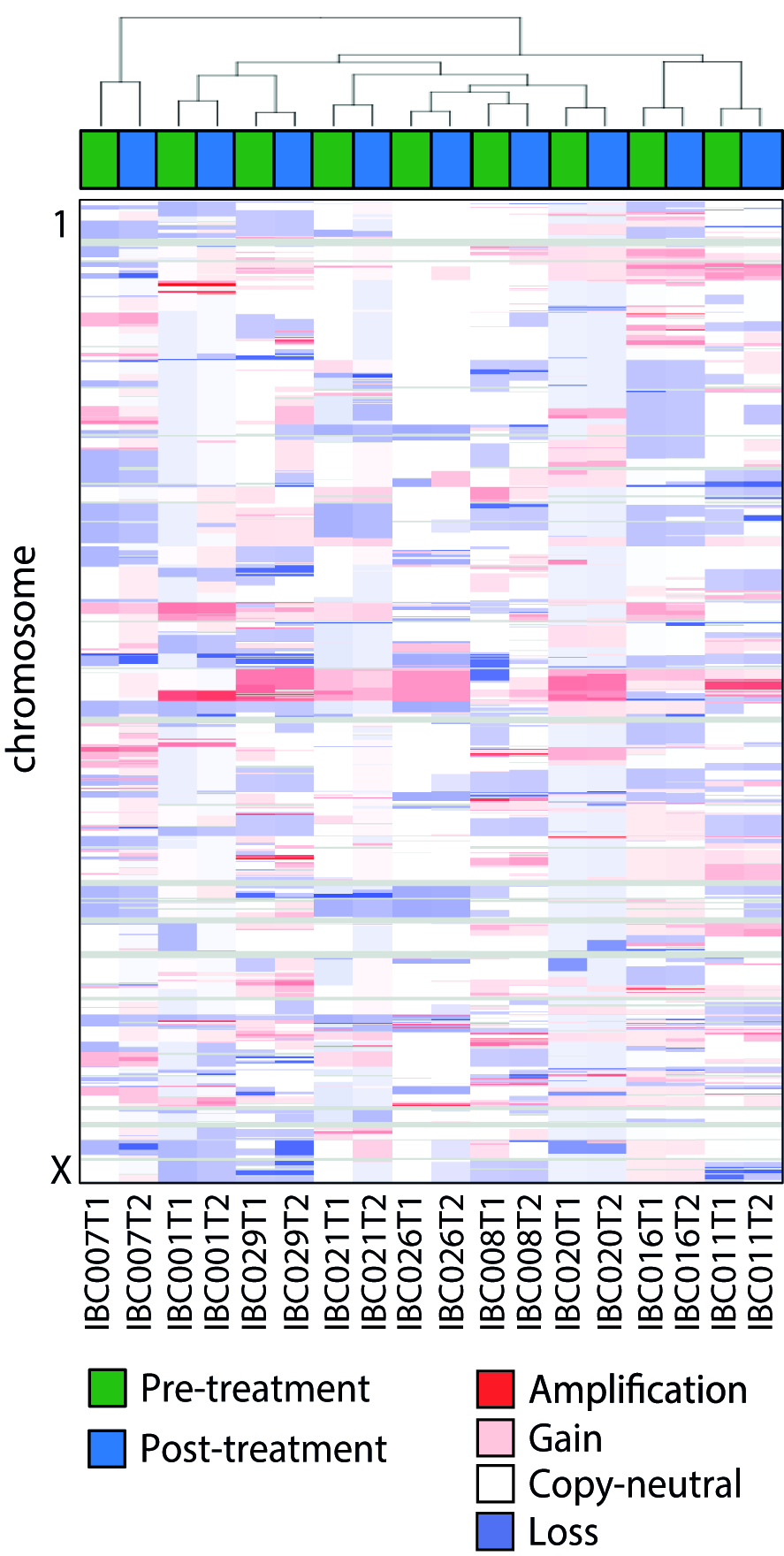

Supplement: S9 Fig — Pre-treatment biopsies are labelled T1 and in green; post-treatment biopsies are labelled T2 and in blue. (TIF) [file pmed.1002136.s010.tif]

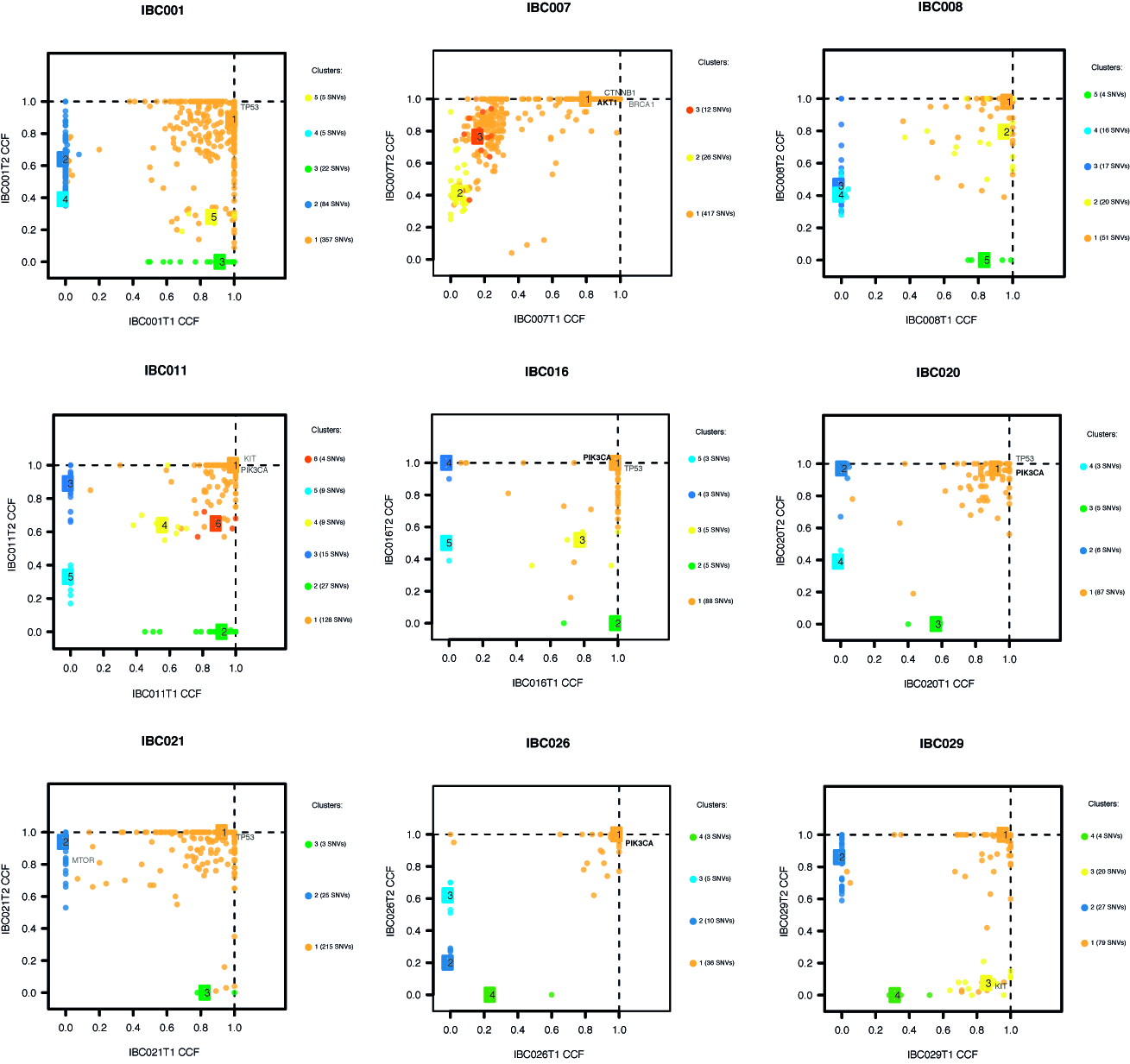

Supplement: S10 Fig — Each of the nine tumours with copy number data is shown here. SNV mutation clusters in each tumour determined by Dirichlet clustering using PyClone coloured distinctly and labelled from 1 through 6. Shades of yellow are clusters shared between biopsies, greens are clusters only in the pre-treatment biopsy, and blues are clusters only in the post-treatment biopsies. Mutations in driver genes are labelled, where present. T1, pre-treatment biopsy; T2, post-treatment biopsy; CCF, cancer cell fraction. (TIF) [file pmed.1002136.s011.tif]

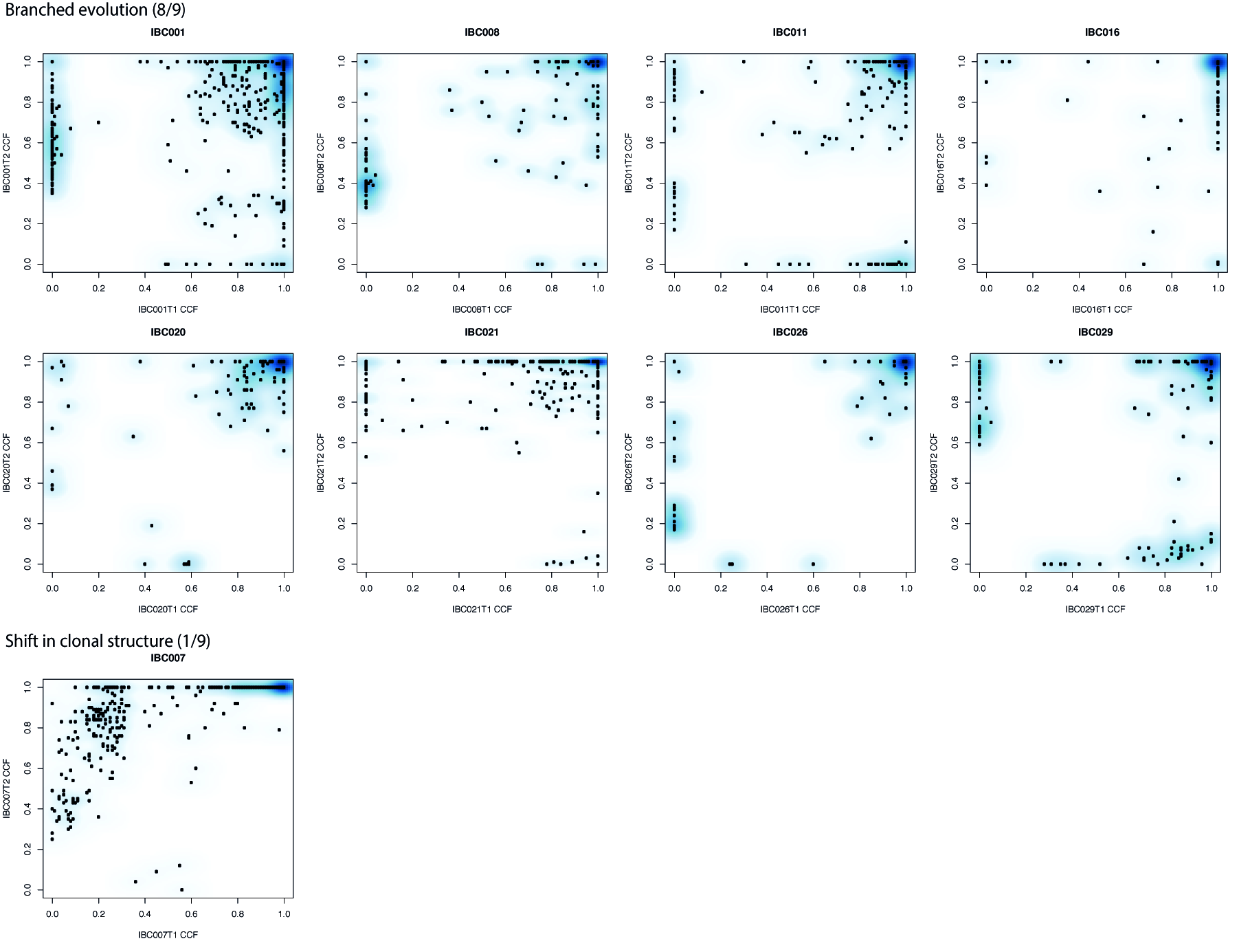

Supplement: S11 Fig — Eight of 9 tumours display branched evolution, 1 tumour displayed shift in clonal structure. The intensity of blue shading corresponds to density of somatic mutations. T1, pre-treatment biopsy; T2, post-treatment biopsy; CCF, cancer cell fraction. (TIF) [file pmed.1002136.s012.tif]
