# Supplementary material for: Clonal Evolutionary Analysis during HER2 Blockade in HER2-Positive Inflammatory Breast Cancer: A Phase II Open-Label Clinical Trial of Afatinib +/- Vinorelbine
Source: PLoS Med. 2016 Dec 6;13(12):e1002136. doi: 10.1371/journal.pmed.1002136 (PMC5140058; doi:10.1371/journal.pmed.1002136)
Supplement: S1 Table — ALN, axillary lymph node. Weak to moderate staining and strong membrane staining of HER2 in >10% of cells scored as IHC 2+ and 3+, respectively. (DOCX) [file pmed.1002136.s013.docx]

# S1 Table. Biopsy locations and HER2 central review results.

| Patient | Biopsy location | | HER2 results | |
| --- | --- | --- | --- | --- |
|  | **Baseline** | **After afatinib monotherapy** | **IHC** | **FISH** |
| IBC001 | Chest wall | Chest wall | 3 | Positive |
| IBC002 | Left breast | Breast | 2 | Positive |
| IBC003 | Left breast | ALN | 3 | Positive |
| IBC004 | Chest wall | Not taken | 2 | Positive |
| IBC005 | Unknown | Not taken | 3 | Positive |
| IBC006 | Right breast | Not taken | NA | NA |
| IBC007 | Left breast | Left breast | 2 | Positive |
| IBC008 | Unknown | Chest wall | 3 | Positive |
| IBC009 | Chest wall | Chest wall | 3 | Positive |
| IBC010 | Chest wall | Chest wall | 3 | Positive |
| IBC011 | Chest wall | Chest wall | 2 | Positive |
| IBC012 | Left breast | Left breast | 3 | Positive |
| IBC013 | Chest wall | Not taken | 3 | Positive |
| IBC014 | Breast | Liver | 3 | Positive |
| IBC015 | Chest wall | Chest wall | 3 | Positive |
| IBC016 | Right ALN | Left ALN | 3 | Positive |
| IBC017 | Unknown | Not taken | 3 | Positive |
| IBC020 | Chest wall | Chest wall | 3 | Positive |
| IBC021 | Chest wall | Chest wall | 3 | Positive |
| IBC022 | Chest wall | Not taken | 3 | Positive |
| IBC024 | Left breast | Not taken | 3 | Positive |
| IBC025 | Breast/ALN | Breast | 3 | Positive |
| IBC026 | Unknown | Left breast | NA | NA |
| IBC027 | Breast | Not taken | 3 | Positive |
| IBC028 | Chest wall | Chest wall | 2 | Positive |
| IBC029 | Left breast | Chest wall | 2 | Negative |

ALN, axillary lymph node.
