# Supplementary material for: Clonal Evolutionary Analysis during HER2 Blockade in HER2-Positive Inflammatory Breast Cancer: A Phase II Open-Label Clinical Trial of Afatinib +/- Vinorelbine
Source: PLoS Med. 2016 Dec 6;13(12):e1002136. doi: 10.1371/journal.pmed.1002136 (PMC5140058; doi:10.1371/journal.pmed.1002136)
Supplement: S2 Table — (DOCX) [file pmed.1002136.s014.docx]

# S2 Table. Sequencing statistics of IBC tumour biopsies and matched germline samples.

|  | Blood | Pre-afatinib tumour | Post-afatinb tumour |
| --- | --- | --- | --- |
| Total effective reads (x10^6^) | 1.56 | 3.89 | 4.24 |
| Total effective yield (Mb) | 13855 | 34665 | 37830 |
| Fraction of uniquely mapped on target | 70.40% | 66.80% | 69.50% |
| Average sequencing depth on target (x) | 157.9 | 372 | 425.4 |
| Mismatch rate in target region | 0.20% | 0.20% | 0.20% |
| Coverage of target region | 99.10% | 99.60% | 99.50% |
| Fraction of target covered with ≥20x | 90.50% | 95.80% | 96.10% |
| Fraction of target covered with ≥10x | 94.60% | 97.80% | 97.90% |
| Fraction of target covered with ≥4x | 97.60% | 99.00% | 99.00% |
| Mapping rate | 99.40% | 99.10% | 99.40% |
| Duplicate rate | 8.10% | 18.00% | 23.60% |
