# Supplementary material for: Clonal Evolutionary Analysis during HER2 Blockade in HER2-Positive Inflammatory Breast Cancer: A Phase II Open-Label Clinical Trial of Afatinib +/- Vinorelbine
Source: PLoS Med. 2016 Dec 6;13(12):e1002136. doi: 10.1371/journal.pmed.1002136 (PMC5140058; doi:10.1371/journal.pmed.1002136)
Supplement: S3 Table — (DOCX) [file pmed.1002136.s015.docx]

# S3 Table. Ploidy and purity of tumour biopsies.

| Tumour | Pre-afatinib biopsy | | Post-afatinib biopsy | |
| --- | --- | --- | --- | --- |
|  | **Purity** | **Ploidy** | **Purity** | **Ploidy** |
| IBC001 | 0.28 | 3.6 | 0.32 | 3.3 |
| IBC007 | 0.30 | 1.8 | 0.68 | 2.2 |
| IBC008 | 0.37 | 3.1 | 0.49 | 3.2 |
| IBC009 | 0.15 | 3.8 | NA* | NA* |
| IBC010 | NA* | NA* | 0.16 | 4.3 |
| IBC011 | 0.46 | 3.1 | 0.67 | 3.2 |
| IBC014 | NA* | NA* | 0.35 | 3.1 |
| IBC016 | 0.20 | 3.3 | 0.42 | 3.2 |
| IBC020 | 0.35 | 2.4 | 0.40 | 2.3 |
| IBC021 | 0.15 | 3.8 | 0.33 | 3.6 |
| IBC025 | 0.28 | 2.9 | NA* | NA* |
| IBC026 | 0.52 | 2.0 | 0.64 | 2.0 |
| IBC029 | 0.66 | 3.2 | 0.59 | 3.2 |
| IBC004 | 0.64 | 3.2 | NA | NA |
| IBC005 | 0.19 | 1.8 | NA | NA |
| IBC006 | 0.20 | 3.2 | NA | NA |
| IBC013 | 0.19 | 3.2 | NA | NA |
| IBC015 | 0.19 | 3.5 | NA | NA |
| IBC017 | 0.23 | 2.1 | NA | NA |
| IBC024 | 0.28 | 1.9 | NA | NA |
| IBC027 | 0.37 | 1.9 | NA | NA |
| IBC028 | 0.36 | 2.1 | NA | NA |

*SNV calling, but not SCNA calling, was possible in these biopsies.
