# Supplementary material for: Clonal Evolutionary Analysis during HER2 Blockade in HER2-Positive Inflammatory Breast Cancer: A Phase II Open-Label Clinical Trial of Afatinib +/- Vinorelbine
Source: PLoS Med. 2016 Dec 6;13(12):e1002136. doi: 10.1371/journal.pmed.1002136 (PMC5140058; doi:10.1371/journal.pmed.1002136)
Supplement: S5 Table — (DOCX) [file pmed.1002136.s017.docx]

# S5 Table. Clinical data and tumour response by patient.

| **Patient** | **Prior trastuzumab treatment** | **Part A: Afatinib monotherapy (N=26)** | | | | **Part B: Afatinib plus vinorelbine (N=10)** | | **Whole study** | | | |
| --- | --- | --- | --- | --- | --- | --- | --- | --- | --- | --- | --- |
|  |  | **Best overall response*** | **Best overall response confirmed** | **PFS [days]** | **PFS Censored** | **Best overall response*** | **Best overall response confirmed** | **Total PFS [days]** | **Total PFS Censored** | **OS [days]** | **OS status** |
| IBC014 | No | PR | PR^§^ | 165 | Event | PD | PD | 214 | Event | 711 | Event |
| IBC021 | No | PR | PR^§^ | 451 | Censored | NA | NA | 451 | Censored | 725 | Censored |
| IBC024 | No | PR | PR^§^ | 386 | Event | NA | NA | 395 | Event | 395 | Event |
| IBC017 | No | PR | PR^§^ | 111 | Censored | NA | NA | 111 | Censored | 154 | Censored |
| IBC022 | No | PR | PR^§^ | 713 | Event | NA | NA | 713 | Event | 713 | Event |
| IBC005 | No | PR | PR^§^ | 389 | Event | NA | NA | 389 | Censored | 929 | Censored |
| IBC010 | No | PR | PR^§^ | 701 | Event | NA | NA | 701 | Censored | 831 | Censored |
| IBC009 | No | PR | PR^§^ | 202 | Event | NEV | NEV | 223 | Censored | 302 | Censored |
| IBC012 | No | SD | SD^§^ | 554 | Censored | NA | NA | 554 | Censored | 688 | Censored |
| IBC001 | Yes | SD | SD | 163 | Censored | SD | SD^ | 413 | Event | 904 | Censored |
| IBC015 | No | SD | SD | 151 | Event | SD | SD | 253 | Event | 652 | Event |
| IBC020 | No | PR | SD | 109 | Event | SD | SD | 165 | Censored | 402 | Event |
| IBC002 | No | PR | SD | 110 | Event | PR | SD | 239 | Event | 873 | Censored |
| IBC008 | No | PR | SD | 109 | Event | PD | PD | 166 | Event | 230 | Event |
| IBC013 | Yes | SD | SD | 91 | Event | NA | NA | 91 | Event | 91 | Event |
| IBC003 | No | SD | SD | 111 | Event | NA | NA | 111 | Censored | 841 | Censored |
| IBC011 | Yes | PD | PD | 29 | Event | PR | SD | 136 | Event | 709 | Censored |
| IBC016 | Yes | PD | PD | 55 | Event | PR | PR^ | 253 | Event | 350 | Event |
| IBC028 | No | PD | PD | 58 | Event | NA | NA | 58 | Censored | 432 | Censored |
| IBC007 | No | PD | PD | 57 | Event | NA | NA | 57 | Censored | 870 | Censored |
| IBC004 | No | PD | PD | 52 | Event | NA | NA | 159 | Event | 159 | Event |
| IBC026 | No | PD | PD | 58 | Event | NA | NA | 58 | Censored | 400 | Event |
| IBC029 | No | PD | PD | 55 | Event | NA | NA | 55 | Censored | 477 | Censored |
| IBC027 | Yes | PD | PD | 64 | Event | NA | NA | 64 | Censored | 448 | Censored |
| IBC025 | Yes | NEV | NEV | 1 | Censored | PD | PD | 69 | Event | 306 | Event |
| IBC006 | Yes | NEV | NEV | 1 | Censored | NA | NA | 1 | Censored | 52 | Censored |

PR, partial response; SD, stable disease; PD, progressive disease; NEV, not evaluated because no post-baseline assessment of response to therapy; NA, not applicable; *, regardless of confirmation; §, confirmed clinical benefit in Part A; ^, confirmed clinical benefit in Part B.
