# Supplementary material for: Clonal Evolutionary Analysis during HER2 Blockade in HER2-Positive Inflammatory Breast Cancer: A Phase II Open-Label Clinical Trial of Afatinib +/- Vinorelbine
Source: PLoS Med. 2016 Dec 6;13(12):e1002136. doi: 10.1371/journal.pmed.1002136 (PMC5140058; doi:10.1371/journal.pmed.1002136)
Supplement: S7 Table — (DOCX) [file pmed.1002136.s019.docx]

# S7 Table. *TP53* mutations identified in IBC patients.

| Tumour | Genome Change | Variant  Classification | Protein  Change |
| --- | --- | --- | --- |
| IBC024 | g.chr17:7577538C>A | Missense | p.R248L |
| IBC020 | g.chr17:7577538C>T | Missense | p.R248Q |
| IBC005 | g.chr17:7577538C>T | Missense | p.R248Q |
| IBC025 | g.chr17:7577539G>A | Missense | p.R248W |
| IBC006 | g.chr17:7577539G>A | Missense | p.R248W |
| IBC021 | g.chr17:7577556C>Ta | Missense | p.C242Y |
| IBC009 | g.chr17:7577505T>A | Missense | p.D259V |
| IBC004 | g.chr17:7579350A>C | Missense | p.F113V |
| IBC001 | g.chr17:7577548C>T | Missense | p.G245S |
| IBC017 | g.chr17:7574018G>A | Missense | p.R337C |
| IBC008 | g.chr17:7574004_7574014delGAACATCTCGA | Frameshift | p.FEMF338fs |
| IBC011 | g.chr17:7579432_7579433delAG | Frameshift | p.P85fs |
| IBC026 | g.chr17:7579315_7579315insGC | Frameshift | p.C124fs |
| IBC015 | g.chr17:7579470_7579470insCG | Frameshift | p.V73fs |
| IBC013 | g.chr17:7578239C>A | Nonsense | p.E204* |
| IBC016 | g.chr17:7577610T>C | Splice site | c.e7-2 |
| IBC010 | g.chr17:7579311C>A | Splice site | c.e4+1 |
| IBC007 | g.chr17:7579311C>T | Splice site | c.e4+1 |
| IBC028 | g.chr17:7578556T>C | Splice site | c.e5-2 |
