# Supplementary material for: Clonal Evolutionary Analysis during HER2 Blockade in HER2-Positive Inflammatory Breast Cancer: A Phase II Open-Label Clinical Trial of Afatinib +/- Vinorelbine
Source: PLoS Med. 2016 Dec 6;13(12):e1002136. doi: 10.1371/journal.pmed.1002136 (PMC5140058; doi:10.1371/journal.pmed.1002136)
Supplement: S9 Table — (DOCX) [file pmed.1002136.s021.docx]

# S9 Table. GISTIC peaks identified in IBC (n=20).

| SCNA | cytoband | q value | wide peak boundaries | Overlaps non-IBC |
| --- | --- | --- | --- | --- |
| amplification | 17q12 | 9.22E-13 | chr17:37319104-38145087 | Yes |
| amplification | 8q24.21 | 4.89E-03 | chr8:114111198-139889971 | Yes |
| amplification | 1q32.1 | 5.80E-02 | chr1:181752803-249250621 | Yes |
| amplification | 17q11.2 | 7.13E-02 | chr17:26092556-27904710 | Yes |
| amplification | 11q13.5 | 1.01E-01 | chr11:76257216-83962250 | No |
| amplification | 17q25.1 | 1.67E-01 | chr17:53076800-81195210 | Yes |
| deletion | 11p15.5 | 2.09E-02 | chr11:1-17408629 | Yes |
| deletion | 8p23.3 | 2.27E-03 | chr8:1-6873602 | Yes |
| deletion | 8p12 | 2.27E-03 | chr8:12878831-41519247 | Yes |
| deletion | 3p22.1 | 2.69E-02 | chr3:33195285-94225785 | Yes |
| deletion | 1p36.13 | 5.61E-02 | chr1:16380253-17084767 | Yes |
| deletion | 5q12.3 | 6.02E-02 | chr5:41158864-171481605 | Yes |
| deletion | 10q26.3 | 1.51E-01 | chr10:44340866-135534747 | Yes |
| deletion | 2q37.3 | 1.56E-01 | chr2:231738169-243199373 | Yes |
| deletion | 4q34.3 | 1.61E-01 | chr4:169928843-191154276 | Yes |
| deletion | 6q13 | 1.60E-01 | chr6:57512566-136594327 | Yes |
| deletion | 13q14.11 | 2.01E-01 | chr13:39262058-73301654 | Yes |
| deletion | 15q11.1 | 2.09E-01 | chr15:1-30008855 | Yes |
