# Supplementary material for: Clonal Evolutionary Analysis during HER2 Blockade in HER2-Positive Inflammatory Breast Cancer: A Phase II Open-Label Clinical Trial of Afatinib +/- Vinorelbine
Source: PLoS Med. 2016 Dec 6;13(12):e1002136. doi: 10.1371/journal.pmed.1002136 (PMC5140058; doi:10.1371/journal.pmed.1002136)
Supplement: S1 Text — (PDF) [file pmed.1002136.s024.pdf]

Contents

1. DESCRIPTION OF DESIGN AND TRIAL POPULATION 2
1.1 OVERALL TRIAL DESIGN AND PLAN 2
1.2 OBJECTIVES 3
1.3 SELECTION OF THE TRIAL POPULATION 4
2. TREATMENTS 13
2.1 TREATMENTS TO BE ADMINISTERED 13
2.2 CONCOMITANT THERAPY, RESTRICTIONS, AND RESCUE TREATMENT 17
2.3 TREATMENT COMPLIANCE 20
3. VARIABLES AND THEIR ASSESSMENT 22
3.1 EFFICACY - PHARMACODYNAMICS 22
3.2 SAFETY 24
3.3 BIOMARKERS AND THEIR ASSESSMENT 24
4. STATISTICAL METHODS AND DETERMINATION OF SAMPLE SIZE 27
4.1 STATISTICAL DESIGN - MODEL 27
4.2 NULL AND ALTERNATIVE HYPOTHESES 27
4.3 PLANNED ANALYSES 27
4.4 HANDLING OF MISSING DATA 30
4.5 RANDOMISATION 30
4.6 DETERMINATION OF SAMPLE SIZE 30
5. REFERENCES 32
5.1 PUBLISHED REFERENCES 32
5.2 UNPUBLISHED REFERENCES 32
5.3 POSTERS/ONLINE PUBLICATIONS 33
6. APPENDICES 33
6.1 APPENDIX 1 COCKCROFT-GAULT FORMULA 33
6.2 APPENDIX 2 ECOG SCALE 34
6.3 APPENDIX 3 TUMOUR RESPONSE ASSESSMENT ACCORDING TO RECIST 1.1 35
6.4 APPENDIX 4 - CYP3A4 LIST AND PGP LIST 35
6.5 APPENDIX 5 CLINICAL EVALUATION OF LIVER INJURY 38
7. DESCRIPTION OF GLOBAL AMENDMENTS 40

Protocol: An open label, phase II trial of afatinib with or without vinorelbine for the treatment of HER2-overexpressing Inflammatory Breast Cancer

1. DESCRIPTION OF DESIGN AND TRIAL POPULATION

1.1 OVERALL TRIAL DESIGN AND PLAN

This is an open label study designed to investigate the efficacy and safety of afatinib monotherapy and upon progression on monotherapy as a combination with weekly vinorelbine (only for patients commencing treatment with this combination prior to 03May2013 and deriving benefit in the opinion of the investigator) in patients with HER2-overexpressing locally advanced or metastatic inflammatory breast cancer. All patients will start on afatinib monotherapy. In case of progression of disease on monotherapy they will continue with combination therapy as described (only for patients commencing treatment with afatinib and vinorelbine prior to 03May2013 and deriving benefit in the opinion of the investigator) (also refer to Figure 1.1:1 below).

Figure 1.1:1 Schematic of Trial Design

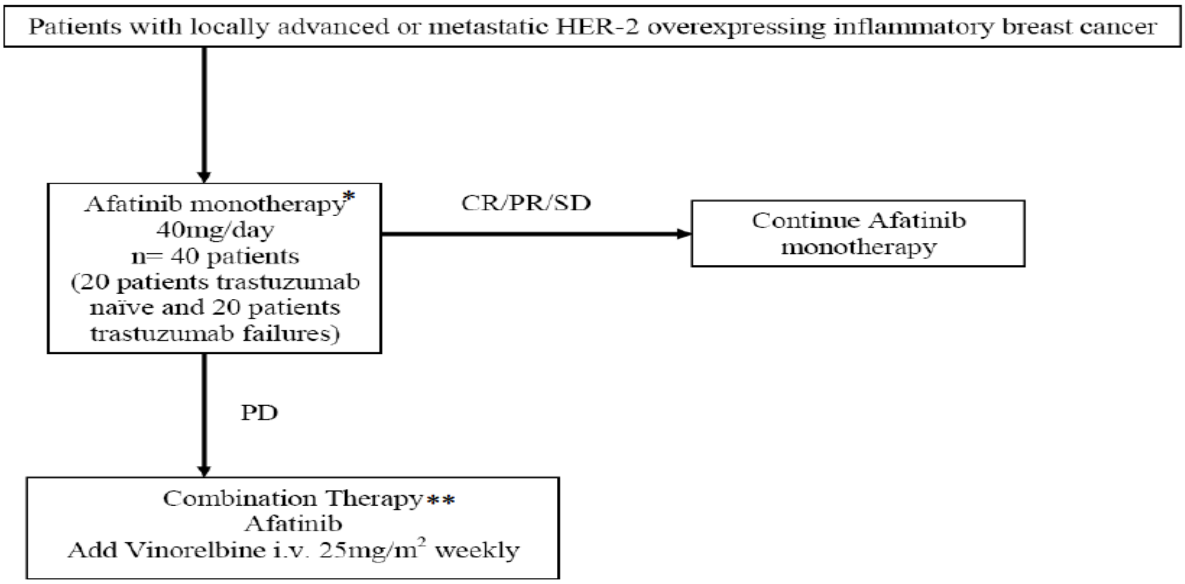

* patient recruitment will be stopped upon approval of Protocol Version 3.0, however patients who signed informed consent before that date may proceed in Part A only

All patients will initially undergo screening (up to 2 weeks) during which time their eligibility will be confirmed. Eligible patients will receive 40 mg/day afatinib monotherapy in 4-weekly treatment courses for the treatment of locally advanced or metastatic HER2-overexpressing inflammatory breast cancer, until disease progression (PD) assessed by RECIST 1.1 (see Flow Chart A and Figure 1.1:1 above). In the event of treatment-related adverse events, the dose of afatinib will be reduced according to Dose reduction scheme (Table 2.1.4:1).

Patients showing disease progression on afatinib monotherapy will then receive vinorelbine weekly, which will be added to afatinib (only for patients commencing treatment with this

combination prior to 03May2013 and deriving benefit in the opinion of the investigator) (see Flow Chart B and Figure 1.1:1 above).

The following measure has been proposed for ensuring appropriate and continuous treatment to the patients who were consented and treated in the study as of 03 May 2013:

- Part A: Patients in Part A (or in screening for Part A) should be informed that they will no longer be able to receive afatinib/vinorelbine in Part B upon progression. Patients should discuss with their investigator whether they want to continue afatinib monotherapy. If the patient and investigator agree, the patient will continue to receive treatment within Part A. All patients continuing to receive treatment within Part A at the time of approval of this amendment will need to be re-consented.

- Patients in screening for Part B should be informed that they will no longer be able to receive afatinib/vinorelbine in Part B and should cease afatinib monotherapy.

- Part B: Patients in Part B who are deriving benefit from treatment in the investigator's judgment, should discuss with their investigator whether they want to continue afatinib/vinorelbine. If the patient and investigator agree, the patient will continue to receive treatment within Part B. All patients continuing to receive treatment within Part B at the time of approval of this amendment will need to be re-consented.

Any patients not agreeing to continue treatment in Part A or Part B, or who were in screening for Part B as of 03May2013 will need to follow the clinical trial protocol for patients who permanently discontinue study treatment.

Any patients who have signed written informed consent after 03 May 2013 (until recruitment into Part A is stopped) may continue in the trial in Part A only.

All patients will attend an End of Treatment (EOT) Visit when they discontinue study treatment permanently. All patients will then have a follow-up visit at least 28 days after the EOT Visit. Following this, patients who still have not experienced disease progression and not started further treatment, will have further follow-up visits every 4 weeks until disease progression or start of further treatment. Once patients have progressed or started further treatment they will enter the observation period and will be contacted every 3 months until death or the study is completed to obtain vital status and follow up treatment information.

The primary analysis will occur at least 12 months after the last patient is entered or and at a time to be determined by the sponsor, when most or all patients have progressed or started further treatment. In any case patients still benefiting from treatment will continue to receive study medication.

Earlier studies with dual EGFR/HER2-inhibitors albeit reversible ones showed progression free survival (PFS) in the range of 15 weeks (R10-6339). It is therefore estimated patients will on average be on afatinib monotherapy for 12-16 weeks and on afatinib and vinorelbine combination therapy for 8-12 weeks.

1.2 OBJECTIVES

The primary objective of this trial is to investigate the efficacy and safety of afatinib alone and upon progression on afatinib monotherapy of afatinib in combination with vinorelbine (only for patients commencing treatment with this combination prior to 03 May 2013 and deriving benefit in the opinion of the investigator) in patients with locally advanced or metastatic HER2-overexpressing Inflammatory Breast Cancer. Patients must be HER2-positive by documented HER2-status in their medical files and must have biopsiable disease.

1.3 SELECTION OF THE TRIAL POPULATION

1.3.1 Main diagnosis for study entry

All patients that will be included into the trial must have been diagnosed with inflammatory breast cancer, have locally advanced and/or metastatic disease, have histologically confirmed breast cancer and must have proven HER2-overexpressing disease.

For Part A, patients must meet Inclusion Criteria 1 to 10 and not meet Exclusion Criteria 1-25.

For Part B, patients must meet Inclusion Criteria 1 to 11 and not meet Exclusion Criteria 2, 3, 4, 9, 11-26.

1.3.2 Inclusion criteria

1. Female patients ≥18 years.

2. Investigator-confirmed diagnosis of Inflammatory Breast Cancer defined as a clinico-pathologic entity that is characterized by diffuse erythema and oedema (peau d'orange). Pathological findings of dermal lymphatic emboli or palpable mass are supportive but not necessary for diagnosis.

3. Histologically confirmed breast cancer HER2-overexpressing disease (i.e. IHC 3+ or IHC 2+ with FISH/SISH positivity). (The central lab results of any analysis of archived tissue sample are not required to be available to confirm the eligibility of the patient to take part in the study).

4. Presence of locally advanced (in the opinion of the investigator) or metastatic disease.

5. For trastuzumab pre-treated patients, must have failed prior trastuzumab treatment in the investigator's opinion.

6. Must have biopsiable disease.

7. Must have disease that can be evaluated according to RECIST 1.1.

   NOTE: Patients with skin disease only may be included and their disease evaluated as non-target lesion(s) according to RECIST 1.1.

8. Eastern Cooperative Oncology Group (ECOG) score of 0, 1 or 2 (Ref: Appendix 2).

9. Life expectancy of at least six (6) months in the opinion of the investigator.

10. Written informed consent that is consistent with ICH-GCP guidelines and local legislation.

11. In addition for Part B (afatinib and vinorelbine combination therapy), patients must have progressed on afatinib monotherapy in Part A*.

    * No longer applicable after 03 May 2013

1.3.3 Exclusion criteria

1. Prior treatment with HER2-targeted small molecules or antibodies (other than trastuzumab which must have been given in the trastuzumab-failure study population).
2. Requirement for treatment with any of the prohibited concomitant medications listed in Section 2.2.2 (restrictions).
3. Must not have received prior vinorelbine in any setting
4. Known pre-existing interstitial lung disease (ILD).
5. Radiotherapy, chemotherapy, hormone therapy, immunotherapy, trastuzumab or surgery (other than biopsy) within 2 weeks prior to the first dose of afatinib in Part A. Patients must have recovered from previous lines of treatment in the investigator's opinion. Treatment with palliative radiotherapy (short course to non-target lesions) is allowed.
6. Active brain metastases (defined as stable for less than 4 weeks and/or symptomatic and/or requiring changes of treatment with anticonvulsants or steroids and/or leptomeningeal disease).
7. Any other current malignancy or malignancy diagnosed or relapsed within the past five (5) years (other than bilateral primary breast cancer, metastases to thecontralateral breast, non-melanomatous skin cancer and in situ cervical cancer).
8. Significant or recent acute gastrointestinal disorders with diarrhoea as a major symptom e.g. Crohn's disease, malabsorption or CTC grade ≥2 diarrhoea of any aetiology.
9. History or presence of clinically relevant cardiovascular abnormalities such as uncontrolled hypertension (in the investigators opinion), congestive heart failure NYHA classification of 3 or higher, unstable angina or poorly controlled arrhythmia (in the investigators opinion).
10. Myocardial infarction within 6 months prior to the first dose of afatinib.
11. Cardiac left ventricular function with resting ejection fraction of less than 50%.
12. Any other concomitant serious illness or organ system dysfunction which in the opinion of the investigator would either compromise patient safety or interfere with the evaluation of the safety of the test drug.
13. Absolute neutrophil count (ANC) < 1.5 x 10^9/L (1,500 cells/ mm^3).
14. Platelet count < 100 x 10^9/L (100,000 cells/ mm^3).
15. Calculated Creatinine clearance < 60 ml / min (using Cr^{51}-EDTA or Cockcroft-Gault formula – Ref: Appendix 1).
16. Bilirubin > 1.5 times upper limit of normal**.**

17. Aspartate amino transferase (AST) or alanine amino transferase (ALT) > 3 times ULN (if related to liver metastases > 5 times ULN).

18. Women of childbearing potential, unwilling to use a medically acceptable method of contraception during the trial.

    Acceptable methods of contraception include:

    - Surgical sterilisation (tubal ligation/hysterectomy)

    - Hormonal contraception (if applicable) with one barrier method

    - Double barrier method. Double barrier method of contraception is defined as two barrier methods used simultaneously each time the patient has intercourse.

    Accepted barrier methods include diaphragm, female condom, cervical cap, male condom and intra-uterine device (IUD) (female and male condom, diaphragm and cervical cap must all be used in conjunction with spermicidal jelly/cream). Partner vasectomy, natural "rhythm" and spermicidal jelly/cream are not acceptable methods of contraception.

    NOTE: Women of child bearing potential, must also continue to use effective contraception for at least 1 month after ceasing afatinib monotherapy and for at least 3 months after ceasing vinorelbine.

19. Pregnancy or breast-feeding (lactation).

20. Patients unable to comply with the protocol in the investigator's opinion.

21. Known Hepatitis B or C infection, or known HIV infection.

22. Known or suspected active drug or alcohol abuse in the opinion of the investigator.

23. Any contraindications for therapy with vinorelbine*.

24. Known hypersensitivity to afatinib or the excipients of any of the trial drugs (including vinorelbine).

25. Use of any investigational drug within 4 weeks of the first dose of afatinib and during the study.

26. <u>For Part B (afatinib and vinorelbine combination therapy),</u> prior treatment with HER2-targeted small molecules or antibodies other than <u>prior treatment with afatinib monotherapy in Part A</u> (and trastuzumab which must have been given in the trastuzumab-failure study population) *.

    * No longer applicable after 03May2013

If the screening blood results do not meet the eligibility criteria, they may be repeated within the protocol specified timeframe for the screening period.

1.3.4 Removal of patients from therapy or assessments

1.3.4.1 Removal of individual patients

A patient has to be withdrawn from study therapy in case any of the following applies:

1. The patient withdraws consent to further study treatment.

2. Documented progressive disease (see Appendix 3) on afatinib monotherapy (for patients in Part A on or after 03 May 2013) OR documented progressive disease on combination treatment of afatinib and vinorelbine, i.e. documented 2nd progression whilst on trial (for patients in Part B prior to 03 May 2013).
3. The patient is no longer able to receive any of the study treatments (e.g. adverse events, pregnancy, concomitant diagnoses, concomitant therapies or administrative reasons).
4. Significant deviation from the protocol or eligibility criteria. The decision to continue or withdraw treatment will be made after discussion between the sponsor and the investigator
5. Diagnosis of interstitial lung disease

6. If a patient experiences deterioration in left ventricular cardiac function (LVEF) to CTCAE Grade ≥3.
7. Further dose reductions considered necessary but not allowed according to the protocol (for exceptions, refer to Section 2.1.4)
8. The patient receives any of the prohibited medications listed in the current Summary of Product Characteristics (SPC) for vinorelbine (See Section 2.2)

All patients who are included into the trial (i.e. having given informed consent) will have their data entered into the trial database. This includes patients who are considered screen failures i.e. do not commence study drug.

1.3.4.2 Discontinuation of the trial by the sponsor

Boehringer Ingelheim reserves the right to discontinue the trial overall or at a particular trial site at any time for the following reasons:

1. Failure to meet expected enrolment goals overall or at a particular trial site,
2. Emergence of any efficacy/safety information that could significantly affect continuation of the trial,
3. Violation of GCP, the CTP, or the contract by a trial site or investigator, disturbing the appropriate conduct of the trial.

The investigator / the trial site will be reimbursed for reasonable expenses incurred in case of trial termination (except in case of the third reason).

Flow chart – Part A – Afatinib monotherapy

Follow this flow chart if patient continues on afatinib monotherapy	Screening*	Treatment Course 1**		Treatment Course 2 and subsequent courses**	ONLY PATIENTS NOT CONTINUING TO PART B		
					End of Treatment***	Follow-up****	Observation period
**Visit abbreviation**	**SV**	**C1V1**	**C1V2**	**C2V1 to CnV1**	**EOT #**	**FU1 to FUn #**	**OP1 - OPn #**
**Days**	**Day -14 to Day -1**	**Day 1**	**Day 15 (± 2 days)**	**Day 1 (± 2 days)**	**0-14 days after permanent discontinuation of afatinib monotherapy**	**FU1 - 28 days after EOT visit (± 2 days), FU2 to FUn – 28 days after FU1 (± 7 days)**	**Every 90 days after last follow-up visit (± 7 days)**
Informed Consent [1]	X						
Demographics	X						
Medical History	X						
Review of In-/Exclusion criteria	X	X					
Complete physical examination [2]	X				X		
Limited physical examination [2]		X		X		X	
Vital signs	X	X	X	X	X	X	
ECOG performance status	X	X	X	X	X	X	
12 Lead ECG [3]	X		X	X	X		
ECHO or MUGA scan [4]	X			X	X		
Safety lab [5]	X	X		X	X	X	
Urinalysis [5]	X				X		
Pregnancy test [6]	X				X		
Archived tumour sample	X [7]						
Fresh tumour biopsy [8]		X [9]			X [10]		
Blood samples for biomarker testing [8]		X [9]					
Tumour assessment	X	See schedule below [11]					
Adverse events	X	X	X	X	X	X [12]	X [12]
Concomitant medications	X	X	X	X	X	X	
Dispense trial drugs		X		X [13]			
Afatinib treatment		Continuous [14]					
Compliance check afatinib			X	X	X		
Termination of afatinib monotherapy					X		

Other anti-cancer therapy							X	X
Collection of vital status information [15]								X

Abbreviations for Visit types: SV = Screening Visit, C = Treatment course, V = Visit, EOT = End of Treatment Visit, FU = Follow-up Visit, OP = Observation Period

* HER2-retesting of archived biopsies will be performed by a central laboratory. The results of these analyses are not required to be available to confirm the eligibility of the patient to take part in the study. Inclusion into the trial will be based on documented HER2 over-expression status in the patient's files.
** All courses are 4 weeks in duration (28 ± 2 days). Patients may continue on treatment for unlimited courses, until the criteria for stopping medication are met.
*** If the decision to permanently discontinue afatinib is taken during a scheduled visit (and the patient will not continue to Part B), the EOT visit should be performed instead of the scheduled visit.
**** All patients not continuing into Part B, should have a follow-up visit 28 days after the EOT visit. Patients who have not progressed and not started further treatment should have further follow-up visits every 28 days until progression or start of further anti-cancer treatment.
#The EOT, FU visits and OP visits in Flow Chart A, are only applicable to patients who are permanently withdrawn from the study during afatinib monotherapy.

1. Written informed consent must be obtained before any protocol specific screening assessments are performed. Informed Consent must include consent to collection of demographic data and consent to obtaining and testing of archived tumour sample, fresh tumour biopsies and blood samples for biomarkers. Informed consent may be taken prior to Day -14 to allow for the shipment of the archived tumour sample to the central lab or FISH/SISH testing. However all other screening visit procedures (except tumour imaging) should not be performed prior to Day -14 (i.e. 14 days prior to the first dose of afatinib). Once all required approvals for Protocol Version 3.0 are obtained by the site, written informed consent of new patients must be stopped. Any patient who signed written informed consent before all required approvals for Protocol Version 3.0 were obtained by the site may continue in the trial.
2. Includes height (at screening only) and weight.
3. A 12-lead resting ECG will be performed at Screening, on Day 15 of Course 1, and then on Day 1 of every third course (Day 1 of Course 4, 7, 10 etc.), and at EOT (if not performed in the previous 12 weeks). In patients with skin lesions not tolerating thoracic electrodes, 3 lead Goldberger plus Einthoven ECG leads will be acceptable).
4. ECHO or MUGA will be performed at Screening, and then on Day 1 of every third course (Day 1 of Course 4, 7, 10 etc.), and at EOT (if not performed in the previous 12 weeks).
5. Safety laboratory tests (includes haematology and serum biochemistry) and Urinalysis performed within 3 days prior to the visit (except SV) are acceptable. If SV safety labs are taken within 3 days of C1V1 then safety labs do not have to be taken again at C1V1.
6. Pregnancy test (via blood preferred, urine only as back-up) is only to be performed if patient is of child bearing potential.
7. Archival tumour sample should be shipped to the central laboratory during screening. The following tests will be done by the central laboratory: HER2 IHC/FISH testing, hormone receptor (oestrogen and progesterone) IHC testing and EGFR IHC testing.
8. Visits should be scheduled Mon-Wed to allow for the samples to be collected and shipped to the central lab on the same day.
9. Must be taken prior to the first dose of afatinib. May be taken up to 2 days before C1V1.
10. Only in patients with disease progression.
11. Tumour assessments should be performed every 8 weeks from the first dose of afatinib. Tumour assessments for patients with radiologically measurable disease should include scans of the chest and abdomen and, if clinically indicated, imaging of any other known or suspected sites of disease using an appropriate method (CT scan, MRI). The same radiographic procedure must be used throughout the study. For patients with clinically evaluable skin disease, tumour assessments should also be performed every 8 weeks. If tumour imaging (CT/Bone Scan/MRI or other) has been performed as part of routine clinical practice within 28 days prior to the first dose of afatinib (Part A only), the imaging exams (CT/Bone Scan/MRI or other) do not need to be repeated. Unless the patient has known bone metastases, a bone scan should be performed during screening for all patients and then when clinically indicated. For patients with clinically known, stable brain metastases a brain MRI should be performed during screening and subsequently at every imaging time-point. In the event of early discontinuation or an interruption/delay of treatment, the tumour assessment schedule should not be changed. Tumour assessments may be performed up to 7 days prior to the scheduled tumour assessment date.
12. After FU1, only report AEs and SAEs which are considered study drug and/or trial related.
13. See Table 2.1.4:1 should patient require a dose reduction of afatinib.

14. If patient has shown disease progression (based on the clinical judgement of the investigator) on afatinib monotherapy (and vinorelbine will be added), then refer to Flow Chart B below. In addition afatinib monotherapy should be continued until start of combination treatment (Part B) and if necessary additional medication dispensed.
15. Collection of information on vital status, further anti-cancer treatment and (S)AEs considered related to study treatment or procedures. Information should be collected from the patient notes or by telephone contact with the patient. A formal study visit is not required.

Flow chart – Part B – Afatinib and vinorelbine combination treatment (only for patients commencing treatment with this combination prior to 03 May 2013)

Follow this flow chart if vinorelbine treatment is added (i.e. only patients with PD on afatinib monotherapy)	**Screening**	**Treatment Course 1[Δ]**		**Treatment Course 2 and subsequent treatment courses[Δ]**		**End of Treatment[ΔΔ]**	**Follow-up[ΔΔΔ]**	**Observation period**
**Visit abbreviation**	**vSV**	**vC1V1**	**vC1V2<br>vC1V3<br>vC1V4**	**vC2V1 to vCnV1**	**vC2V2 to vCnV2<br>vC2V3 to vCnV3<br>vC2V4 to vCnV4**	**EOT**	**FU1 to FUn**	**OP1 - OPn**
**Days**	**0-14 days after PD in Part A**	**Day 1 (0-14 days after PD in Part A)**	**Day 8<br>Day 15<br>Day 22<br>(± 2 days)**	**Day 1 (± 2 days)**	**Day 8<br>Day 15<br>Day 22<br>(± 2 days)**	**0-14 days after permanent discontinuation of Afatinib and Vinorelbine**	**FU1 - 28 days after EOT visit (± 2 days), FU2 to FUn – 28 days after FU1 (± 7 days)**	**Every 90 days after last follow-up visit (± 7 days)**
Complete physical examination[1]	X					X		
Limited physical examination[1]				X			X	
Re-review of In-/Exclusion criteria (for Part B – afatinib + vinorelbine)	X	X						
Vital Signs	X	X		X		X	X	
ECOG performance status	X	X		X		X	X	
12 Lead ECG[2]	X			X		X		
ECHO or MUGA scan[3]	X			X		X		
Safety lab[4]	X	X		X		X	X	
Urinalysis[4]	X					X		
CBC assessment prior to administering vinorelbine		X	X	X	X			
Pregnancy test[5]	X					X		
Fresh tumour biopsy	X[6]							
Tumour assessment	X[7]		See schedule below[8]					
Adverse events	X	X	X	X	X	X	X[9]	X[9]
Concomitant medications	X	X	X	X	X	X	X	
Dispense Trial drugs		X		X[10]				
Afatinib treatment		Continuous						
Vinorelbine treatment		Weekly[11]						
Compliance check afatinib/vinorelbine				X		X		

Termination of active treatment						X		
Other anti-cancer therapy							X	X
Collection of vital status information [12]								X

Abbreviations for Visit types: SV = Screening Visit, vC = Course of combination treatment with afatinib + vinorelbine, V = Visit, EOT = End of Treatment Visit, FU = Follow-up Visit, OP = Observation Period

A All courses are 4 weeks in duration (28 ± 2 days). Patients may continue on treatment for unlimited courses, until the criteria for stopping medication are met
AA If the decision to permanently discontinue afatinib and vinorelbine is taken during a scheduled visit, the EOT visit should be performed instead of the scheduled visit.
AAA All patients should have a follow-up visit 28 days after the EOT visit. Patients who have not progressed and not started further treatment should have further follow-up visits every 28 days until progression or start of further anti-cancer treatment.

1. Includes weight and body temperature (until the EOT).
2. A 12-lead resting ECG will be performed at Screening (unless performed in the last 14 days), and then on Day 1 of every third course (Day 1 of Course 4, 7, 10 etc.), and at EOT (if not performed in the previous 12 weeks). In patients with skin lesions not tolerating thoracic electrodes, 3 lead Goldberger plus Einthoven ECG leads will be acceptable.
3. ECHO or MUGA will be performed during screening (unless performed in the last 14 days) and then on Day 1 of every third course (Day 1 of Course 4, 7, 10 etc.), and at EOT (if not performed in the previous 12 weeks).
4. Safety laboratory tests (includes haematology and serum biochemistry) and Urinalysis performed within 3 days prior to the visit are acceptable. If vSV safety labs are taken within 3 days of vC1V1 then safety labs do not have to be taken again at vC1V1.
5. Pregnancy test (via blood preferred, urine only as back-up) is only to be performed if patient is of child bearing potential.
6. Must be taken prior to the first dose of vinorelbine.
7. A tumour assessment is not required to be repeated at this visit if it has already been performed at the point when PD was determined on afatinib monotherapy in Flow Chart A unless the first dose of vinorelbine treatment is delayed due to short course palliative radiotherapy in which case a tumour assessment should be performed within 14 days of the first dose of vinorelbine.
8. Tumour assessments should be performed every 8 weeks from the first dose of vinorelbine. Tumour assessments for patients with radiologically measurable disease should include scans of the chest and abdomen and, if clinically indicated, imaging of any other known or suspected sites of disease using an appropriate method (CT scan, MRI). The same radiographic procedure must be used throughout the study. For patients with clinically evaluable skin disease, tumour assessments should also be performed every 8 weeks. See Section 3.1.2 for further details.
   Bone scan should be performed when clinically indicated. For patients with clinically known, stable brain metastases a brain MRI should be performed at every imaging time-point. In the event of early discontinuation or an interruption/delay to treatment, the tumour assessment schedule should not be changed. Tumour assessments may be performed up to 7 days prior to the scheduled tumour assessment date.
9. After FU1, only report AEs and SAEs which are considered study drug and/or trial related.
10. See Table 2.1.4:1, should patient require a dose reduction of afatinib. Refer to Section 2.1.4 for should patient require vinorelbine to be withheld.
11. Vinorelbine 25 mg/m² i.v. administered weekly, only after progression on afatinib monotherapy. Each administration should be preceded by CBC assessment and measurement of weight and body temperature. Pre-medications should be administered as per SPC (see ISF).
12. Collection of information on vital status, further anti-cancer treatment and (S)AEs considered related to study treatment or procedures. Information should be collected from the patient notes or by telephone contact with the patient. A formal study visit is not required.

2. TREATMENTS

2.1 TREATMENTS TO BE ADMINISTERED

Patients will initially be treated with afatinib 40mg/day monotherapy until 1st disease progression on trial. Upon disease progression, patients will additionally receive vinorelbine at a weekly dose of 25 mg/m^2 (only for patients commencing treatment with this combination prior to 03May2013 and deriving benefit in the opinion of the investigator). The manufacturers for each of the products are listed in Section 2.1.1. Both study treatments will be supplied by Boehringer Ingelheim to the investigator for this study.

2.1.1 Identity of BI investigational product and comparator product

Substance (INN):	Afatinib (BIBW 2992)
Pharmaceutical form:	Film-coated tablets
Source:	Boehringer Ingelheim Pharma GmbH & Co. KG
Unit strengths:	40mg, 30mg and 20mg (the dose of afatinib in the film-coated tablets is related to the free base equivalent of afatinib)
Route of administration:	Oral (swallowed)
Posology:	40mg once daily
Duration of use:	Continuous daily dosing until disease progression, unacceptable adverse events or other reason necessitating withdrawal.

Substance (INN): Vinorelbine (Navelbine®)

Vinorelbine is a registered commercially available cytotoxic drug. Vinorelbine will be provided by the sponsor

Pharmaceutical form:	Concentrate solution for intravenous infusion
Source:	Pierre Fabre Pharma GmbH
Unit strengths:	50 mg/5mL and 10mg/1mL
Duration of use:	Weekly (Days 1, 8, 15 and 22) in a 4-weekly course. Patients will be eligible for repeated treatment courses until disease progression, unacceptable adverse events or other reason necessitating withdrawal.
Route of administration:	Intravenous, short infusion over approximately 10 minutes

Vinorelbine reconstitution: Vinorelbine must be diluted prior to administration in a 20-50mL volume of normal saline solution for injection or in 5 % glucose solution for injection

Posology:	25 mg/m^2 once weekly (only after Complete Blood Count (CBC) assessment)
Additional information:	Vinorelbine should be administered and the recommendations for supportive care followed according to the SPC.

2.1.2 Method of assigning patients to treatment group

All eligible patients will initially receive afatinib monotherapy on a continuous basis until disease progression, undue toxicity or withdrawal of consent. If disease progression occurs on afatinib monotherapy, then patients will receive additional vinorelbine treatment, whilst continuing on afatinib (only for patients commencing treatment with this combination prior to 03May2013 and deriving benefit in the opinion of the investigator). Patients will continue to receive afatinib in combination with vinorelbine until further disease progression, undue toxicity or withdrawal of consent.

Disease progression can occur at any time during the trial and the decision is always based on investigator judgement. It may be based on clinical assessment only (e.g. at the end of course 1) or on both clinical judgement and tumour imaging. In any case before combination treatment is started tumour imaging should take place. If there are no signs of PD, patients will continue on afatinib monotherapy.

2.1.3 Selection of doses in the trial

The Maximum Tolerated Dose (MTD) and recommended phase II dose for afatinib in combination with vinorelbine 25 mg/m^2 i.v. was determined at 40 mg in a continuous regimen.

The starting dose of afatinib in this trial was determined at 40 mg/day for monotherapy. 30mg and 20mg tablets of afatinib are available in case a patient requires dose reduction. See Section 2.1.4 below. (U03-3218, P11-07412)

2.1.4 Drug assignment and administration of doses for each patient

For administrative purposes, treatment will be divided into treatment courses, which are each 4 weeks (28 ± 2 days) in duration. This applies to both Part A (afatinib monotherapy) and Part B (afatinib and vinorelbine combination therapy).

Afatinib

Patients will take a single oral dose of 40 mg afatinib each day in Part A and Part B unless dose reduction is required. Once a dose is reduced it cannot be increased. Upon progression on afatinib monotherapy in Part A, if it is planned the patient will continue in the study and receive additional treatment with vinorelbine, then afatinib monotherapy should be continued.

Afatinib should be taken at the same time each day at least one hour before food intake and at least three hours after food intake. The time between the first dose of afatinib and the second dose of afatinib may be less than twenty-four hours but should be at least twelve hours. The tablet should be swallowed with a glass of water. Afatinib tablets are film-coated and therefore should not be chewed or crushed, but may be administered via G-tube after dispersing the afatinib tablets according to the following procedure: Place the tablet into a glass containing 50mL isotonic sodium chloride solution. Stir until the tablet is broken up into very fine particles (about 15 minutes). Drink the suspension immediately or administer via a gastric tube. Rinse the glass with another 50mL of isotonic sodium chloride solution

and drink or administer the supplementary solution via the gastric-tube again (to pick up any drug remaining in the glass/gastric-tube). Administration of afatinib via G-tube has to be recorded in the eCRF.

Missed doses of afatinib can be made up if taken up to +6 hours of the regularly scheduled dosing time. Otherwise, the dose should be withheld and patients should take the next scheduled dose at the usual time. Patients with emesis should not take a replacement dose.

In the event of treatment-related toxicities, the treatment with afatinib should be handled according to the schedule in Table 2.1.4:1.

Table 2.1.4:1 Afatinib Dose Reduction Scheme

AE type and grade	Action	Dose reduction scheme
Events related to study drug;<br>• Any **drug related** AE CTCAE Grade ≥3.<br>• CTCAE Grade ≥2 diarrhoea persisting for 2 or more consecutive days (48 hours) despite adequate anti-diarrhoeal medication/hydration.<br>• CTCAE Grade ≥2 nausea and/or vomiting persisting for 7 or more consecutive days despite anti-emetic treatment/ hydration.<br>• CTCAE Grade ≥2 worsening of renal function as measured by serum creatinine, newly developed proteinuria, or newly developed decrease in glomerular filtration rate of more than 50% from baseline.	Pause treatment with afatinib until patient has recovered to CTCAE Grade ≤ 1 or baseline[1]. Resume treatment at reduced dose according to schedule opposite.<br><br>If patient has not recovered to CTCAE Grade ≤ 1 or baseline[1] within 14 days study treatment should be permanently discontinued[2].	If patient was receiving 40mg, resume treatment at a dose of 30mg.<br><br>If patient was receiving 30mg, resume treatment at a dose of 20mg.<br><br>If patient was receiving 20mg, discontinue afatinib.

1 Baseline is defined as the CTCAE grade until start of treatment

2 In the event that the patient is deriving obvious clinical benefit in the opinion of the investigator, but has not recovered within 14 days, the further treatment of the patient will be decided by the investigator in agreement with the Trial Clinical Monitor.

Dose reduction should always follow a treatment pause. In the event of a treatment pause, subsequent visits/courses should not be delayed.

Patients will discontinue treatment if they experience deterioration in left ventricular cardiac function (LVEF) to CTCAE Grade ≥3.

In the event of a prolonged (≥7 consecutive days) Grade 2 drug-related event not listed in Table 2.1.4:1, which is poorly tolerated by the patient, the investigator may choose to pause the medication for up to 14 days to allow the patient to recover followed by a dose reduction according to the schedule in Table 2.1.4:1.

In the event of any unrelated adverse events or unrelated serious adverse events, the investigator may choose to pause the medication for up to 7 days to allow the patient to

recover, but no dose reduction should occur. If the investigator chooses to pause the medication for more than 7 days and believes that the patient would derive clinical benefit from continuing medication, the decision to continue medication will be made by the investigator in agreement with the Trial Clinical Monitor.

Vinorelbine (only for patients commencing treatment with the combination prior to 03May2013 and deriving benefit in the opinion of the investigator)

In Part B, Vinorelbine 25mg/m^2 i.v. will be administered weekly at the investigator site and will be prepared and administered in accordance with the current SPC.

A CBC must be performed and assessed within 48 hours prior to each weekly dose of vinorelbine. Treatment with vinorelbine must be withheld if platelet count is $< 100 \times 10^9$/L (100,000 cells/ mm^3) or ANC is $< 1.5 \times 10^9$/L (1,500 cells/ mm^3). Afatinib therapy should continue unless one of the criteria noted above is met regarding afatinib dosing.

The patient must be given supportive care during vinorelbine therapy in accordance with the current SPC and institutional guidelines.

In the event of treatment related adverse events the treatment dose of vinorelbine therapy will be skipped in accordance with the guidance in the current SPC. The current SPC for vinorelbine is provided in the ISF.

In the event of a skipped vinorelbine infusion due to adverse events, all visits including tumour assessments should continue to be performed according to the original schedule.

In the event of adverse events or serious adverse events which are not related to treatment, the investigator may choose to skip the dose, but no dose reduction should occur. If the investigator chooses to skip the dose the reason will need to be documented in the eCRF. Treatment will be resumed at the regular schedule thereafter.

Dose reduction for vinorelbine

Doses should continue to be skipped if vinorelbine is not tolerated and the investigator should decide if the patient should be permanently discontinued. Growth factor support will be allowed, following ASCO Guidelines (R09-0871).

In general, no dose adaptations for liver metastases or renal insufficiency are needed. In case of myelotoxicity, administration of subsequent doses will be skipped until recovery, i.e. platelet count $\geq 100 \times 10^9$/L (100,000 cells/ mm^3) and ANC $\geq 1.5 \times 10^9$/L (1,500 cells/ mm^3). Treatment should be continued within the regular schedule.

In cases of severe hepatic impairment a dose reduction of vinorelbine to 20 mg/m^2 is recommended per SPC and may be applied after causes for severe hepatic impairment on trial have been discussed between the sponsor and the investigator.

In the event that the patient is deriving obvious clinical benefit but a dose reduction of vinorelbine is deemed necessary in the opinion of the investigator, treatment continuation with vinorelbine at 20 mg/m^2 per week will be decided in agreement between the sponsor and the investigator.

2.2 CONCOMITANT THERAPY, RESTRICTIONS, AND RESCUE TREATMENT

2.2.1 Rescue medication, emergency procedures, and additional treatment(s)

Symptomatic treatments of tumour-associated symptoms are allowed including the use of corticosteroids and bisphosphonates. Concomitant medications, or therapy to provide adequate care, may be given as clinically necessary. Restrictions in Section 2.2.2 apply.

All concomitant (non-oncological) medications which are taken between trial informed consent and follow-up visit 1 should be recorded in the e-CRF (except for vitamins, appetisers or nutrient supplements which do not need to be recorded). After follow-up visit 1 only report concomitant medications given for AEs and SAEs considered study drug and/or trial related as well as further anti-cancer treatment. If patients receive parenteral nutrition during the trial, the components need not be specified in detail, it should just be indicated as "parenteral nutrition". If a patient requires anaesthesia, it will be sufficient to indicate "anaesthesia" without specifying the details.

For screen failures, only concomitant medications given for trial related adverse events will be collected.

Careful assessment of all patients with an acute onset and/or unexplained worsening of pulmonary symptoms (dyspnoea, cough, fever) should be performed to exclude ILD. Study drugs should be interrupted pending investigation of these symptoms. If interstitial lung disease is diagnosed, study drug must be permanently discontinued and appropriate treatment instituted as necessary.

Patients who present with symptoms of keratitis, such as acute or worsening eye inflammation, lacrimation, light sensitivity, blurred vision, eye pain and/or red eye should be referred promptly to an ophthalmic specialist. If a diagnosis of ulcerative keratitis is confirmed, treatment with afatinib should be interrupted or discontinued. If keratitis is diagnosed, the benefits and risks of continuing treatment with afatinib should be carefully considered. Afatinib should be used with caution in patients with a history of keratitis, ulcerative keratitis or severe dry eye. Contact lens use is a risk factor for keratitis and ulceration.

Rescue medications to reverse the actions of afatinib or vinorelbine are not available. There are no specific antidotes for overdosage with afatinib or vinorelbine. Please refer to the IB (U03-3218) for afatinib and the current SPC for vinorelbine (filed in the ISF) for further information. Side effects of trial medications should be treated symptomatically. Growth factor support, if required, will be used following ASCO Guidelines (R09-0871).

The current version of the investigator brochure lists the AEs expected with afatinib (U03-3218). Always refer to the most recent IB. Suggested treatments for diarrhoea, nausea, vomiting and rash/acne are described in Sections 2.2.2.3, 2.2.2.4 and 2.2.2.5.

There are no special emergency procedures to be followed.

2.2.2 Restrictions

2.2.2.1 Restrictions regarding concomitant treatment

Patients should not receive any additional experimental anti-cancer treatment, chemotherapy, radiotherapy, immunotherapy or maintenance therapy for metastatic breast cancer within 2 weeks prior to receiving the first dose of afatinib in Part A until the end of treatment visit. Treatment with palliative radiotherapy (short course to non-target lesions) is allowed.

For Afatinib: Afatinib is a substrate of the P-gp transporter. Caution should be exercised when combining afatinib with P-gp modulators. For a list of potent P-gp inhibitors and inducers (see Appendix 4)

For vinorelbine: Patients should not receive any of the prohibited medications as listed in the current SPC for vinorelbine (see ISF). Special care should be taken when administered with inducers or inhibitors of CYP 3A4 (see Appendix 4)

2.2.2.2 Restrictions on diet and life style

For Afatinib:

In the event of diarrhoea patients should be advised to avoid lactose-containing products or any foods known to aggravate diarrhoea.

To prevent skin related adverse events: During the treatment period, it is recommended to proactively avoid intense irradiation with UV light, e.g. sunbathing or visiting a solarium and strict sun protection should be used. In case of sun exposure a sunscreen of Sun Protection Factor 15 (SPF 15) or higher, preferably containing zinc oxide should be used. A thick, alcohol-free emollient cream should be used. Harsh detergents should be avoided.

For vinorelbine:

Refer to the current SPC (see ISF) for any restrictions on diet and lifestyle.

2.2.2.3 Management of diarrhoea following treatment with Afatinib

Close monitoring and proactive management of diarrhoea is essential for successful treatment of patients with afatinib. Early and appropriate intervention can prevent the development of more severe diarrhoea. In most cases, loperamide controls diarrhoea caused by afatinib. Loperamide should be available at the start of therapy and kept with the patient at all times; it is therefore advisable that patients be given a prescription at the time of initiating treatment with afatinib. Loperamide is considered a Non Investigational Medicinal Product (NIMP). For loperamide use, please refer to the current SPC.

The recommendations for management are as follows:

- If any diarrhoea is experienced (CTCAE Grade 1), two 2 mg loperamide tablets should be taken immediately, followed by one 2 mg tablet with every loose bowel movement, up to a maximum daily dose of 8 tablets (16 mg).
- Oral hydration is essential regardless of severity; appropriate rehydration (1.5 L/m^2/day plus equivalent of actual fluid loss) and electrolyte replacement has to be ensured in the event of CTCAE Grade 2 and Grade 3 adverse events.
- For CTCAE Grade 2 or 3 diarrhoea lasting ≥ 2 days (48 hours) despite adequate anti-diarrhoeal treatment, afatinib must be paused until recovery to CTCAE ≤ Grade

1. Upon recovery, afatinib should be resumed at a reduced dose according to the dose reduction scheme outlined in Table 2.1.4:1.

The occurrence of diarrhoea and the outcome of treatment will be recorded in the AE section of the eCRF. If the event is serious an SAE form should be completed and immediately faxed to the sponsor.

A patient diary may also be used during the study to collect information regarding a patient's bowel movements.

If despite optimal supportive care and a treatment pause, diarrhoea does not resolve to CTC Grade ≤1 within 14 days, the patient must not receive any further afatinib treatment. Restrictions given in Table 2.1.4:1, footnote 2 may apply.

2.2.2.4 Management of nausea and vomiting following treatment with Afatinib

Nausea and vomiting may significantly affect patients' adherence to the treatment and their quality of life. In order to reduce the occurrence and the intensity of emesis, the patients should be treated according to the recommendation given in Table 2.2.2.4:1.

Table 2.2.2.4:1 Management of nausea and vomiting

**CTCAE Grade**	**Antiemetic treatment**
Nausea = grade 0<br>and<br>Vomiting = grade 0	No antiemetic prophylactic treatment
Nausea = grade 1<br>and<br>Vomiting = grade 0	Antiemetic treatment if deemed necessary by the investigator
Nausea = grade 2<br>and<br>Vomiting = grade 0<br><br>Nausea = grade 0, 1 or 2<br>and<br>Vomiting = grade 1 or 2	Antiemetic treatment[1]<br><br>Pause afatinib treatment if grade 2 vomiting or grade 2 nausea persist for 7 or more consecutive days despite optimal supportive care. Resume treatment when CTCAE grade < 1.
Vomiting ? grade 3<br>or<br>Nausea ? grade 3	Antiemetic treatment[1]<br><br>Pause afatinib treatment until return to CTCAE grade < 1 or baseline[2].

1 Antiemetic treatment should follow the recommendations given in the Consensus Statement of the Antiemetic Subcommittee of the Multinational Association of Supportive Care in cancer (MASCC): Prevention of chemotherapy- and radiotherapy-induced emesis: Results of the Perugia Consensus Conference (R06-0986).
2 Baseline is defined as the CTCAE grade at the start of treatment.

After a treatment pause the dose of afatinib should be reduced according to the dose reduction scheme in Table 2.1.4:1.

The occurrence of nausea and/or vomiting and the outcome of treatment will be recorded in the AE section of the eCRF. If the event is serious an SAE form should be completed and immediately faxed to the sponsor.

In case of nausea and/or vomiting CTCAE grade 2, appropriate hydration (1.5 L/m^2/day plus hydration deficit) must be ensured.

2.2.2.5 Management of rash following treatment with Afatinib

A proactive and early approach to management of rash is crucial. Rash can be managed by a variety of treatment options to relieve symptoms and reduce the rash.

The recommendations for management are as follows:

- General/Prevention: see Section 2.2.2.2
- CTCAE Grade 1 rash: mild rash may not need treatment. However, if treatment is considered necessary, topical hydrocortisone (1% or 2.5%) cream and/or clindamycin 1% gel can be used.
- CTCAE Grade 2 rash: relief from major symptoms caused by CTCAE Grade 2 skin-related adverse events should be achieved by a combination of local and systemic therapies including:

  1) Systemic antibiotics (e.g. doxycycline or minocycline etc.).

  2) Topical treatment (e.g. hydrocortisone 2.5% cream, clindamycin 1% gel, pimecrolimus 1% cream).

  And / or

  1) Antihistamines (e.g. diphenhydramine, etc.)

  2) Oral corticosteroid (low dose and short term i.e. <10 days treatment) may be added at investigator's discretion.

  Systemic and topical treatment should be initiated at the start of CTCAE Grade 2 rash and continued until improvement or resolution to CTCAE Grade ≤1. If grade 2 rash persists for ≥ 7 days despite treatment and is poorly tolerated by the patient, the investigator may choose to pause treatment for up to 14 days followed by a reduction in the dose of afatinib according to the dose reduction scheme in Table 2.1.4:1

- CTCAE Grade 3 (or greater) rash: may be treated in a manner similar to CTCAE Grade 2 rash. In the event of CTCAE Grade ≥3 rash, treatment with afatinib should be paused until recovery to CTCAE Grade ≤1. Treatment should be resumed at a reduced dose (see Table 2.1.4:1). If CTCAE Grade ≥3 rash does not resolve to CTCAE Grade ≤1 within 14 days of stopping afatinib treatment and despite optimal supportive care, the patient should not receive any further treatment with afatinib. Restrictions given in Table 2.1.4:1, footnote 2 may apply.

The occurrence of rash and the outcome of treatment will be recorded in the AE section of the eCRF. If the event is serious an SAE form should be completed and immediately faxed to the sponsor.

2.3 TREATMENT COMPLIANCE

Study medications will be given in accordance with the protocol and under the instruction of the site investigator.

Afatinib

Patients treated with afatinib should take their first dose at the trial site. Subsequent doses should then be taken at home. A compliance check of trial medication should be performed on Day 15 of course 1 (Part A only) and day 1 of each subsequent course to ensure that the medication is being taken correctly. The patient should bring all remaining medication to the site and a compliance check should be performed. Discrepancies between the number of tablets remaining and the calculated number of tablets the patient should have taken should be documented and explained. Any significant compliance issues (<80% or >120%) should be discussed between the investigator and the sponsor. At the end of each course any remaining medication should be collected. If the patient is eligible for a further course of treatment a new bottle should be dispensed.

Patients experiencing emesis should not take a replacement dose. Afatinib should not be taken more than once a day under any circumstances.

Vinorelbine (only for patients commencing treatment with the combination prior to 03May2013 and deriving benefit in the opinion of the investigator)

Vinorelbine will be administered at the trial site under the supervision of the investigator and in accordance with the current SPC. In the event that the patient does not receive the full dose of vinorelbine or a dose is withheld this should be documented and a reason given.

3. VARIABLES AND THEIR ASSESSMENT

3.1 EFFICACY - PHARMACODYNAMICS

3.1.1 Endpoints of efficacy

3.1.1.1 Primary endpoint

The primary endpoint of this study, defined separately for afatinib monotherapy and combination therapy with vinorelbine, is:

- Clinical Benefit (CB) assessed by Stable Disease (SD) for at least 6 months, Partial Response (PR) or Complete Response (CR) using the Response Evaluation Criteria in Solid Tumours (RECIST 1.1).

3.1.1.2 Secondary endpoints

The secondary endpoints for this study, defined separately for afatinib monotherapy and combination therapy with vinorelbine, are:

- Objective Response (OR) assessed by CR and PR (RECIST 1.1)
- Duration of objective response, defined as the time from first objective response to the time of progression or death.
- Progression-Free Survival (PFS) will be defined for three time intervals: time from the date of the start of monotherapy to the date of 1st disease progression; time from the date of the start of combination therapy to the date of 2nd disease progression and the time from the date of the start of monotherapy to the date of 2nd disease progression. In each case, the date of death will be used if a patient dies without having progressed. The analysis will be based upon the evaluation of tumour imaging performed by the investigator using RECIST Version 1.1.

3.1.1.3 Other Endpoints

- Overall survival (OS)
- Best overall response during each treatment period according to RECIST 1.1
- Incidence of brain metastases
- ECOG performance status.
- Percentage change from baseline in tumour size

3.1.2 Assessment of efficacy

Efficacy will be evaluated according to RECIST 1.1 (R09-0262). Complete Response (CR), Partial Response (PR), Stable Disease (SD), Progressive Disease (PD) or Not Evaluable (NE) will be assessed by the investigator or authorised designee (Ref: Appendix 3 for RECIST 1.1 criteria).

Every effort should be made to objectively evaluate tumour response and confirm this using RECIST 1.1 for all patients who enter into the trial, including those who discontinue prematurely. (Ref: Appendix 3 for RECIST 1.1 criteria)

One to five target lesions (not exceeding two lesions per organ) should be identified at screening. Individual lesions detected at screening will be numbered and recorded in the eCRF. The size of the target lesions will be recorded in millimetres.

Tumour assessments should include scans of the chest and abdomen in patients with radiologically measurable disease and evaluation of clinical lesions in patients with skin disease. If clinically indicated, imaging of any other known or suspected sites of disease (e.g. breast, pelvis, brain, bone etc) using an appropriate method (CT scan, MRI) should be performed. The same radiographic procedure must be used throughout the study (except if contrast media is medically contra-indicated at any time during the study in which case MRI scans can be substituted for CT scans). Assessment will be performed at screening and then every 8 weeks after the first dose of afatinib. If tumour imaging (CT/Bone Scan/MRI or other) has been performed as part of routine clinical practice within 28 days prior to the first dose of afatinib (Part A only), the imaging exams (CT/Bone Scan/MRI or other) do not need to be repeated. In the event of early discontinuation or an interruption/delay to treatment, the tumour assessment schedule should not be changed.

Target lesions should be selected based on their size (those with the longest diameter) and suitability for accurate repeated measurements. All other lesions should be identified as non-target lesions and will be recorded at baseline. The non-target lesions will be followed during the patient's participation and will be taken into consideration when determining the patient's response.

Clinical lesions will only be considered measurable when they are superficial and ≥10mm diameter as assessed using calipers. For the case of skin lesions, documentation by colour photography including a ruler to estimate the size of the lesion is suggested. When lesions can be evaluated by both clinical exam and imaging, imaging evaluation should be undertaken (as per RECIST 1.1).

Unless the patient has known bone metastases a bone scan should be performed at screening (Part A only) for all patients. During study treatment, bone scans should be performed when clinically indicated, including in the case of suspected new bone metastases.

For patients with clinically known, stable brain metastases a brain MRI (T1-weighted gadolinium enhanced MRI) of the respective lesion(s) should be performed during screening (Part A only) and subsequently at every imaging time-point.

Correlative imaging (e.g. mammography, x-ray etc) of any site should be performed when clinically indicated.

If a patient has suspected disease progression earlier than Treatment Course 2 (C2V1) whilst on afatinib monotherapy, imaging (as applicable) should be done to confirm disease progression.

For patients who prematurely discontinue study medication without disease progression, tumour assessments should continue to be performed every 8 weeks until disease progression or start of further treatment.

3.2 SAFETY

3.2.1 Endpoints of safety

Safety of afatinib and vinorelbine will be evaluated as indicated by intensity and incidence of adverse events, graded according to CTCAE Version 3.0 (R04-0474).

3.3 BIOMARKERS AND THEIR ASSESSMENT

All patients must have biopsiable disease so that fresh tumour biopsies can be taken for biomarker evaluation. This is in addition to blood samples which will also be taken for biomarker evaluation. Fresh tumour biopsies and blood samples for biomarker evaluation will be taken at the timepoints specified in Flow Chart A and Flow Chart B. Further details of the biomarker evaluations and sample collection and processing is specified in Section 3.3.1.

An archived tumour sample will be shipped to a central laboratory for biomarker evaluations to retrospectively confirm the patient's HER2/hormone receptor/EGFR status. The results of these tests are not required to confirm the patients' eligibility to take part in the study.

3.3.1 Biomarker testing of archived tumour, fresh tumour and blood samples

3.3.1.1 Archived tumour sample analyses

An archived tumour sample should be sent to the central laboratory for biomarker analysis as outlined below.

Formalin Fixed Paraffin Embedded (FFPE) tumour slides or block(s) from the primary tumour or metastasis should be shipped to the central laboratory during the screening period.

HER2 testing
Earlier studies showed that there are discrepancies of up to 23% when comparing HER2 IHC and FISH analyses between local and central labs (R09-6008; R09-6009; R09-6011). To ensure that patients who had received and failed prior HER2-targeted treatment were truly HER2 over-expressing when they received this, confirmatory HER2-tests will be performed on archival tissue by a central laboratory.

The following tests will be done by a central laboratory: HER2 IHC and reflex FISH testing using a Food and Drug Administration (FDA) approved kit.

The central lab results of the HER2 tests are not required for patient eligibility. Only in cases where a patients HER2 status is negative, will the results be returned to the investigator (via the local clinical monitor). The local clinical monitor should discuss with the investigator whether the patient will remain in the study.

Hormone receptor and EGFR testing
Hormone receptor (oestrogen and progesterone) and EGFR status will also be tested at the central laboratory. The results of the hormone receptor and EGFR status may be used in a sub-analysis at the completion of the study. The results of these analyses will not be sent to the investigational site.

The following tests will be done by a central laboratory: hormone receptor (oestrogen and progesterone) and EGFR IHC testing using FDA-approved kit(s).

All tests will be performed by an authorised central laboratory according to standard procedures. Detailed instructions for the processing and shipping of these samples will be provided in a Laboratory Manual in the ISF.

3.3.1.2 Next Generation Sequencing, Proteomic Analysis and other exploratory analyses

Next generation sequencing (via DNA and RNA analysis), including gene expression and copy number, protemoic analysis and other exploratory analyses will be used in the search for predictive markers of response and resistance to afatinib. These analyses are exploratory only and are not approved tests and therefore cannot be used to reliably inform patients regarding genotype or treatments. As such the results of the analyses will not be sent to patients or investigators. The data generated from these analyses are hypothesis generating only and will be used in conjunction with functional laboratory studies in the search for markers of afatinib response and resistance.

Next generation sequencing, protemoic analysis and other exploratory analyses will be carried out on the following samples (when collected):

- *Fresh tumour biopsy - Part A – C1V1*
- *Fresh tumour biopsy - Part A – Upon disease progression*
- *Blood sample - Part A – C1V1 (to allow comparison between tumour and normal tissue)*

All tests will be performed by an authorised central laboratory according to standard procedures. Detailed instructions for the collection, processing and shipping of these samples will be provided in a Laboratory Manual in the ISF.

3.3.1.3 Blood sample analyses

Blood samples for biomarker testing will only be taken at C1V1 (Part A) i.e. at the same time as the first fresh tumour biopsy. The following analyses will be performed:

- o Soluble EGFR expression by ELISA
- o Ligands: epiregulin and amphiregulin by ELISA
- o Experimental testing of Circulating Nucleic Acids – this may include testing for PIK3CA, RAS, AKT1-3, EGFR, HER2, BRAF, NF1 and PIK3R1. As medical knowledge in this field is constantly evolving, other relevant predictive markers of response and resistance to the study treatments may be explored if there is sufficient sample.

All tests will be performed by authorised central laboratories according to standard procedures. Detailed instructions for the collection, processing, storage and shipping of these samples will be provided in a Laboratory Manual in the ISF.

3.3.2 Methods of sample collection

All samples will be collected as described in the Flow Chart A and Flow Chart B. No deviation from the schedule should be made regardless of treatment interruptions or dose reductions.

The FFPE tumour slides (minimum of 12 slides or at least 1 block) from the primary tumour or metastasis should be provided and sent to the central laboratory for retesting to confirm the patient's HER2 and hormone receptor status.

For fresh tissue biopsies, standardised tissue collection protocols (see Laboratory Manual) must be strictly followed and only sites capable of following these collection protocols will be able to participate in this study. It is preferable that the first fresh tissue biopsy (at C1V1) be taken from the chest wall, however if this is not possible another metastatic site should be used. It is preferable that the second fresh tissue biopsy (upon progression in Part A) be taken from the site of progression, however if this is not possible a sample should be taken from the chest wall. Core needle biopsies (rather then punch biopsies) should be the method of choice for the collection of fresh tissue biopsies, however a punch biopsy may be used if a core needle biopsy cannot be taken. A 16 gauge needle should be used to collect the core needle biopsy. If this is not possible then the next largest gauge should be used and 2 cores should be taken.

A fresh tumour sample will be taken from excised tumour material for patients undergoing surgery during Part A.

Blood samples will be collected as per standard local procedures.

3.3.3 Analytical determinations

HER2 IHC/FISH, Hormone receptor IHC and EGFR IHC will be performed according to standard procedures by a central lab.

Next generation sequencing, proteomic analyses and other exploratory analyses will be performed according to standard procedures by a central lab.

Soluble EGFR, amphiregulin, epiregulin and circulating nucleic acids analyses will be performed according to standard procedures by a central lab

Details of all analyses will be provided in a Laboratory Manual in the ISF.

4. STATISTICAL METHODS AND DETERMINATION OF SAMPLE SIZE

4.1 STATISTICAL DESIGN - MODEL

The trial will be performed as a single arm open label study. Patients will receive afatinib monotherapy (Part A) until disease progression according to RECIST 1.1. Once disease progression is confirmed, patients will be treated with afatinib in combination with vinorelbine (Part B) (only for patients commencing treatment with the combination prior to 03May2013 and deriving benefit in the opinion of the investigator) until further disease progression according to RECIST 1.1. (See Figure 1.1:1)

Once approval for Protocol Version 3.0 is obtained by the site, further patients must not be consented at the site (see Section 1.3.4.2).

4.2 NULL AND ALTERNATIVE HYPOTHESES

All analyses in this study are descriptive and exploratory by nature. Any statistical tests are performed only to provide a statistical framework from which to view the results and to provide aid for planning further studies. No formal statistical inferences are foreseen.

4.3 PLANNED ANALYSES

Treated analysis sets (TRT) are defined for Parts A and B separately. The TRT for Part A comprises all patients who received at least one single dose of afatinib, and the TRT for Part B comprises all patients who received at least one dose each of afatinib and vinorelbine.

Separate baselines will be defined for Parts A and B and no pooling will be performed across the two parts of the study unless otherwise stated.

The important protocol violations will be described.

4.3.1 Primary analyses

Clinical Benefit (CB)

Patients whose best RECIST 1.1 assessment is stable disease (≥6 months), partial, or complete response will be considered to have achieved Clinical Benefit. Only confirmed responses will be considered.

The primary analysis will estimate the proportion of patients who achieve clinical benefit for Parts A and B separately (according to RECIST 1.1 criteria). The individual response criteria will be relative to the respective baselines in each part and the rates will be presented using the respective TRTs. An exact 95% Clopper-Pearson confidence interval will be calculated for the proportion of patients who achieve clinical benefit (R06-1080).

Each patient will be assigned to one of the following categories based upon their best response during treatment in each part, as determined according to RECIST 1.1.

- 1 = CR (complete response)

- 2 = PR (partial response)
- 3 = SD (stable disease)
- 4 = PD (progressive disease)
- 9 = NE (not evaluable)

The proportion of patients in each RECIST category will also be tabulated at each planned imaging time.

4.3.2 Secondary analyses

Objective response and best overall response will be presented for both confirmed and unconfirmed RECIST 1.1 results. None of the other secondary endpoints which use RECIST 1.1, require confirmation.

4.3.2.1 Secondary Endpoints

4.3.2.1.1 Objective Response (OR)

Objective response is defined on a patient level as a best response of CR or PR. Point estimates and exact confidence intervals will be calculated for the proportion of patients who achieve objective response. Results will be presented by patients with target lesions only, patients with non-target lesions only, patients with both and patients with either. Confirmed and unconfirmed responses will be presented separately.

4.3.2.1.2 Duration of objective response

Duration of objective response is measured from the time of first objective response to the time of progression or death (or date of censoring for PFS).

Time to objective response is defined as the number of days from the start of treatment to the first recorded objective response in each part.

Descriptive statistics will be produced for the duration of objective response for Parts A and B separately.

4.3.2.1.3 Progression-free survival

Disease progression will be evaluated according to the RECIST 1.1 criteria (R09-0262).

For patients with known date of progression (or death):

- PFS [days] = earlier of date of progression or death – date of first administration + 1.

Date of progression and date of first administration is referring to the respective part of the study (A or B).

PFS will also be defined for the following time interval:

- PFS [days] = earlier of date of death or second progression – date of first administration in Part A + 1.

PFS will be assessed based on the Kaplan-Meier method for each part separately. Point estimates together with confidence intervals (based on Greenwood's method) will be

provided for median PFS as well as for PFS rates at specified time points. Details of time points and censoring rules will be provided in the TSAP.

4.3.2.2 Other Endpoints

4.3.2.2.1 Overall Survival

For patients with known date of death (regardless of the cause of death):

Overall survival [days] = date of death – (date of start of Part A) + 1 For patients known to be alive by the end of trial or follow-up visit:

Overall survival (censored) [days] = last date when the patient is known to be alive – (date of start of Part A) + 1

Descriptive statistics will be produced for the overall survival.

4.3.2.2.2 Best overall response

Patients' best responses according to RECIST 1.1 during each treatment period, will be tabulated separately. Confirmed and unconfirmed responses will be presented separately.

Frequency tables will be presented for the best RECIST assessment achieved by each patient during each of the treatment periods in the order (from best to worst): complete response, partial response, stable disease, disease progression

4.3.2.2.3 Incidence of brain metastases

Number of patients with new brain metastases will be tabulated. If considered necessary, primary and secondary analyses for CBR as well as ORR will be repeated in the subgroup of patients with brain metastases (see Section 4.3.2.2.5).

4.3.2.2.4 ECOG performance status

ECOG performance status at baseline and at end of treatment will be tabulated. If deemed appropriate time to deterioration of ECOG performance status will be analysed where deterioration is any increase in ECOG performance status.

4.3.2.2.5 Percentage change from baseline in tumour size

Descriptive statistics will be presented for the percentage change from baseline in tumour size in both Part A and Part B of the study.

4.3.2.2.6 Subgroups

If considered necessary CBR and ORR will be evaluated for the following subgroups for Parts A and B separately:

- Hormone receptor [oestrogen, progesterone]

- EGFR status [positive, negative]

- New brain metastases [Yes, No]

- Target vs non-target lesions

- Prior trastuzumab therapy [Yes, No]

Other subgroups may be explored as appropriate and will be defined in the TSAP.

4.3.3 Safety analyses

Safety analyses will be performed for Parts A and B separately using the respective TRTs.

Adverse events as well as laboratory parameters will be graded according to CTCAE, Version 3.0 (R04-0474). Key safety measures will include:

- events leading to permanent dose reduction
- events leading to permanent treatment discontinuation
- the overall incidence and CTC criteria grade of adverse events, as well as relatedness of adverse events to treatment
- causes of death
- gastrointestinal events (diarrhoea, vomiting, nausea)
- skin disorders
- mucosal inflammation
- renal insufficiency
- elevated liver function tests,
- haematological abnormalities (anaemia, thrombocytopenia, neutropenia)
- CTCAE Grade 2 with increase by at least one CTCAE grade from baseline, for selected laboratory tests:
- (high values) INR, creatinine, AST, ALT, total bilirubin, alkaline phosphatase,
- (low values) haemoglobin, neutrophils, platelets, WBC, magnesium, potassium
- descriptive statistics for change from baseline for all quantitative laboratory tests

Additional, more in-depth analyses will be performed as needed. These analyses will examine the influence of extent of exposure and time to event onset.

Vital signs and Left ventricular ejection fraction will be listed and described descriptively.

4.3.4 Interim analyses

Not applicable to this protocol

4.4 HANDLING OF MISSING DATA

Patients will continue to be followed for progression after discontinuation of study treatment until death or until the study is closed by the sponsor.

4.5 RANDOMISATION

This is an open-label non-randomised trial without a control group. No randomisation is involved in this trial. Eligible patients will be sequentially entered into the trial.

4.6 DETERMINATION OF SAMPLE SIZE

A sample size of 40* has been selected for this study. With this number of patients, efficacy signals may be detected, however the study is not powered for this and these signals are not confirmatory. The table below illustrates various probabilities associated with different sample sizes assuming an underlying clinical benefit rate of 50%.

* Once approval for Protocol Version 3.0 is obtained by the site, further patients must not be consented at the site (see Section 1.3.4.2). As a result, less than 40 patients may be entered when recruitment is completed.

Table 4.6:1 Statistical power for an underlying clinical benefit rate of 50%

Number of patients	Observed number of patients with clinical benefit	Observed clinical benefit rate (R_0)	Probability of clinical benefit rate in excess of R_0, given true P=50%
30	16	53%	43%
30	20	67%	5%
30	24	80%	<1%
40	16	40%	92%
40	20	50%	56%
40	24	60%	13%
50	16	32%	>99%
50	20	40%	94%
50	24	48%	66%

Assuming an underlying clinical benefit rate of 50%, 40 patients would be expected to provide more than a 90% probability of observing a clinical benefit rate of at least 40% (i.e. 16 or more patients achieving clinical benefit). On the other hand, the expected probability of observing at least 16 patients with clinical benefit would be 43% among 30 patients, and in excess of 99% for 50 patients.

The primary analysis will occur at least 12 months after the last patient is entered and at a time to be determined by the sponsor, when most or all patients have progressed or started further treatment.

5. REFERENCES

5.1 PUBLISHED REFERENCES

R04-0474 Common terminology criteria for adverse events v3.0 (CTCAE) (publish date: December 12, 2003). http://ctep.cancer.gov/forms/CTCAEv3.pdf ; Cancer Therapy Evaluation Program, Common Terminology Criteria for Adverse Events, Version 3.0, DCTD, NCI, NIH, DHHS, March 31, 2003, Publish Date: December 12, 2003

R06-0986 Antiemetic Subcommittee of the Multinational Association of Supportive Care in Cancer (MASCC). Prevention of chemotherapy- and radiotherapy-induced emesis: results of the 2004 Perugia International Antiemetic Consensus Conference. Ann Oncol 2006;17(1):20-28

R06-1080 Clopper CJ, Pearson ES. The use of confidence or fiducial limits illustrated in the case of the binomial. Biometrika 1934;26:404-413.

R09-0262 Eisenhauer EA, Therasse P, Bogaerts J, Schwartz LH, Sargent D, Ford R, et al. New response evaluation criteria in solid tumours: revised RECIST guideline (version 1.1). Eur J Cancer 2009; 45:228-247

R09-0871 Smith TJ, et al. 2006 update of recommendations for the use of white blood cell growth factors: an evidence-based clinical practice guideline. J Clin Oncol 2006;24(19):3187-3205

R09-6008 Gancberg D, Di Leo A, Cardoso F, Rouas G, Pedrocchi M, Paesmans M, et al. Comparison of HER-2 status between primary breast cancer and corresponding distant metastatic sites. Annals of Oncology 2002; 13: 1036-1043

R09-6009 Perez EA, Suman VJ, Davidson NE, Martino S, Kaufmann PA, Lingle WL, et al. HER2 Testing by Local, Central, and Reference Laboratories in Specimens From the North Central Cancer Treatment Group N9831 Intergroup Adjuvant Trial. Journal of Clinical Oncology 2006; 24(19): 3032-3038

R09-6011 McCullough A, Dueck A, Chen B, Reinholz M, Wiktor A, Lingle W, et al. HER-2 central confirmatory testing using ASCO/CAP guidelines for trastuzumab/lapatanib trial MCCR RC0639. Journal of Clinical Oncology ASCO Annual Meeting Proceedings 2009; 27: No15S May 20 Supplement, 2009: e11527

R10-6339 Johnston S, Trudeau M, Kaufman B, Boussen H, Blackwell K, LoRusso P, et al. Phase II study of predictive biomarker profiles for response targeting human epidermal growth factor receptor 2 (HER-2) in advanced inflammatory breast cancer with lapatinib monotherapy. J Clin Oncol 2008;26(7):1066-1072

5.2 UNPUBLISHED REFERENCES

U03-3218 Afatinib (BIBW2992) Investigator's Brochure Version **13** dated **11 Jul 2012**

5.3 POSTERS/ONLINE PUBLICATIONS

P11-07412 Bahleda R, Soria J, Berge Y, Massard C, Wind S, Uttenreuther-Fischer MM, Fleischer F, De-Montserrat H, Solca F, Tschoepe I, Delord J. Phase I trial assessing safety and pharmacokinetics of afatinib (BIBW 2992) with intravenous weekly vinorelbine in advanced solid tumors. 47th Ann Mtg of the American Society of Clinical Oncology (ASCO), Chicago, 3 - 7 Jun 2011 J Clin Oncol 2011;29(15S)(Suppl) Abstr 2585

6. APPENDICES

6.1 APPENDIX 1 COCKCROFT-GAULT FORMULA

Estimated creatinine clearance rate (eCCR) using Cockcroft-Gault formula

$$eC_{CR} = \frac{\textbf{(140-Age)X Mass (in kilograms) X [0.85 } \textbf{\textit{if Female}}\textbf{]}}{\textbf{72 X Serum Creatinine (in mg/dL)}}$$

Or when serum creatinine is measured in µmol/L

$$eC_{CR} = \frac{\textbf{(140-Age)X Mass (in kilograms) X } \textbf{\textit{Constant}}}{\textbf{Serum Creatinine (in } \mu \textbf{mol/L)}}$$

Where *Constant* is *1.23* for men and *1.04* for women

6.2 APPENDIX 2 ECOG SCALE

**ECOG Performance Status Scale**	
Grade	Descriptions
0	Normal activity. Fully active, able to carry on all pre-disease performance without restriction.
1	Symptoms, but ambulatory. Restricted in physically strenuous activity, but ambulatory and able to carry out work of a light or sedentary nature (e.g., light housework, office work).
2	In bed <50% of the time. Ambulatory and capable of all self-care, but unable to carry out any work activities. Up and about more than 50% of waking hours.
3	In bed >50% of the time. Capable of only limited self-care, confined to bed or chair more than 50% of waking hours
4	100% bedridden. Completely disabled. Cannot carry on any self-care. Totally confined to bed or chair
5	Dead.

6.3 APPENDIX 3 TUMOUR RESPONSE ASSESSMENT ACCORDING TO RECIST 1.1

Response criteria for target lesions

1. Complete Response (CR):	Disappearance of all target lesions. Any pathological lymph nodes (whether target or non-target) must have a reduction in short axis to <10mm)
2. Partial Response (PR):	At least a 30% decrease in the sum of diameters of target lesions taking as reference the baseline sum diameters
3. Progression (PD):	At least a 20% increase in the sum of diameters of target lesions, taking as references the smallest sum on study (this includes the baseline sum if that is the smallest on study). In addition to the relative increase of 20%, the sum must also demonstrate an absolute increase of a least 5mm (note: the appearance of one or more new lesions is also considered progression).
4. Stable Disease (SD):	Neither sufficient shrinkage to qualify for PR nor sufficient increase to qualify for PD, taking as references the smallest sum diameters while on study

Response criteria for non-target lesions

1. Complete Response (CR):	Disappearance of all non-target lesions and normalization of tumour marker level. All lymph nodes must be non-pathological in size (<10mm short axis)
2. Non-CR/ Non-PD:	Persistence of one or more non-target lesion(s) or/and maintenance of tumour marker level above the normal limits.
3. Progression (PD):	Unequivocal progression of existing non-target lesions (Note: the appearance of one or more new lesions is also considered progression)

Time-point response

Target lesions	Non-Target lesions	New lesions	Overall response
CR	CR	No	CR
CR	Non-CR/Non-PD	No	PR
CR	Not evaluated	No	PR
PR	Non-PD or not all evaluated	No	PR
SD	Non-PD or not all evaluated	No	SD
Not all evaluated	Non-PD	No	NE
PD	Any	Yes or No	PD
Any	PD	Yes or No	PD
Any	Any	Yes	PD

6.4 APPENDIX 4 - CYP3A4 LIST AND PGP LIST

6.4.1. Summary of CYP 3A4 Substrates, Inhibitors, and Inducers

Chart of Human CYP-450 3A4 Isoenzyme Selective Substrate, Inhibitors, & Inducers

**Substrates**	**Inhibitors**	**Inducers**
acetaminophen, alfentanil, alprazolam, amiodarone, aminopyrine, amitriptyline, amlodipine, amprenavir, antipyrine, astemizole, atorvastatin, benzphetamine, budesonide, busulfan, cannabinoids, carbamazepine, celecoxib, cisapride, clarithromycin, clindamycin, clomipramine, clozapine, codeine, cortisol, cyclobenzaprine, cyclophosphamide, cyclosporin A, dapsone, delavirdine, dexamethasone, dextromethorphan, diazepam, digoxin, diltiazem, disopyramide, docetaxel, donepezil, doxorubicin, dronabinol, erythromycin, ethinylestradiol, ethosuximide, etopside, felodipine, fentanyl, fexofenadine, flutamide, granisetron, haloperidol, hydrocortisone, ifosfamide, imipramine, indinavir, isradipine, ketoconazole, lansoprazole, lidocaine, loratadine, losartan, lovastatin, methadone, mibefradil, miconazole, midazolam, navelbine, nefazodone, nelfinavir, nicardipine, nifediprine, nimodipine, nisoldipine, omeprazole, ondansetron, paclitaxel, pravastatin, prednisone, propafenone, quindine, quinine, retinoic acid, rifampin, ritonavir, ropivacaine, saquinavir, sertraline, sufentanil, tacrolimus, tamoxifen, temazepam, teniposide, terfenadine, testosterone, THC, theophylline, triazolam, troleandomycin, verapamil, vinblastine, vincristine, (R)warfarin	amiodarone, amprenavir, cannabinoids, cimetadine, clarithromycin, clotrimazole, cyclosporin, delavirdine, diltiazem, ethinylestradiol, erythromycin, fluconazole, fluoxetine, fluvoxamine, indinavir, intraconazole, ketoconazole, metronidazole, mibefradil, miconazole, nefazodone, nelfinavir, nicardipine, norfloxacin, propafol, quinine, ritonavir, saquinavir, sertraline, troleandomycin, verapamil, zafirlukast	carbamazepine, dexamethasone, ethosuximide, glutethimide, nevirapine, phenobarbital, phenytoin, primidone, rifabutin, rifampin, St. John’s Wort, sulfadimidine, sulfinpyrazone, troglitazone, troleandomycin

6.4.2. List of potent inhibitors and inducers of P-glycoprotein

Inhibitors	Inducers
Amiodarone	Carbamazepine
Azithromycin	Phenytoin
Captopril	Rifampicin
Carvedilol	St John's Wort
Clarithromycin	Phenobarbital Salt
Conivaptan	Tipranavir
Cyclosporine	Ritonavir
Diltiazem	
Dronedarone	
Erythromycin	
Felodipine	
Itraconazole	
Ketoconazole	
Lopinavir	
Nelfinavir	
Ritonavir	
Quinidine	
Ranolazine	
Saquinavir	
Tacrolimus	
Ticagrelor	
Verapamil	

As the information on potent inhibitors and inducers of P-glycoprotein may evolve, it is important for the investigator to assess the status of each concomittant therapies and in case of questions contact BI clinical monitor.

While caution needs to continue to be exercised in concomitant use of P-gp inhibitors/inducers with afatinib, based on new data from two drug-drug interaction (DDI) trials (U03-3218) investigating the effect of ritonavir and rifampicin, respectively, on 40 mg afatinib, their use in patients needing such therapies is no longer prohibited. It cannot be excluded that the plasma exposure to afatinib may increase under concomitant treatment with strong P-gp inhibitors. Conversely, strong P-gp inducers may decrease the plasma concentrations of afatinib. However, maximum observed effects are rather mild to moderate and could even be avoided for the potent inhibitor ritonavir when given simultaneously or 6 h after afatinib. Therefore caution has to be exercised when combining afatinib with potent P-gp modulators.

6.5 APPENDIX 5 CLINICAL EVALUATION OF LIVER INJURY

6.5.1 Procedures

Any elevation of ALT/AST and bilirubin qualifying as laboratory alert should be confirmed using the initial sample if possible.

If the alert is confirmed on initial sample, or it is not possible to repeat testing using initial sample, the following must be completed:

1) Evaluate patient within 48 hours and

2) Perform the following laboratory tests:

   1. Repeat of AST, ALT, bilirubin (with fractionation to total and direct)
   2. Haptoglobin
   3. Complete blood count and cell morphology
   4. Reticulocyte count
   5. Creatine Kinase (CK)
   6. Lactate dehydrogenase (LDH)
   7. Alkaline Phosphatase

The results of these laboratory tests must be reported to BI as soon as possible.

If the initial alert values (*ie* AST, ALT, and bilirubin) are confirmed on the second sample described as above, then an abdominal ultrasound or clinically appropriate alternate imaging (to rule out biliary tract, pancreatic or intrahepatic pathology, e.g. bile duct stones or neoplasm) must be completed within 48 hours.

The findings from the hepatic imaging (including comparison to prior imaging if available) must be made available as soon as possible as part of the adverse event reporting process. In the event the aetiology of the abnormal liver tests results is not identified based on the imaging (e.g. biliary tract, pancreatic or intrahepatic pathology), then the "DILI checklist" must be completed. Details of the "DILI checklist" are provided in the ISF. The following assessments need to be performed in order to complete the "DILI checklist" and any resulting diagnoses will be reported via the eCRF:

- obtain a detailed history of current symptoms and concurrent diagnoses and medical history according to the "DILI checklist" provided in the ISF;
- obtain history of concomitant drug use (including non-prescription medications, herbal and dietary supplement preparations), alcohol use, recreational drug use, and special diets according to the "DILI checklist" provided in the ISF;
- obtain a history of exposure to environmental chemical agents (consider home and work place exposure) according to the "DILI checklist" provided in the ISF;
- complete the following laboratory tests as detailed in the DILI checklist provided in the ISF:

– *Clinical chemistry*
alkaline phosphatase, cholinesterase (serum)*, albumin, PT or INR, CK, CK-MB, coeruloplasmin*, α-1 antitrypsin*, transferrin*, amylase, lipase, fasting glucose, cholesterol, triglycerides

– *Serology*
Hepatitis A (Anti-IgM, Anti-IgG), Hepatitis B (HbsAg, Anti-HBs, DNA), Hepatitis C (Anti-HCV, RNA if Anti-HCV positive), Hepatitis D (Anti-IgM, Anti-IgG), Hepatitis E (Anti-HEV, Anti-HEV IgM, RNA if Anti-HEV IgM positive), Anti-Smooth Muscle antibody (titer), Anti-nuclear antibody (titer), Anti-LKM (liver-kidney microsomes) antibody, Anti-mitochondrial antibody, Epstein Barr Virus (VCA IgG, VCA IgM), cytomegalovirus (IgG, IgM), herpes simplex virus (IgG, IgM)*, varicella (IgG, IgM)*, parvovirus (IgG, IgM)*

– *Hormones, tumour marker*
Thyroid-stimulating hormone (TSH)*

– *Haematology*
Thrombocytes, eosinophils

**If clinically indicated (e.g. immunocompromised patients)*

➢ Long term follow-up

– Initiate close observation of subjects by repeat testing of ALT, AST, and bilirubin (with fractionation to total and direct) at least weekly until the laboratory ALT and or AST abnormalities stabilize or return to normal, then according to the protocol. Depending on further laboratory changes, additional parameters identified e.g. by reflex testing will be followed up based on medical judgement and Good Clinical Practices (GCP).

Report any resulting diagnoses via the eCRF.

7. DESCRIPTION OF GLOBAL AMENDMENTS

The CTP was amended 3 times.

7.1 REASONS FOR AMENDMENT 1

Updated data from drug-drug interaction studies whereby the concomitant use of P-gp inducers/inhibitors with afatinib was no longer prohibited, however caution still needed to be exercised

Added statement on risk of Keratitis and ulcerative keratitis that had been reported following treatment with currently approved EGFR inhibitors for cancer, for which the European Medicines Agency (EMA) had requested a class labelling.

Further clarifications were provided.

7.2 REASONS FOR AMENDMENT 2

All relevant sections of the protocol updated to clarify that only patients who commenced treatment within Part B i.e. with afatinib and vinorelbine prior to 03May2013, and had benefit as assessed by the investigator at this time, were allowed to continue in Part B. All other patients (either new, in Part A, or in screening for Part B) could not start treatment within Part B.

A pre-defined benefit risk analysis had been performed by an independent Data Monitoring Committee (DMC) for the 1200.75 trial. The DMC evaluated the benefit/risk ratio of the afatinib/vinorelbine combination as unfavourable and recommended discontinuing recruitment into the 1200.75 trial.
The sponsor decided as a precautionary measure and in the absence of any unexpected safety signal in the 1200.89 trial, to stop the inclusion of new patients into Part B of the 1200.75 trial.

Closure of recruitment to Part A. Recruitment of trastuzumab failure patients in the 1200.89 trial has been behind the timelines. Due to this slow recruitment further continuation of recruitment was stopped.

7.3 REASONS FOR AMENDMENT 3

Recruitment for 1200.89 was ceased before it was fully completed (see above). The collection of Observation Period data was included as part of this study to increase the database of outcome results for vinorelbine added to afatinib beyond progression. Due to unfavourable benefit/risk assessment of afatinib/vinorelbine combination in BI’s pivotal phase III trial (1200.75) this combination was also stopped in trial 1200.89. Therefore collection of Observation Period data was no longer considered necessary.
Patients benefiting from treatment were continued on study medication. However once they ceased study medication they completed their study participation at the time of their last follow-up visit and did not enter the observation period.
